# Characterizing DNA methylation signatures and their potential functional roles in Merkel cell carcinoma

**DOI:** 10.1186/s13073-021-00946-3

**Published:** 2021-08-16

**Authors:** Hemant Gujar, Arjun Mehta, Hong-Tao Li, Yvonne C. Tsai, Xiangning Qiu, Daniel J. Weisenberger, Miriam Galvonas Jasiulionis, Gino K. In, Gangning Liang

**Affiliations:** 1 Department of Urology, USC Norris Comprehensive Cancer Center, University of Southern California, Los Angeles, CA USA; 2 Department of Biochemistry and Molecular Medicine, USC Norris Comprehensive Cancer Center, University of Southern California, Los Angeles, CA USA; 3 Department of Dermatology, Hunan Key Laboratory of Medical Epigenomics, Second Xiangya Hospital, Central South University, Changsha, Hunan China; 4 Department of Pharmacology, Universidade Federal de São Paulo (UNIFESP), Rua Pedro de Toledo 669 5 andar, Vila Clementino, São Paulo, SP 04039032 Brazil; 5 Department of Dermatology, USC Norris Comprehensive Cancer Center, University of Southern California, Los Angeles, CA USA

**Keywords:** DNA methylation marker, Epigenetic therapy, Epigenetic driver, MCC, MCPyV, EZHIP, KDM6B, H3K27me3, Neuroendocrine, PD1, PDL1

## Abstract

**Background:**

Merkel cell carcinoma (MCC) is a rare but aggressive skin cancer with limited treatment possibilities. Merkel cell tumors display with neuroendocrine features and Merkel cell polyomavirus (MCPyV) infection in the majority (80%) of patients. Although loss of histone H3 lysine 27 trimethylation (H3K27me3) has been shown during MCC tumorigenesis, epigenetic dysregulation has largely been overlooked.

**Methods:**

We conducted global DNA methylation profiling of clinically annotated MCC primary tumors, metastatic skin tumors, metastatic lymph node tumors, paired normal tissues, and two human MCC cell lines using the Illumina Infinium EPIC DNA methylation BeadArray platform.

**Results:**

Significant differential DNA methylation patterns across the genome are revealed between the four tissue types, as well as based on MCPyV status. Furthermore, 964 genes directly regulated by promoter or gene body DNA methylation were identified with high enrichment in neuro-related pathways. Finally, our findings suggest that loss of H3K27me3 occupancy in MCC is attributed to *KDM6B* and *EZHIP* overexpression as a consequence of promoter DNA hypomethylation.

**Conclusions:**

We have demonstrated specific DNA methylation patterns for primary MCC tumors, metastatic MCCs, and adjacent-normal tissues. We have also identified DNA methylation markers that not only show potential diagnostic or prognostic utility in MCC management, but also correlate with MCC tumorigenesis, MCPyV expression, neuroendocrine features, and H3K27me3 status. The identification of DNA methylation alterations in MCC supports the need for further studies to understand the clinical implications of epigenetic dysregulation and potential therapeutic targets in MCC.

**Supplementary Information:**

The online version contains supplementary material available at 10.1186/s13073-021-00946-3.

## Background

Merkel cell carcinoma (MCC) is a rare but aggressive neuroendocrine cancer of the skin with a high risk for recurrence and metastasis, often within 2–3 years after initial diagnosis [1]. While there are approximately 3000–3500 cases diagnosed per year in the USA, the incidence of MCC has tripled in the USA over the past four decades [2] and doubled in recent years [3]. MCC mostly affects elderly populations with a median age of diagnosis at 75–80 years old. In addition, MCC patients show overall poor outcomes with a 5-year overall survival rate of 64%. Risk factors for MCC include advanced age, exposure to UV light, fair skin, and immunosuppression (e.g., hematologic malignancy, HIV/AIDS, and solid organ transplant) [2, 4–6].

Up to 80% of MCC cases are associated with the dsDNA containing human polyomavirus 5 (HPyV5), also known as the Merkel Cell polyomavirus (MCPyV) [7, 8]. MCC tumors infected by MCPyV express the viral oncoproteins, small and large T antigen, but lack a UV-derived mutation signature [9]. On the contrary, MCPyV-negative tumors display a UV-derived mutation signature and a higher overall mutation burden [9]. Both the MCPyV-positive and negative tumors are highly immunogenic and express tumor neoantigen and viral antigens [10]. One half of all MCC tumors express PD-L1 and demonstrate the presence of tumor-infiltrating leukocytes; interestingly, these patients may have improved survival as compared to PD-L1-negative MCC patients [11].

The clinical management of MCC is challenging. MCC tumors are asymptomatic or have a benign appearance at initial presentation, leading to missed or late diagnoses [12, 13]. Pathological diagnosis requires immunostaining for neuroendocrine markers in addition to hematoxylin eosin staining [10, 14, 15]. For patients with early-stage disease, surgery and radiation are recommended to achieve local regional disease control. For patients with advanced or metastatic disease, immune checkpoint inhibition targeting the PD-1/PD-L1 pathway leads to improved survival [16–18]. However, there is no standard approach for patients who develop resistance or relapse, thus representing a large gap in clinical management.

Increasing evidence suggests that epigenetic dysregulation drives cancer progression in MCC [19–22]. Genomic analyses of MCC have revealed frequent mutations in genes regulating chromatin modification [20, 23–25]. Specifically, recent studies have demonstrated global loss of histone H3 lysine 27 trimethylation (H3K27me3) or loss of polycomb repressive complex 2 (PRC2) activity in the development of Merkel cells, although the mechanism remains unknown [20, 26]. Loss of H3K27me3 has been shown in pediatric brain tumors and may be influenced by overexpression of the PRC2 inhibitory protein EZH Inhibitory Protein (EZHIP) and/or KDM6B, an H3K27me3 demethylase [27–29].

While some DNA methylation-based biomarkers have been identified for in other aggressive skin cancers (e.g., melanoma) [30], only a small number of hypermethylated genes have been described in MCCs, namely CpG islands located at the *RASSF1A* promoter in 50% of patients [19] and *CDKN2A*^(*p14ARF*)^ (encoding tumor suppressor p14) in 42% of patients [31]. DNA hypermethylation modulates expression of both of these genes in MCC [19, 31, 32]. Negative regulation of *PD-L1* expression by DNA hypermethylation of its gene promoter region has also been recorded in many cancers [33–37]. Early studies show that epigenetic dysregulation also contributes towards immune escape and poor prognosis in MCC, including MHC class I and PD-L1 downregulation and decrease in immune cell populations [38–41]. Furthermore, preclinical studies show that epigenetic therapy with histone deacetylase (HDAC) inhibitors to reverse silencing of HLA class-1 antigen processing machinery (APM) and MHC class I chain-related proteins A and B using in vitro and mouse xenograft MCC model [39, 40].

An increased understanding of the epigenetic dysregulation of MCC biology is needed to help improve the clinical management of this rare but aggressive disease. Investigation of DNA methylation profiles in MCC may provide diagnostic and therapeutic utility in clinical management. In this study, we sought to describe the global DNA methylation landscape of MCC and characterize potential links between DNA methylation, gene expression, and MCC tumorigenesis. We have identified DNA methylation markers specific for MCC diagnosis, MCPyV status, and expression, as well as DNA methylation-based driver genes related to MCC tumorigenesis, neuroendocrine-related gene pathways, and H3K27me3 status. Our findings support further studies to understand the clinical implications of epigenetic dysregulation in MCC.

## Methods

### Sample collection

Tumor samples from 11 patients treated at the University of Southern California Keck School of Medicine and USC Norris Comprehensive Cancer Center from 2016 to 2018 were retrospectively identified and collected. All patients underwent surgical resection of primary MCC tumors, regional lymph nodes, and/or in-transit skin metastases as standard of care. Patients included nine males and two females, six non-Hispanic white patients, and five Hispanic patients; the median age was 66 (range 49–88) years old. MCC tumor samples included eight primary tumors, four lymph node metastases, and three skin metastases. Adjacent-normal tissues from five patients were used as controls. Among the 11 patients, there was one patient who was immunocompromised (history of prior kidney transplantation), while two patients were noted to have a history of second malignancy (one with metastatic breast cancer, one patient with monoclonal gammopathy of unknown significance). Staging was conducted per AJCC 8^th^ edition TNM staging system; there was one patient with stage I disease, three with stage II disease, and seven with stage III disease. Among all 11 patients who underwent surgical resection, five had recurrent disease, three remain alive and disease free, and three were lost to clinical follow-up. Additional clinical characteristics are listed in Table 1. In addition, two commercially available Merkel cell carcinoma cell lines, MS-1 and MCC13, were obtained from Millipore Sigma (St. Louis, MO) and were cultured as recommended by the supplier. This study was reviewed and approved by the institutional review board (IRB) of the University of Southern California following written informed consent from all patients.

**Table 1:** Detailed characteristics of samples used in this study

Patient	Sample_Name	Status	Site	Age	Recurrence	RACE	Gender	Stage	MCPyV (IHC)
1	MCC_s1	Normal	Skin	58	No	HISP	M		No
	MCC_s2	Metastatic (LN)	LN	58	No	HISP	M	III	No
2	MCC_s3	Primary	Skin	82	No	WHITE	F	I	Yes
3	MCC_s5	Metastatic (Sk)	Skin	53	Yes	WHITE	M	III	Yes
	MCC_s6	Primary	Skin	53	Yes	WHITE	M	III	Yes
4	MCC_s7	Metastatic (Sk)	Skin	72	Yes	WHITE	M	III	Yes
	MCC_s8	Normal	Skin	72	Yes	WHITE	M		Yes
	MCC_s9	Primary	Skin	72	Yes	WHITE	M	III	Yes
5	MCC_s10	Normal	Skin	49	Yes	HISP	M		Yes
	MCC_s11	Metastatic (Sk)	Skin	49	Yes	HISP	M	III	Yes
6	MCC_s12	Primary	Skin	66	No	HISP	M	I	No
7	MCC_s14	Primary	Skin	82	Yes	WHITE	M	III	No
	MCC_s15	Primary	Skin	82	Yes	WHITE	M	III	No
8	MCC_s16	Primary	Skin	51	No	HISP	M	II	Yes
9	MCC_s17	Primary	Skin	88	Yes	WHITE	F	II	Yes
10	MCC_s18	Normal	Skin	86	No	WHITE	M		No
	MCC_s19	Metastatic (LN)	LN	86	No	WHITE	M	III	No

### Data collection and submission

In a prospectively collected institutional review board (IRB)-approved database, MCC tumor samples were pathologically reviewed and confirmed by a certified dermatopathologist. Immunohistochemistry testing for MCPyV was performed using the CM2B4 mouse monoclonal antibody clone [42] (Santa Cruz Biotechnology, Santa Cruz, CA). Genomic DNA from MCC cell lines and FFPE tissues was extracted as described in Chopra et al [43]. Following bisulfite treatment (Zymo Research Corporation EZ DNA Methylation kit), DNA methylation data was generated using the Illumina Infinium MethylationEPIC BeadChip array at the USC Norris Molecular Genomics Core Facility [44, 45]. BeadArrays were scanned using Illumina iScan scanners and .idat files were used as input for data extraction and processing. Summarized methylated and unmethylated intensities, beta values (*β* values), and detection *p* values were generated using *minfi* in R computing language, and background correction and normalization was performed using the “noob” function in *minfi*. Data points with detection *p* value > 0.05 were masked as “NA”.

RNA extraction was performed as per the instructions from the Qiagen RNeasy Mini Kit according to the user manual (qiagen.com). The DNA methylation and gene expression data from this study can be obtained from the Gene Expression Omnibus GSE160878 and PRJNA671514. Published epidermis and dermis DNA methylation data from apparently healthy individuals above the age of 50 were obtained from GEO (GSE51954) for cell type DNA methylation comparisons [46]. DNA methylation data were obtained for primary small cell lung carcinomas (SCLCs )[47], normal lymph node DNA methylation data from GEO (GSE73549) [48], normal tibial neuron from ENCODE (ENCSR551DKY, ENCSR729VBL, ENCSR061NRX, ENCSR039CGW), epilepsy brain tissues from GEO (GSE111165), lung carcinoids from GEO (GSE118133), pancreatic cancer from GEO (GSE117852), pediatric high-grade glioma (pHGG) from E-MTAB-5552, and prostate cancer from GDC.

### DNA methylation analysis

We removed primary tumor samples containing higher than 50% white blood cell contamination using the LUMP (leukocytes unmethylation for purity) assay [49]. Probes with mean DNA methylation β-values less than 0.05 (5%) across the entire sample set were considered as background noise and removed from the analysis. Probes with DNA methylation associated with gender and age, as well as those related to polymorphisms, were also removed from our analysis [50, 51]. Student’s *t* test was performed to identify significantly different methylation β-values of each probe between MCPyV-positive and MCPyV-negative samples. P-value correction (false discovery rate, FDR) was performed using the *p.adjust* function in R [52]. Probes with mean methylation β-value difference of > 0.4 or < − 0.4 between two sample groups (i.e., MCPyV-positive vs MCPyV-negative) at FDR < 0.05 were selected. Probes with the greatest β-value deviation between adjacent-normal, primary tumor, metastatic skin tumor and metastatic lymph node tissues were selected with ANOVA p-value < 0.05 and standard deviations (SD) of the mean of groups > 0.25. Heatmap representation was generated using the R package *ComplexHeatmap* [53]). The utility of these probes in differentiating tumor samples was shown using principle component analysis graph with generic R functions *prcomp* and *ggplot2* [54].

### Probe annotations and pathway analyses

We identified EPIC DNA methylation probes located in promoter and gene body regions and classified the remaining probes as intergenic. Probe annotations were obtained from the Infinium MethylationEPIC manifest (illumina.com). Hypergeometric test for determining the enrichment of probes in promoters, gene body, and intergenic regions was performed using the *phyper* function in R. GO annotation was performed using the R package *RDAVIDWebService* or *enrichGO* [55], and data were presented using the R package *clusterProfiler* [56].

### RNA sequencing (RNA-seq)

Total RNA was extracted, and RNA libraries were prepared using the TruSeq Stranded Total RNA kit according to the manufacturer’s recommended protocol (illumina.com). Total RNA sequencing was performed on the NextSeq 500 instrument (Illumina). Single- or paired-end sequencing reads of ~ 75 bp in length were obtained and were cleaned using trimmomatic [57]. Cleaned reads were aligned to human genome hg38 using STAR aligner [58]. Count data was generated using featureCounts [59] and normalized using EdgeR [60]. Two cell line replicates were performed. Expression data from cell lines was compared with normal whole skin RNA expression (GSE130955). The relative expression in cell lines was compared with relative DNA methylation in MS1 and MCC13 cell lines with respect to normal skin.

### Data access

GSE160878 for DNA methylation and PRJNA671514 for gene expression (this study). GSE51954 for published epidermis and dermis DNA methylation data from apparently healthy individuals over the age of 50 [46].

GSE73549 for normal lymph node DNA methylation data [48].

ENCSR551DKY, ENCSR729VBL, ENCSR061NRX, and ENCSR039CGW for tibial neuron DNA methylation data.

GSE111165 for epilepsy brain tissue DNA methylation data.

GSE118133 for lung carcinoid DNA methylation data.

GSE117852 for pancreatic cancer DNA methylation data.

GDC for prostate cancer DNA methylation data.

E-MTAB-5552 for pediatric high-grade glioma (pHGG) DNA methylation data.

GSE130955 for gene expression in normal skin tissues [61, 62].

## Results

### DNA methylation profiles in MCC specimens and differentially methylated loci involve multiple pathways

DNA methylation changes of only a limited set of genes have been identified in MCC [38]; thus, we investigated genome-scale DNA methylation profiling of primary MCC specimens, including primary tumors, adjacent-normal tissues, metastatic skin tissues, and metastatic lymph nodes across 11 patients, using the Illumina MethylationEPIC DNA methylation BeadArray system (Additional file 1: Figure S1). The DNA methylation data were filtered to remove data from probes that are (1) linked to known polymorphisms, (2) located on the X- and Y-chromosomes, and (3) related to aging (Additional file 1: Figure S1). In addition, the data from two metastatic lymph nodes were omitted as these were shown to have < 50% purity after testing for infiltration of normal cells or leukocytes caused by inflammation using LUMP assay (Additional file 1: Figure S2 and the “Methods” section).

**Fig. 1:**
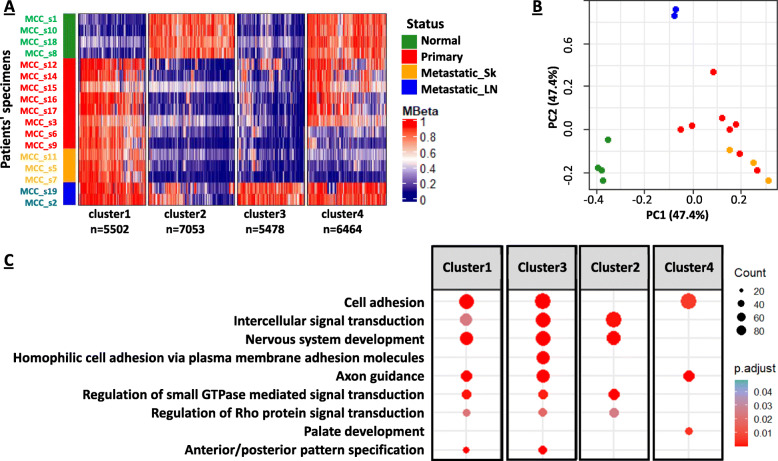
Differential DNA methylation and related pathways in MCC. **A** Illumina Infinium MethylationEPIC BeadChip revealed DNA methylation profiles in the four groups (Cluster 1-4) by comparing Normal, Primary, Metastatic_Sk (Sk for skin), Metastatic_LN (LN for Lymph Node). DNA methylation in these groups was compared using ANOVA. Using *P* < 0.05 and SD of the mean > 0.25, we obtained 24,497 probes. Using hierarchical clustering of probes, four clusters were identified **B** PCA plot showing that the selected probes separated samples in three groups: Normal, Metastatic lymph node (LN), and Primary and Metastatic_Sk. **C** Differentially methylated genes in clusters 1-4 were annotated using GO terms for biological process. GO analysis for biological terms using the R function *RDAVID* was performed and GO terms were reported

We performed ANOVA-based multiple comparison testing of the remaining 700,268 probes to find differentially methylated probes across the sample collection (Table 1). A total of 181,429 probes were significantly differentially methylated (*p* < 0.05) between the four sample groups. Probes showing the highest variation of DNA methylation between the four groups were retained. Using a standard deviation (SD) of the four means greater than 0.25, 24,497 probes were selected (Fig. 1).

Unsupervised clustering of the 24,497-probe set revealed four clusters. Cluster 1 (*n* = 5502 probes) showed cancer-specific DNA hypermethylation in all tumor tissues (primary, metastatic skin, and metastatic lymph node) compared to adjacent-normal tissues. Cluster 2 (*n* = 7053 probes) showed DNA hypomethylation in primary tumors and metastatic skin tissues, but not in metastatic lymph nodes when compared to adjacent-normal tissues. Cluster 3 (*n* = 5478 probes) showed DNA hypermethylation only in metastatic lymph nodes in comparison to the other three sample groups. Cluster 4 (*n* = 6464 probes) displayed DNA hypomethylation in metastatic skin and primary tumors (Fig. 1A). PCA analysis demonstrated separation of tissue samples into three groups: primary tumors (red) with metastatic skin tissues (orange), adjacent-normal tissues (green), and metastatic lymph nodes (blue) (Fig. 1B).

The unique Cluster 3 DNA hypermethylation profiles found in metastatic lymph nodes let us to question whether this might be due to tissue-specific DNA methylation patterns in lymph nodes or was derived from primary tumors. To address this, we re-clustered the data after adding EPIC DNA methylation data of three primary normal lymph node tissues [48] (Additional file 1: Figure S3A). Indeed, the DNA methylation profiles of normal lymph nodes were similar to adjacent-normal skin tissues, while the Cluster 2 probes that displayed specific DNA hypomethylation in primary tumors and metastatic lymph nodes were unique from the regions displaying DNA methylation in normal lymph nodes (Additional file 1: Figure S3A and B). Thus, the Cluster 3-specific DNA hypermethylation patten is unique to metastatic lymph nodes involved with MCC.

MCC patients are sensitive to immunotherapy (immune checkpoint inhibitors) [63, 64], and recent studies have suggested that DNA demethylation and reactivation of transposon elements (TEs), such as endogenous retroviruses (ERVs), can lead to up-regulation of tumor cell immune response (viral mimicry) and increase T cell infiltration [65–67]. In order to determine TE DNA methylation status in each cluster, we analyzed the DNA methylation status of 1286 TE probes on the Infinium MethylationEPIC array (Additional file 1: Figure S1). TEs showed very similar DNA methylation patterns across the four clusters, even after unsupervised clustering of the panel of 1286 TE probes alone (Additional file 1: Figure S3C). Thus, TE DNA methylation is distributed across all four clusters and no TE-specific DNA methylation patterns are identified (Fig. 1).

The differentially methylated probes in all four clusters were located on promoter, gene body, or intergenic regions. Enrichment analysis using *phyper* function in R programming language showed that while all clusters contain probes located in intergenic regions, gene body probes were enriched in Clusters 2, 3, and 4. In addition, promoter or gene body DNA hypermethylation is found only in Cluster 3 probes in metastatic lymph nodes (Additional file 1: Figure S3D). Promoter DNA methylation is negatively correlated with gene expression and gene body DNA methylation is positively correlated with gene expression [68, 69]. Meanwhile, DNA methylation in intergenic regions may correlate with chromatin instability and regulation of functional elements, such as enhancers [70, 71]. Thus, the distribution of probes on various genic regions and their DNA methylation states may provide clues regarding potential gene activity.

GO analysis for biological terms was performed for all differentially methylated probes in clusters 1- 4 using *RDAVIDWebService*, and these data suggest that DNA methylation alterations in MCC involve in multiple pathways, including in cell adhesion, signal transduction, and nervous system development, all of which may directly participate in MCC tumorigenesis (Fig. 1C). Pathway analyses based probe location (promoter or gene body) suggests that gene body DNA methylation may drive changes in cell adhesion, signal transduction, and nervous system development (Additional file 1: Figure S3E). Taken together, we identified four clusters of cancer-specific DNA hypermethylation (Clusters 1 and 3) and hypomethylation (Clusters 2 and 4) profiles, some of which are also specific to metastatic MCC tumors and may play a critical role in pathways relevant to tumor progression in MCC (Fig. 1C).

### MCPyV-specific DNA methylation patterns in MCC tumors

Among approximately 80% of patients, MCC is associated with the oncogenic virus MCPyV. Approaches to detect MCPyV include PCR for virus-specific sequences, as well as immunohistochemical and serologic testing for viral oncoproteins. However, to date, no epigenetic biomarker has been established as the consensus test for determining MCPyV status. As a result, MCPyV infection in patients may be easily missed [7, 8] or left unchecked. While it is thought that MCPyV status impacts MCC patient prognosis [72], how MCPyV status may alter clinical decision-making is also controversial [1, 73]. As such, improved diagnostic testing to delineate MCPyV status may be important not only towards improved understanding of MCC biology, but also towards clinical management. Based on immunohistochemistry, five primary tumors and three metastatic skin tumors were MCPyV-positive, while three primary tumors and two metastatic lymph node tumors were MCPyV-negative.

**Fig. 2:**
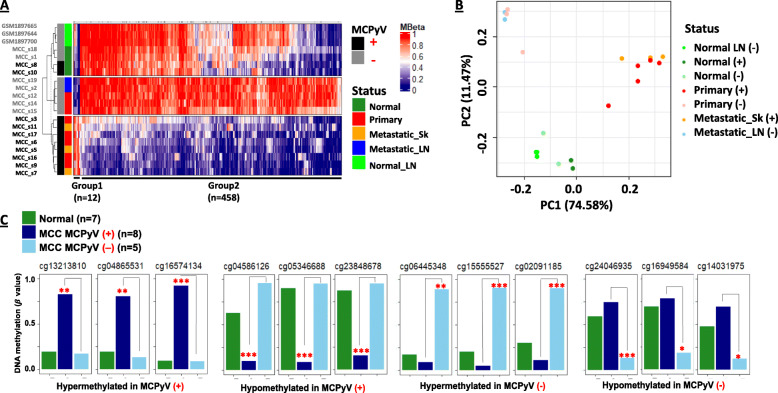
DNA methylation differences based on MCPyV status in MCC. **A** Comparison of DNA methylation in MCPyV-positive vs MCPyV-negative samples using Student’s *t* test revealed 470 loci that were differentially methylated FDR-adjusted *p* < 0.05 and minimum delta change of ± 0.4. Heatmap shows DNA methylation profiles in MCC tumor, adjacent-normal skin, and normal lymph node samples. **B** These probes characterized samples in MCPyV-positive (red/orange) and MCPyV-negative (pink/sky blue) groups in primary (red/ pink), metastatic skin (orange), and metastatic lymph node (sky blue) samples. Adjacent-normal skin (forest green and light green), normal lymph node (green) samples based on PCA plot. **C** Mean DNA methylation from selected loci differentially methylated in MCPyV positive and MCPyV negative samples based on hyper or hypomethylated in MCPyV positive and MCPyV negative is recorded and depicted in bar-graphs. Adjusted *p* value (FDR) significance thresholds are indicated: 0.01 < *p* < 0.05 (*); 0.001 < *p* < 0.01 (**); *p* < 0.001 (***)

We determined whether DNA methylation profiles differed between MCPyV-positive (*n* = 8) and MCPyV-negative tumor tissues (*n* = 5) using supervised clustering of the EPIC DNA methylation data for these samples. Using FRD-adjusted *P* < 0.05 and delta *β* value differences > 0.4 or < − 0.4, we identified 470 probes (*n* = 12 in Group 1 and *n* = 458 in Group 2) showing significant differential DNA methylation between MCPyV-positive and MCPyV-negative tumors (Fig. 2A). Interestingly, the DNA methylation profile of MCPyV-positive tumors was independent from adjacent-normal skin or normal lymph node tissues irrespective of the MCPyV status in normal tissues (Fig. 2A). PCA analysis showed that the MCPyV-positive (red and orange) and MCPyV-negative (pink and light blue) tumors separated into two groups and do not overlap with normal tissues regardless of MCPyV status (green and light green) (Fig. 2B). In addition, we further refined the top 12 cancer-specific DNA methylation markers, a combination of both hypermethylated and hypomethylated loci, from this group that could most clearly distinguish MCPyV status among the MCC specimens (Fig. 2C). The top 10 significant loci in each group are listed in Additional file 2: Table S1.

### Differential DNA methylation among skin cell types and cancer cells of origin

**Fig. 3:**
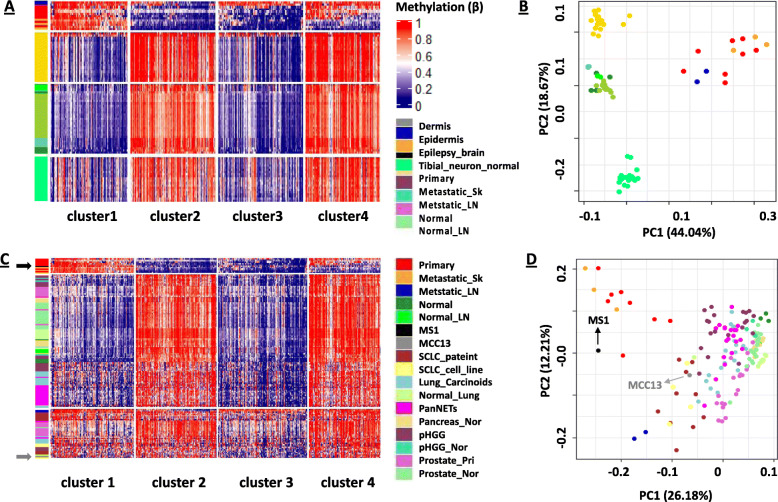
Differential DNA methylation in skin cell subtypes and cancer cells of origin. **A** MCC-specific DNA methylation (Fig. 1A) was compared to DNA methylation data from epidermis, dermis, tibial neuron, and epilepsy brain tissues. **B** PCA plot showing MCC was well separated from dermis, epidermis, tibial neuron, and epilepsy brain tissues. **C** Unsupervised clustering the MCC-specific DNA methylation profiles (Fig. 1A) with DNA methylation data from SCLC tissues, SCLC cell lines, MCC cell lines (MS1 and MCC13), lung carcinoids, pancreatic tumors, prostate tumors, and pediatric high-grade gliomas (pHGG). **D** PCA plot of Normal tissue, primary MCC tumors, with SCLC, SCLC cell lines, MCC cell lines (MS1 and MCC13), lung carcinoids, pancreatic tumors, prostate tumors, and pediatric high-grade gliomas (pHGG)

Merkel cells are mechano-sensory receptors that are required for soft touch response, have neuro-endocrine features, and are in the basal layer between dermis and epidermis, however, there is controversy over whether these are the true cells of origin for MCC [74, 75]. Depending on the location of primary tumors and skin metastases, the collected tumor samples from this study could contain differing proportions of epidermal, dermal tissue, and Merkel cells, thereby potentially resulting in a sampling bias that may affect our analyses due to cell-type-specific DNA methylation profiles. To determine the potential consequences of dermal, epidermal, and neuro-like cell contamination among our resected MCC tumor tissues, we obtained and analyzed publicly available Infinium HumanMethylation450 (HM450) BeadArray DNA methylation data for primary epidermis and dermis samples from sun-exposed and sun-protected body sites of 20 individuals over 60 years old (GSE51954) [46], as well as tibial neuron (ENCSR551DKY, ENCSR729VBL, ENCSR061NRX, ENCSR039CGW) and epilepsy brain tissues (GSE111165). Specifically, we compared the epidermis, dermis, and neuro-like tissues (tibial neuron and epilepsy brain) DNA methylation profiles of the probes used for clustering (Fig. 1) to the MCC data. A total of 24,497 probes were originally used for clustering and 13,460 of these are also represented in the HM450 array data. After performing cluster analysis using the 13,460 shared probes across the MCC sample panel, the same four clusters remained. Interestingly, we noticed that epidermal, dermal, and neuron-like tissues have their own unique DNA methylation profiles after including these samples in the clustering analysis (Fig. 3A). Dermal, epidermal and neuron-like samples were clearly different from MCC tissues based on PCA plot analyses (Fig. 3B); therefore, the DNA methylation profiles identified in Clusters 1–4 (Figs. 1A and 3A) are likely MCC-specific.

In addition to tumor cell purity, cancer cell of origin is also influential in characterizing DNA methylation profiles and may result in misleading findings of cancer cell type. Both MCC and small-cell lung carcinoma (SCLC) are neuroendocrine tumors and share cytological and histochemical similarities [76]. In addition to the challenges in characterizing poorly differentiated neuroendocrine tumors, MCC and SCLC can present with both lymph node and skin metastases. As a result, MCC and SCLC may be misdiagnosed [77–79], and this may alter treatment options for the patient.

Cells of origin questions also persist for human MCC cell lines [80]. DNA methylation data can be used to identify the cell of origin [81], therefore, we took advantage of specific DNA methylation profiles based on tissue, cell, or cancer type to analyze the cell of origin for human MCC13 and MS-1 MCC cell lines. We compared MCC13 and MS-1 DNA methylation patterns with our patient MCC samples (Fig. 1A). In addition, we compared MCC EPIC DNA methylation data with HM450 DNA methylation data from primary SCLC and corresponding adjacent-normal lung tissues generated by Poirier et al. [47] and other potential neuroendocrine or neuro-like tumors such as lung carcinoids (GSE118133; *n* = 18), pancreatic tumors (GSE117852, *n* = 20), prostate tumors (GDC, *n* = 20), and pediatric high-grade gliomas (pHGG) (E-MTAB-5552; *n* = 20). Unsupervised clustering of the MCC patient samples, MCC cell lines and other tumor types using the panel of 13,460 probes shared between the EPIC and HM450 arrays showed that MCC and other tumor types have unique DNA methylation profiles. Interestingly, the MS1 cell line clustered with patient MCC samples, while the MCC13 cell line clustered with the SCLC samples and SCLC cell lines but not the other cancer types. In addition, adjacent-normal skin and adjacent-normal lung tissues display similar DNA methylation profiles (Fig. 3C). PCA analysis showed that the primary MCC, metastatic skin, SCLC, other cancer types, adjacent-normal skin, and normal lung tissue samples clustered separately. In addition, PCA analyses also showed that the MS1 cell line clustered with MCC patient samples, while the MCC13 cell line clustered with SCLC samples and SCLC cell lines (Fig. 3D). This finding suggests that MCC cell lines have unique cells of origin with MCC13 cells likely derived from metastatic SCLC cells, as previously hypothesized [80]. Thus, DNA methylation may be a novel approach to help identify cancer cell of origin for aggressive neuroendocrine malignancies.

### Impact of DNA methylation on gene regulation in MCC

Most cancer-specific DNA methylation alterations are passage effects and do not result in altered gene expression [69, 82, 83]. Only a small portion of DNA methylation alterations correlate with gene expression changes; specifically, promoter DNA and gene body DNA methylation are negatively and positively associated with gene expression, respectively [68, 84]. Due to the limited tissue availability of this rare cancer, we were unable to perform RNA expression analyses in patient samples and instead focused on RNA sequencing (RNA-seq) of MCC13 and MS1 MCC cell lines to characterize the extent to which MCC DNA methylation may affect gene expression.

**Fig. 4:**
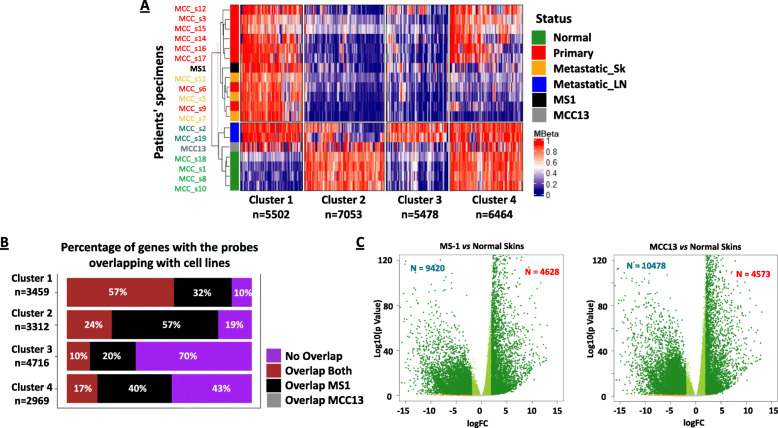
DNA methylation and RNA expression in MCC cell lines. **A** Unsupervised clustering of probes based on MCC-specific DNA methylation compared to MS1 and MCC13 cell lines. **B** Differentially methylated genes in the four clusters were overlapped with MS1 and MCC13. Percentage of genes showing DNA methylation similar to cell lines were recorded. **C** Volcano plot for gene expression by RNA-seq in MS1 and MCC13 cell lines compared to normal skin samples

First, we clustered the MCC DNA methylation data with MCC13 and MS1 cell lines included. The MS1 DNA methylation profiles were similar to MCC tumors, while the MCC13 DNA methylation profiles clustered with adjacent-normal MCC tissues (Fig. 4A). Next, we determined the extent to which the MCC cell line data overlapped with each MCC cluster (Fig. 4B). Both cell lines showed substantial similarity with the Cluster 1-specific probes, whereas the majority of Cluster 3 probes did not overlap with the MCC cell lines. Interestingly, we did not identify MCC13-specific overlap with any of the four clusters, but in contrast, there was overlap with MS1 among four clusters. We identified MCC-specific promoter or gene body DNA methylation alterations in 8885 of 14,456 genes (61%) in one or both MCC cell lines.

**Fig. 5:**
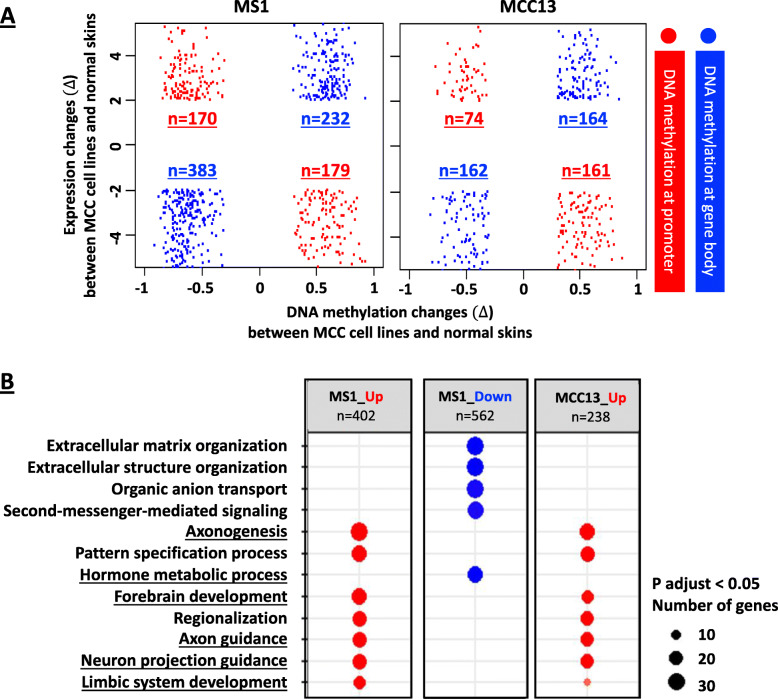
Genes regulated by DNA methylation in MCC. **A** DNA methylation changes in MCC cell lines vs normal skin samples were plotted on *x*-axis (∆*β* > 0.3 or ∆*β* < − 0.3) and expression levels were plotted on the *y*-axis (fold change > 2 or < − 2). Probe location on promoter (red) and gene body (blue) is shown on the scatter plot. **B** GO analysis for biological terms for genes regulated by DNA methylation using the R function *enrichGO* was performed and GO terms were reported

Second, we uncovered dramatic expression differences when comparing gene expression of MS1 and MCC13 cells to normal skin tissues (GSE130955) [61, 62] (Fig. 4C), suggestive of widespread epigenetic dysregulation in MCC. After integrating the MS1 and MCC13 DNA methylation and RNA-seq data for the panel of 8885 genes, we identified 968 genes (11%) that are directly regulated by DNA methylation (Fig. 5A, Additional file 2: Table S2 and S3). These genes include those upregulated by promoter DNA hypomethylation (171 genes in MS1 cells and 74 genes in MCC13 cells) and gene body DNA hypermethylation (232 genes in MS1 cells and 164 genes in MCC13 cells), as well as genes downregulated by promoter DNA hypermethylation (179 genes at MS1 cells and 161 genes in MCC13 cells) or gene body DNA hypomethylation (383 genes in MS1 cells and 162 genes in MCC13 cells) (Fig. 5A).

GO analysis for biological terms on genes regulated by DNA methylation showed enrichment in neuroendocrine-related pathways including axonogenesis, hormone metabolism process, forebrain development, axon guidance, neuron projection guidance, and limbic system development (Fig. 5B). This finding suggests that DNA methylation alterations may directly contribute to the neuroendocrine features present in MCC.

### Identification of DNA methylation regulated genes involved in MCC tumorigenesis, neuroendocrine status, and MCPyV infection

Aberrant DNA methylation aberrations have been described in most types of human cancers [85]. However, most of the defined alterations appear to be passenger events that do not lead to gene expression changes [83, 86, 87]. Understanding the relationship between DNA methylation alterations and gene expression changes will provide not only a functional DNA methylation marker for gene expression status, but also a potential therapeutic biomarker, especially for DNA methylation inhibitors [88]. We recently demonstrated that epigenetic alterations are more frequent than genetic alterations in regulating gene expression, and this may be identified by correlating gene expression with DNA methylation and/or nucleosome accessibility of gene promoters or gene bodies [68, 69, 83, 87, 89].

**Fig. 6:**
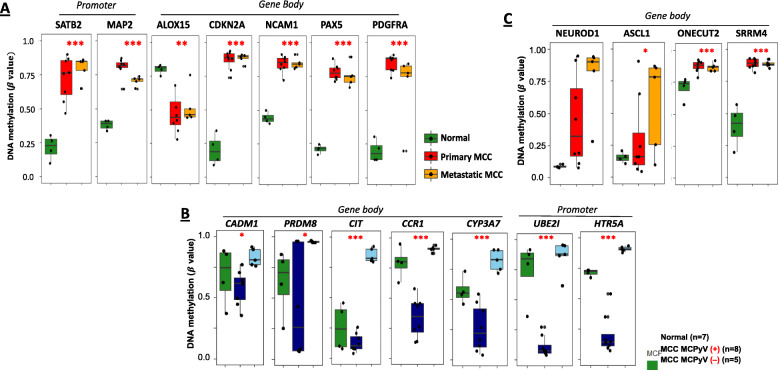
The selected genes regulated by DNA methylation and those related to MCC tumorigenesis, MCPyV, and neuroendocrine status. **A** The genes related to A) MCC tumorigenesis, **B** MCPyV status, and **C** neuroendocrine signatures were identified by comparing DNA methylation of normal, primary, and metastatic (skin and lymph node) MCC samples. The data recorded and depicted in bar-graphs. Adjusted *p* value (FDR) significance thresholds are listed: 0.01 < *p* < 0.05 (*); 0.001 < *p* < 0.01 (**); *p* < 0.001 (***)

We queried our list of cancer-related genes regulated by DNA methylation in MCC (Fig. 5A) to identify MCC-specific DNA methylation regulated genes which mainly dependent on expression status as existing MCC biomarkers, MCPyV-specific biomarkers, and neuroendocrine-specific genes. First, we identified epigenetic regulation of *SATB2*, *MAP2*, *ALOX15*, *CDKN2A*, *NCAM1*, *PAX5*, and *PDGFRA* in MCC. Interestingly, these seven genes have been described as diagnostic and/or prognostic markers for MCC based on RNA or protein expression [90–95]. Indeed, *SATB2*, *MAP2*, and *ALOX15* were previously reported as down-regulated in MCC. Our data suggest that SATB2 and MAP2 downregulated expression is correlated with promoter DNA hypermethylation, while downregulated ALOX5 expression correlates with gene body DNA hypomethylation (Fig. 6A) [90–92]. Furthermore, overexpression of CDKN2A, NCAM1, PAX5, and PDGFRA in MCC can be indicated by DNA hypermethylation of their gene bodies (Fig. 6A) [93–95]. Thus, their DNA methylation status also provides clinically relevant MCC biomarkers.

Second, *CADM1* and *PRDM8* expression have been previously studied as putative biomarkers for MCpyV status in MCC [21, 96]. We found that and *CADM1* and *PRDM8* expression positively correlated with DNA methylation in their gene bodies. We then overlapped the MCPyV-specific DNA methylation probes (Fig. 2A) with the genes that were regulated by DNA methylation in MCC (Fig. 5A). In doing so, we not only identified DNA methylation markers that were strongly correlated with MCPyV status (Fig. 2C), but we also identified 10 genes whose DNA methylation status correlated with gene expression (Fig. 5A) (Additional file 2: Table S4). The top five genes displaying significant DNA methylation differences between MCPyV-negative and MCPyV-positive MCC tumors are shown and compared to *CADM1* and *PRDM8* (Fig. 6B). Our list of MCPyV-specific genes shows substantial DNA methylation differences and outperforms *CADM1* and *PRDM8*, indicating that DNA methylation and gene expression curated biomarkers are effective and specific for MCPyV status in MCC.

Third, neuroendocrine signatures based on *NEUROD1* and *ASCL1* [97] expression are not only a feature of MCC but are also used for diagnostic and prognostic purposes [1]. For both *NEUROD1* and *ASCL1*, we found that gene body DNA methylation status correlates with gene expression (Fig. 5A). In addition, we also identified two well-known neuroendocrine genes, *ONECUT2* and *SRRM4* [98, 99], that demonstrated cancer-specific gene body DNA methylation alterations (Fig. 6C), and which also positively correlated with gene expression in MCC (Fig. 5A). Taken together, these DNA methylation markers not only correlate with their expression status, but are also potential targets of epigenetic therapy.

### DNA methylation alterations influence global loss of histone H3 lysine 27 trimethylation in MCC

**Fig. 7:**
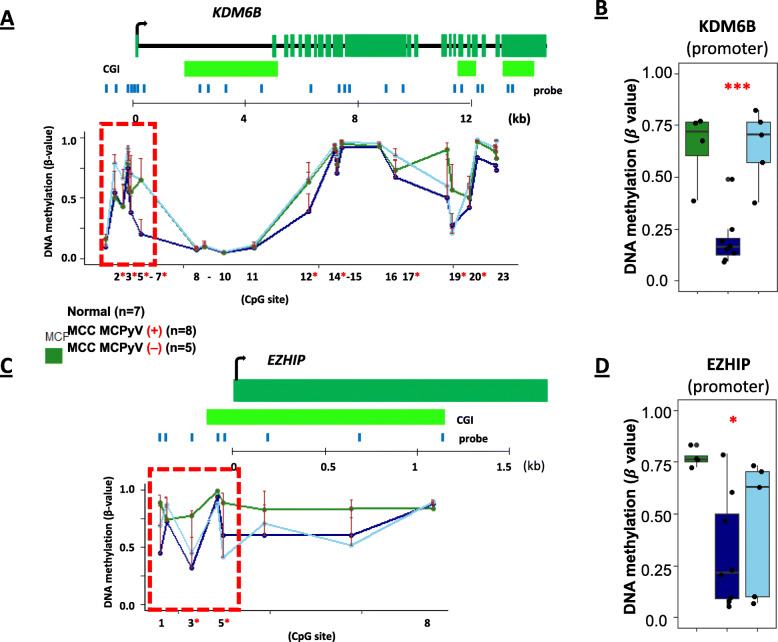
DNA methylation in genes involved in H3K27me3demethylation. **A**, **C** Plots displaying all probes for *KDM6B* and *EZHIP*, arranged by genomic context showing promoter DNA hypomethylation after comparisons of normal, MCPyV-positive, and MCPyV-negative samples. **B**, **D** Changes in *KDM6B* and *EZHIP* promoter DNA methylation-based on box plot analyses. Adjusted *p* value (FDR) significance thresholds are listed: 0.01 < *p* < 0.05 (*); 0.001 < *p* < 0.01 (**); *p* < 0.001 (***)

Global loss of H3K27me3 occupancy has been reported in MCC [20, 100]. In brain tumors, global loss of H3K27me3 may be explained by KDM6B over-expression, leading to H3K27me3 demethylation, and/or EZHIP over-expression that leads to inhibition of EZH2, resulting in global loss of H3K27me3 [27–29]. However, these data have not been shown in MCC. We found that *KDM6B* promoter DNA hypomethylation (CpG site from 2 to 7) was enriched in MCPyV-positive MCCs (Fig. 7A, B) and correlates with *KDM6B* overexpression in MCC (Additional file 2: Table S2 and S3). In addition, *EZHIP* promoter DNA hypomethylation (CpG site 1–5) and subsequent gene expression was also identified in MCCs regardless of MCPyV status (Fig. 7C, D). These results suggested that the up regulation of *KDM6B* and *EZHIP* by DNA hypomethylation in their promoters may contribute to global loss of H3K27me3 in MCC.

### The potential role of DNA methylation in modulating immune responses in MCC

MCC sensitivity to immune checkpoint inhibitor treatment is mainly dependent on T cell infiltration and PD-1 (PDCD1) and PDL-1expression. Interestingly, expression of PD-1, but not PDL-1, is associated with response to immunotherapy [101]. In addition, *PD-1* and *PDL1* DNA methylation is associated with survival outcomes in MCC and melanoma [33, 38]. We measured *PD-1* and *PDL-1* DNA methylation in MCC tumors, metastatic lymph nodes, and adjacent normal skin tissues. We observed significant *PDL-1* promoter DNA hypermethylation (CpG site 3) (Additional file 1: Figure S4A) and PD-1 promoter DNA hypomethylation (CpG site 1–5) (Additional file 1: Figure S4B) in primary MCCs and metastatic lymph nodes. Although it is yet unclear if *PD-1* and *PDL-1* promoter DNA methylation influences gene expression, our findings suggest that downregulated PDL-1 expression and up-regulated PD-1 expression may be due to DNA methylation changes in their promoters. This knowledge may have clinical relevance in helping identify patients which benefit from immune checkpoint inhibitors [33, 38].

## Discussion

MCC is a rare but challenging malignancy with poor clinical outcomes and may have a benign appearance at initial presentation, leading to missed or late diagnosis [12, 13]. Improved understanding of the biology of MCC, including the impact of MCPyV infection, neuroendocrine features, epigenetic alteration, and immune response-related immunotherapy are critical to improving clinical management of MCC [12, 13]. MCC can also be diagnostically challenging for the clinical pathologist. Testing for the presence of MCPyV and neuroendocrine markers has improved sensitivity and specificity; however, these tests have been dependent on protein and RNA expression from primary specimens [14, 102, 103]. In addition, 20% of MCC tumors are MCPyV-negative and SCLC metastases may be mistaken with MCC pathology at diagnosis [102, 104].

In this study, we have compared the DNA methylation profiles among primary MCC, metastatic MCC, and normal-adjacent tissues. Our analyses revealed four clusters of DNA methylation profiles that can distinguish these samples: MCC-specific hypermethylation regardless of metastatic status (Cluster 1); hypermethylation-specific for MCC lymph node metastases (Cluster 3); MCC-specific hypomethylation that excludes lymph node metastases (Cluster 2); and MCC-specific hypomethylation unique to MCC skin metastases (Cluster 4). These DNA methylation patterns are unique and independent of MCPyV status, and thus may have potential diagnostic and prognostic value in the management of MCC. Unexpectedly, we also identified a panel of DNA methylation markers that distinguish MCPyV infected tumors from non-infected tumors. However, the mechanisms as to how MCPyV actually affects epigenetic alterations in MCC are yet to be determined.

SCLC shares several clinical and pathological features with MCC, and it has been reported that some metastatic MCCs may be derived from SCLCs [77–79]. Because of unique DNA methylation signatures based on tissue, cell, cancer, and cancer cell of origin, we compared DNA methylation profiles between these two types of malignancies. Indeed, DNA methylation profiles of MCC and SCLC tumors are unique and can be used to identify cell of origin. Intriguingly, we found that the MCC13 cell line, considered a Merkel cell “variant,” displayed DNA methylation more similar to SCLCs than MCCs, thereby suggesting that SCLC may be the true origin of this cell line.

Our DNA methylation data also provides potential evidence that aberrant DNA methylation may contribute to MCC tumorigenesis. Gene ontology analysis has indicated potential dysregulation of cell proliferation, neurological development, and hormone regulation pathways. Notably, these pathways are also enriched in genes regulated by promoter or gene body DNA methylation, thus strengthening the possibility that DNA methylation is directly involved in MCC tumorigenesis.

Although global H3K27me3 loss has been reported in MCC [20, 100], this mechanism has not been well studied. Using DNA methylation primary MCC specimens and expression data from MCC cell lines, we show that *KDM6B* and *EZHIP* over-expression by promoter DNA hypomethylation may drive global H3K27me3 loss in MCC. H3K27me3 loss may represent a target for epigenetic therapy based on PRC2, HDAC, and DNA methylation inhibition in other malignancies [105–107], and this may prove an important option in MCC as well [39, 40]. Our findings provide further rationale for clinical trials of epigenetic cancer therapy in MCC. In addition, the specific interaction between epigenetic modification and immunosuppressive pathways should be further explored [66, 88]. When considering that *PD-1* and *PD-L1* expression is associated with immunotherapy response [101] and that their gene promoter DNA methylation levels can potentially predict their expression status, it seems that therapies to modulate epigenetic changes in MCC may help enable improved responses to immunotherapy.

Aberrant DNA methylation is a common event in most malignancies but most of the defined alterations appear to be passenger events that do not actually lead to gene expression changes [83, 85–87]. In this study, by combining DNA methylation from MCC patient specimens and gene expression data from MCC cell lines, we have identified over 900 genes that are directly regulated by promoters or gene body DNA methylation. The functional roles of these genes will need to be evaluated in further studies, especially testing for potential therapeutic or epigenetic therapy efficacy using in vitro and/or in vivo systems. Prior studies have already analyzed RNA or protein expression from some of the genes in this group as biomarkers to evaluate relevant pathways unique to MCC, MCPyV infection, and neuroendocrine features. The established correlation between DNA methylation and gene expression in these genes suggests that these DNA methylation markers can be used in place of RNA- or protein-based gene expression markers in the clinic.

## Conclusions

Taken together, our identification of MCC-specific DNA methylation markers may help provide the foundation for novel methodologies in the clinical diagnosis and prognostication of MCC. It should be noted that DNA is especially stable and easy to obtain from patients in the clinical setting, while DNA methylation markers are easily detected by various global or locus-specific assays [108]. We believe that this approach also could lead to more efficacious, personalized management of MCC based on patient-specific genetic/epigenetic alterations. Although our DNA methylation analyses have identified novel regions of interest that may serve to help MCC in the clinic, these findings are limited by low sample size, and larger cohorts are needed to validate these findings and assess their clinical relevance in the future studies.

## Supplementary Information

**Additional file 1 **Figure S1: Outline for procedure and results obtained in this study. Figure S2: LUMP assay. Figure S3: DNA methylation pattern in normal lymph node and TE methylation status in MCC specimens. Figure S4: DNA methylation in *PD-1* and *PDL-1*.

**Additional file 2.** Supplemental Table 1: Top 10 differentially methylated probes based on MCPyV and DNA methylation status in four groups. Supplemental Table 2: List of genes regulated by DNA methylation in the MS1 cell line. Supplemental Table 3: List of genes regulated by DNA methylation in the MCC13 cell line. Supplemental Table 4: List of genes regulated by DNA methylation related to MCPyV status.

## Data Availability

GSE160878 for DNA methylation and PRJNA671514 for gene expression (this study): Gangning Liang, Hemant Gujar, Arjun Mehta, Yvonne Tsai, Xiangning Qiu, Daniel J. Weisenberger, Gino K. In: Characterizing DNA Methylation Signatures and Their Potential Functional Roles in Merkel Cell Carcinoma; GSE160878 (https://www.ncbi.nlm.nih.gov/geo/query/acc.cgi?acc=GSE160878) and PRJNA671514 (https://www.ncbi.nlm.nih.gov/sra/?term=PRJNA671514) [109]. GSE51954 for published epidermis and dermis DNA methylation data from apparently healthy individuals over the age of 50 [46]. GSE73549 for normal lymph node DNA methylation data [48]. The tibial neuron DNA methylation data was downloaded from ENCODE portal [110] (https://www.encodeproject.org/) with the following identifiers: ENCSR551DKY, ENCSR729VBL, ENCSR061NRX, ENCSR039CGW. GSE111165 for epilepsy brain tissue DNA methylation data [111]. GSE118133 for lung carcinoid DNA methylation data [112]. GSE117852 for pancreatic cancer DNA methylation data [113]. GDC for prostate cancer DNA methylation data [114]. E-MTAB-5552 for pediatric high-grade glioma (pHGG) DNA methylation data [115]. GSE130955 for gene expression in normal skin tissues [61, 62].
