# Supplementary material for: Characterizing DNA methylation signatures and their potential functional roles in Merkel cell carcinoma
Source: Genome Med. 2021 Aug 16;13:130. doi: 10.1186/s13073-021-00946-3 (PMC8365948; doi:10.1186/s13073-021-00946-3)
Supplement: Supplementary file 1 — Additional file 1 Figure S1: Outline for procedure and results obtained in this study. Figure S2: LUMP assay. Figure S3: DNA methylation pattern in normal lymph node and TE methylation status in MCC specimens. Figure S4: DNA methylation in PD-1 and PDL-1. [file 13073_2021_946_MOESM1_ESM.pdf]

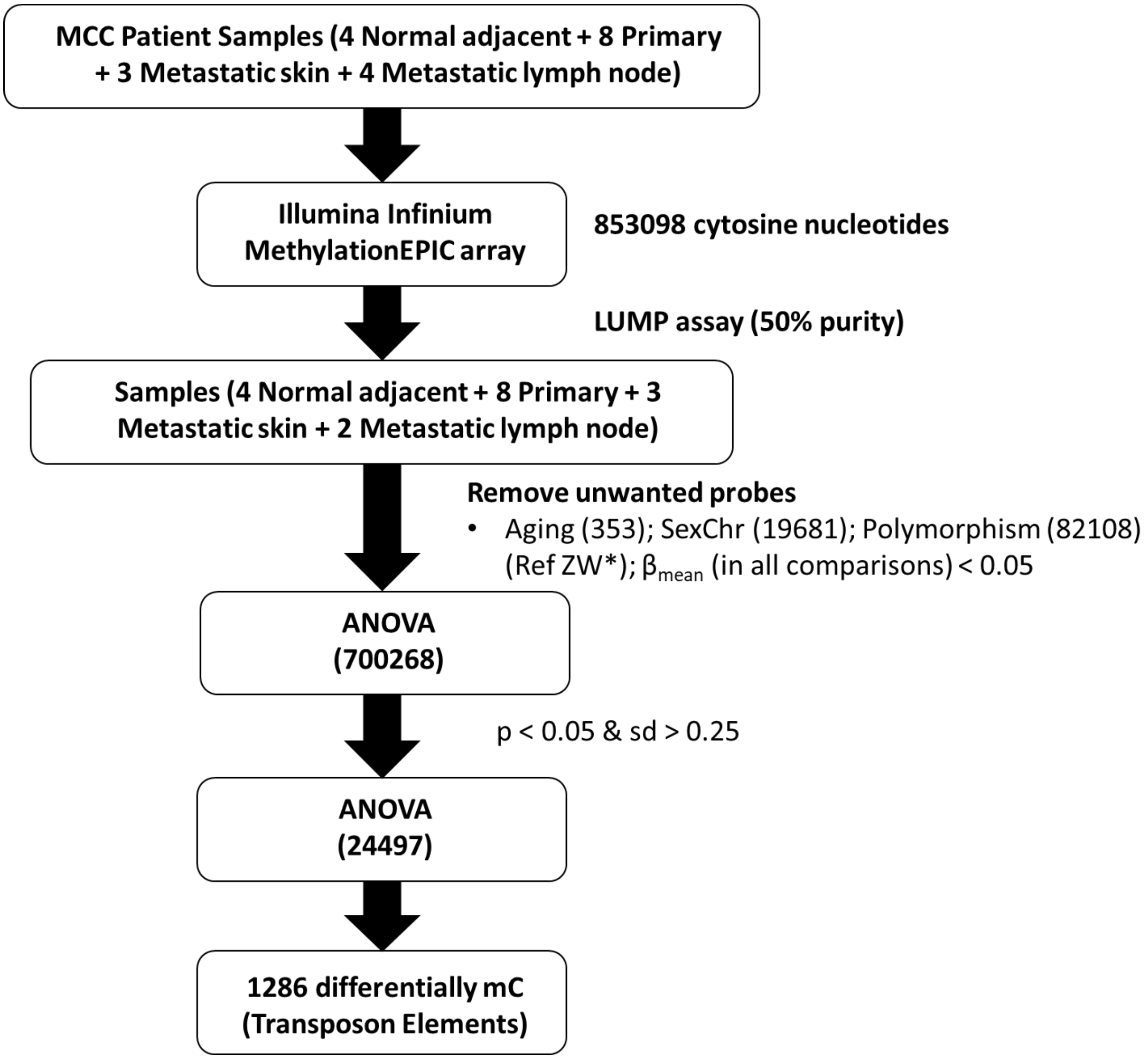

Figure S1: Outline for procedure and results obtained in this study.

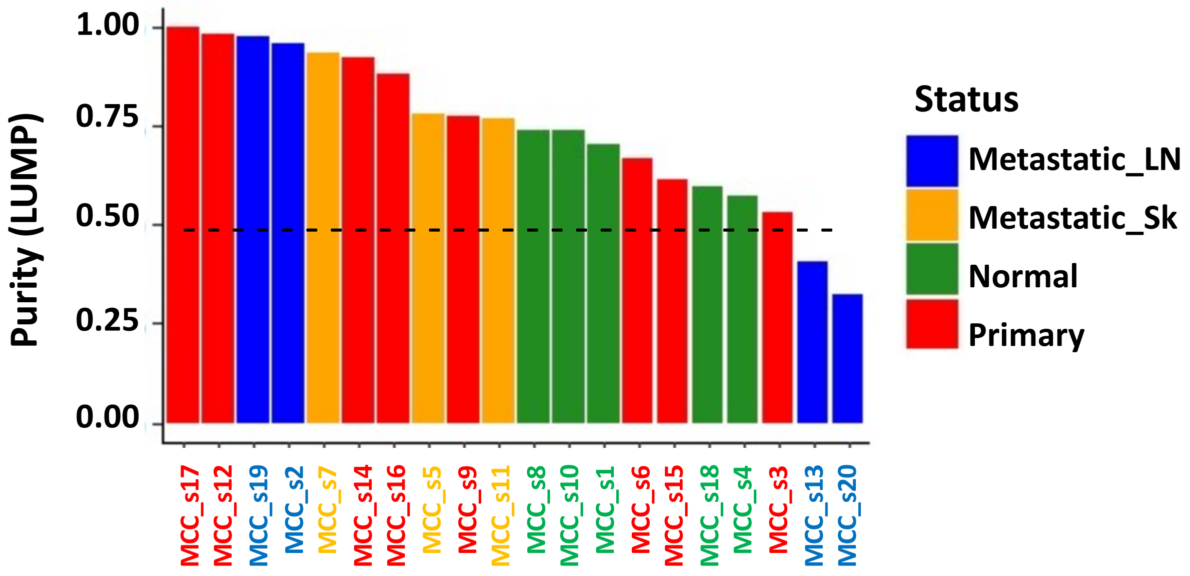

Figure S2: LUMP assay. Tumor purity for samples were assayed using the LUMP assay. Using a cut-off of 50%, two samples were removed from further analysis.

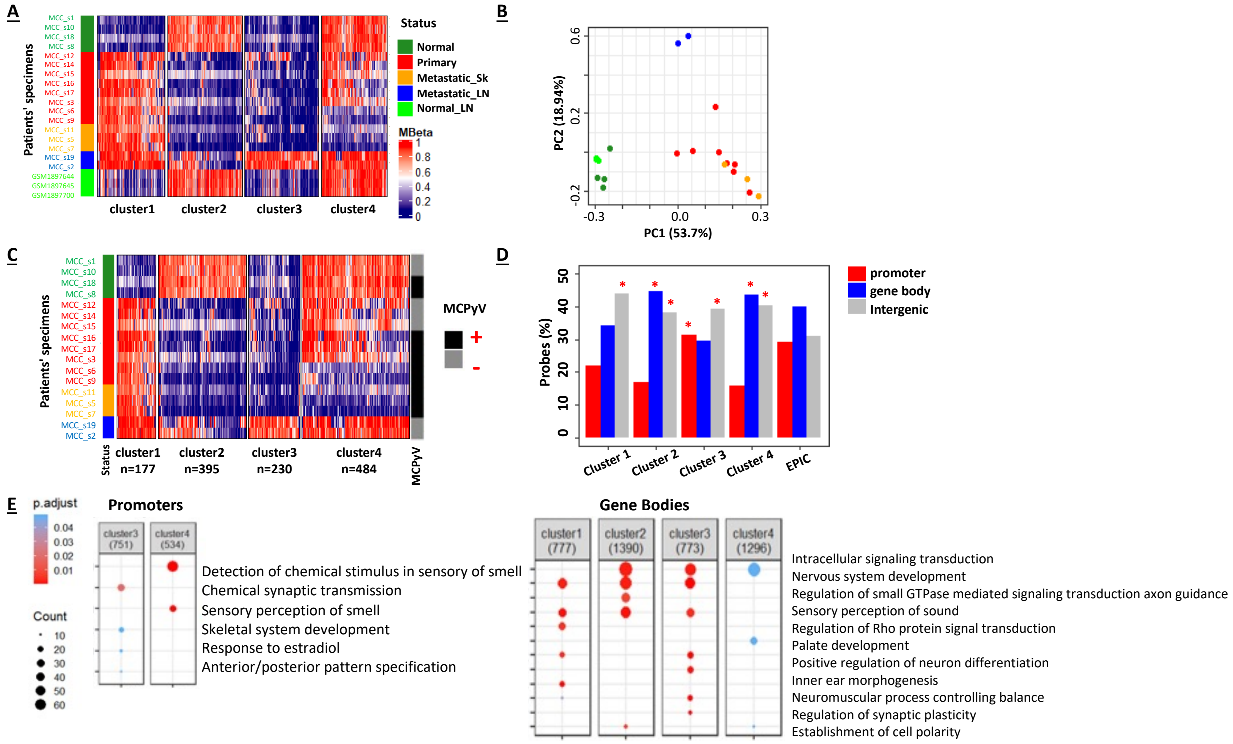

Figure S3: (A) MCC-specific DNA methylation (Figure 1A) was compared with normal lymph node DNA methyltion. DNA methylation in normal lymph nodes was similar to normal skin samples as shown in the heatmap. (B) PCA plot shows normal skin and normal lymph nodes grouped together. (C) Repetitive element DNA methylation was similar to that observed in all probes across Clusters 1-4 (Figure 1A). (D) MCC-specific DNA methylation (Figure 1A) located in different genic features including promoter, gene body and intergenic regions. The enrichment of probes in various genic features was reported using the phyper test. (*p < 0.05). (E) Differentially methylated genes either by promoter or gene body DNA methylation were annotated using GO terms for biological processes. GO analysis for biological terms using the R function *RDAVID* was performed and GO terms were reported.

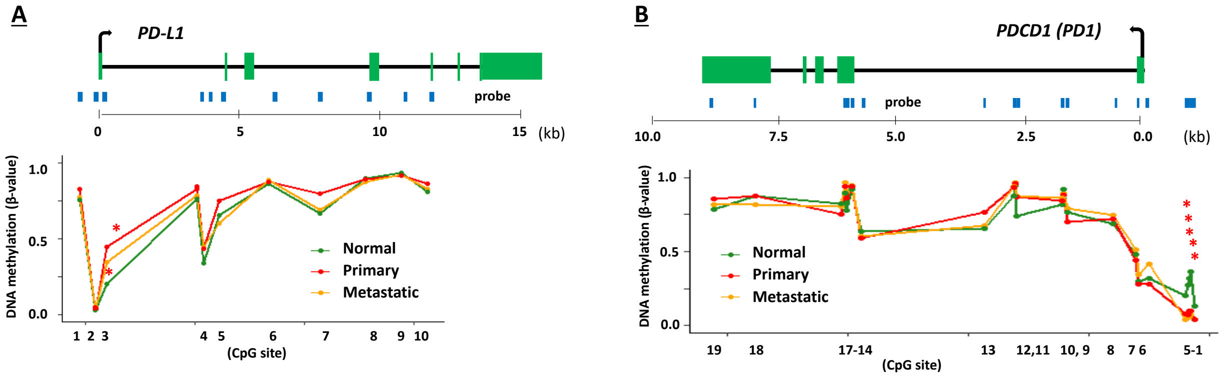

Figure S4: DNA methylation in *PD-1* and *PDL-1*. (A) and (B) Plots displaying all probes for *PDL-1* and *PD-1*, arranged in the genomic context, with promoter DNA hypomethylation or hypermethylation by comparing DNA methylation status in normal, primary, and metastatic (skin and lymph node) MCC samples. Adjusted p-value (FDR), $p < 0.05$ (*).
