# Supplementary material for: Characterizing DNA methylation signatures and their potential functional roles in Merkel cell carcinoma
Source: Genome Med. 2021 Aug 16;13:130. doi: 10.1186/s13073-021-00946-3 (PMC8365948; doi:10.1186/s13073-021-00946-3)
Supplement: Supplementary file 2 — Additional file 2. Supplemental Table 1: Top 10 differentially methylated probes based on MCPyV and DNA methylation status in four groups. Supplemental Table 2: List of genes regulated by DNA methylation in the MS1 cell line. Supplemental Table 3: List of genes regulated by DNA methylation in the MCC13 cell line. Supplemental Table 4: List of genes regulated by DNA methylation related to MCPyV status. [file 13073_2021_946_MOESM2_ESM.pdf]

Supplemental Table 1: Top 10 probes differentially methylated in MCPyV samples in four groups

Group 1: Hypermethylated in MCPyV (+)

	IlmnID	Location	CpG Island	hgnc_symbol	Proaohancer	MCPyV_P_Mean	MCPyV_N_Mean	Normal_Mean	p_adj
1	cg16574134	Body	N_Shore	PAX2	other	0.92	0.09	0.09	0.00
2	cg23206032	Body	Island	PAX2	other	0.79	0.12	0.11	0.01
3	cg04865531	Body	Island	SHANK3	other	0.80	0.13	0.20	0.01
4	cg13213810	Body	Island	SHANK3	other	0.83	0.17	0.20	0.00
5	cg27557378	Promoter	Island	DNASE1L2	Proaohancer	0.63	0.07	0.02	0.04
6	cg25731261	Promoter	S_Shore	BBC3	Proaohancer	0.75	0.20	0.37	0.03
7	cg21762523	Body	Island	NR2E1	other	0.61	0.07	0.03	0.03

Group 2: Hypomethylated in MCPyV (+)

	IlmnID	Location	CpG Island	hgnc_symbol	Proaohancer	MCPyV_P_Mean	MCPyV_N_Mean	Normal_Mean	p_adj
1	cg04586126	Body	Island	MXD3	other	0.10	0.96	0.63	0.00
2	cg05346688	Body	Island	SLCO3A1	other	0.09	0.95	0.90	0.00
3	cg23848678	other	OpenSea	Intergenic	other	0.16	0.95	0.87	0.00
4	cg24850121	Body	Island	SRGAP3	Proaohancer	0.09	0.87	0.52	0.02
5	cg07011961	Promoter	OpenSea	PRDM9	Proaohancer	0.15	0.92	0.56	0.01
6	cg21835874	Body	OpenSea	SMG6	other	0.07	0.85	0.49	0.03
7	cg09166973	Promoter	OpenSea	PRDM9	Proaohancer	0.19	0.96	0.70	0.01
8	cg12500731	other	N_Shelf	Intergenic	other	0.17	0.93	0.89	0.00
9	cg26845390	other	N_Shelf	Intergenic	other	0.16	0.93	0.89	0.00
10	cg01505148	Body	Island	U2AF2	other	0.18	0.94	0.89	0.00

Group 3: Hypomethylated in MCPyV (-)

	IlmnID	Location	CpG Island	hgnc_symbol	Proaohancer	MCPyV_P_Mean	MCPyV_N_Mean	Normal_Mean	p_adj
1	cg15555527	Body	Island	ANTXR2	Proaohancer	0.05	0.91	0.20	0.00
2	cg06445348	Body	Island	ILDR2	other	0.09	0.89	0.17	0.00

3	cg02091185	Promoter	N_Shore	RANBP17	Proaohancer	0.11	0.90	0.30	0.00
4	cg22718636	Body	Island	DGKQ	Proaohancer	0.14	0.93	0.43	0.00
5	cg07178969	other	OpenSea	Intergenic	other	0.06	0.85	0.33	0.03
6	cg08801479	Body	S_Shore	NOTCH4	other	0.17	0.95	0.46	0.00
7	cg12420900	Both	Island	TACC2	Proaohancer	0.09	0.87	0.36	0.00
8	cg04610534	Promoter	S_Shelf	CPEB3	Proaohancer	0.11	0.87	0.22	0.00
9	cg02444433	Promoter	OpenSea	PRDM9	Proaohancer	0.14	0.91	0.41	0.00
10	cg03936449	other	Island	Intergenic	other	0.08	0.84	0.30	0.04

Group 4: Hypomethylated in MCPyV (-)

	IlmnID	Location	CpG Island	hgnc_symbol	Proaohancer	MCPyV_P_Mean	MCPyV_N_Mean	Normal_Mean	p_adj
1	cg24046935	other	OpenSea	Intergenic	other	0.74	0.13	0.59	0.00
2	cg16949584	other	N_Shelf	Intergenic	other	0.78	0.18	0.69	0.03
3	cg14031975	other	OpenSea	Intergenic	other	0.69	0.12	0.48	0.01
4	cg07329929	Both	OpenSea	SLC22A8	other	0.69	0.16	0.43	0.05

Supplemental Table 2: List of genes regulated by DNA methylation in MS1

Group I

	hgnc_symbol	IlmnID	Location	CpG Island	Proaohancer	Cluster	MS1vsNSk_LogFC	MS1_meth
1	ACLY	cg14583225	Promoter	N_Shore	Proaohancer	cluster2	2.745512263	-0.597567954
2	ADRA2B	cg02599338	Promoter	N_Shore	Proaohancer	cluster2	4.967927815	-0.768441483
3	AFAP1	cg12728606	Promoter	OpenSea	Proaohancer	cluster2	3.175909428	-0.810910617
4	ANKRD33	cg19974223	Promoter	OpenSea	Proaohancer	cluster2	5.031376025	-0.650066779
5	ANKRD34B	cg26305827	Promoter	S_Shore	Proaohancer	cluster2	3.281229193	-0.748916117
6	ANKRD65	cg23537515	Promoter	S_Shore	Proaohancer	cluster2	3.974913576	-0.68874997
7	ARC	cg08387463	Promoter	Island	Proaohancer	cluster2	5.46274657	-0.775770166
8	ASPHD1	cg02488299	Promoter	S_Shore	Proaohancer	cluster2	3.534484216	-0.766309305
9	ATF5	cg23878206	Promoter	N_Shore	Proaohancer	cluster2	4.092665967	-0.624891323
10	ATP2B2	cg14496660	Promoter	OpenSea	Proaohancer	cluster2	3.354949138	-0.742752563
11	ATP6V0D2	cg06689659	Promoter	OpenSea	Proaohancer	cluster4	2.444042322	-0.811692681
12	BEST3	cg17277939	Promoter	OpenSea	Proaohancer	cluster2	6.173639252	-0.63111639
13	BOLA3	cg26529864	Promoter	S_Shore	Proaohancer	cluster4	2.744971639	-0.820608114
14	BTG2	cg20138067	Promoter	N_Shore	Proaohancer	cluster2	2.140448176	-0.317207726
15	C10orf82	cg13707002	Promoter	S_Shore	Proaohancer	cluster2	3.569150834	-0.737780962
16	C19orf81	cg14844855	Promoter	OpenSea	Proaohancer	cluster4	2.497245903	-0.757912518
17	C3orf67	cg00891146	Promoter	N_Shore	Proaohancer	cluster2	2.058219597	-0.534110714
18	CADM1	cg08066991	Promoter	S_Shore	Proaohancer	cluster2	2.696370971	-0.420358218
19	CALN1	cg14955982	Promoter	OpenSea	Proaohancer	cluster2	2.045177214	-0.622909623
20	CCER2	cg27096892	Promoter	OpenSea	Proaohancer	cluster2	6.737158779	-0.518837901
21	CDK2AP1	cg07729842	Promoter	S_Shore	Proaohancer	cluster4	2.50316203	-0.644759619
22	CDK5R2	cg23452259	Promoter	N_Shore	Proaohancer	cluster2	12.06738169	-0.356900583
23	CENPN	cg03284308	Promoter	N_Shore	Proaohancer	cluster2	3.636353543	-0.43725917
24	CENPW	cg11677992	Promoter	N_Shore	Proaohancer	cluster4	3.816509669	-0.397208904
25	CHGA	cg01901466	Promoter	N_Shore	Proaohancer	cluster4	9.069495534	-0.654252823

26	CHRNA1	cg09159103	Promoter	OpenSea	Proaohancer	cluster2	6.620008052	-0.561018438
27	CHST10	cg04874329	Promoter	N_Shore	Proaohancer	cluster4	2.775664051	-0.512583344
28	COA4	cg16820724	Promoter	N_Shore	Proaohancer	cluster2	2.145255866	-0.613606729
29	CXCL8	cg22834109	Promoter	OpenSea	Proaohancer	cluster4	7.574154959	-0.677731833
30	CYFIP2	cg01841762	Promoter	OpenSea	Proaohancer	cluster2	2.492110562	-0.456235579
31	DDN	cg03008550	Promoter	S_Shore	Proaohancer	cluster2	6.443600046	-0.590200749
32	DHX9	cg16243197	Promoter	N_Shore	Proaohancer	cluster2	2.304436973	-0.544924424
33	DNMT3A	cg10525105	Promoter	OpenSea	Proaohancer	cluster2	2.241555986	-0.47943714
34	DTYMK	cg00969323	Promoter	S_Shore	Proaohancer	cluster2	2.607518204	-0.659850748
35	DUSP26	cg14890736	Promoter	N_Shore	Proaohancer	cluster2	8.548941168	-0.573782326
36	ELFN1	cg23749162	Promoter	OpenSea	Proaohancer	cluster2	4.35516167	-0.568207178
37	EVA1A	cg12475422	Promoter	OpenSea	Proaohancer	cluster4	3.851104614	-0.736060221
38	EYA2	cg06209200	Promoter	OpenSea	Proaohancer	cluster2	4.701368092	-0.781970331
39	EZH2	cg17771588	Promoter	OpenSea	Proaohancer	cluster2	2.38502062	-0.622181196
40	FAM126A	cg00031708	Promoter	N_Shore	Proaohancer	cluster2	2.08822588	-0.837885844
41	FHL2	cg02231204	Promoter	S_Shore	Proaohancer	cluster2	2.75731217	-0.476545139
42	FKRP	cg05161243	Promoter	N_Shore	Proaohancer	cluster2	2.159216311	-0.593609721
43	FNDC5	cg20951444	Promoter	OpenSea	Proaohancer	cluster2	3.96096329	-0.630197408
44	FOXJ1	cg16861241	Promoter	S_Shore	Proaohancer	cluster2	4.29977663	-0.568941745
45	FSD1L	cg05677901	Promoter	N_Shore	Proaohancer	cluster2	2.439874313	-0.821995648
46	GAL3ST1	cg08215954	Promoter	OpenSea	Proaohancer	cluster2	3.069821761	-0.688606767
47	GAL3ST3	cg06569300	Promoter	S_Shore	Proaohancer	cluster4	3.719636963	-0.304700381
48	GFI1	cg24517501	Promoter	S_Shore	Proaohancer	cluster2	6.636662056	-0.581815473
49	GMNN	cg04570316	Promoter	S_Shore	Proaohancer	cluster2	2.387704068	-0.752231521
50	GNB1	cg27021587	Promoter	OpenSea	Proaohancer	cluster2	2.46041604	-0.53291056
51	GNG4	cg14011070	Promoter	N_Shore	Proaohancer	cluster2	8.545986439	-0.691213313
52	GPR156	cg16533530	Promoter	OpenSea	Proaohancer	cluster2	5.188966247	-0.705225001
53	GREM1	cg17327198	Promoter	OpenSea	Proaohancer	cluster2	3.033922305	-0.75095719
54	GSG1L2	cg15355740	Promoter	OpenSea	Proaohancer	cluster2	3.868081018	-0.701442433

55	HABP2	cg18833140	Promoter	OpenSea	Proaohancer	cluster2	3.461869679	-0.730798888
56	HIST1H2BO	cg12629515	Promoter	N_Shore	Proaohancer	cluster2	4.492945206	-0.422749901
57	HIST1H3J	cg12629515	Promoter	N_Shore	Proaohancer	cluster2	4.465773739	-0.422749901
58	HMGA1	cg02654940	Promoter	S_Shore	Proaohancer	cluster2	3.768301916	-0.680534346
59	HOXA3	cg22772747	Promoter	S_Shore	Proaohancer	cluster2	2.70103247	-0.364581803
60	HPCAL1	cg00141688	Promoter	OpenSea	Proaohancer	cluster2	3.388265011	-0.606769952
61	HPDL	cg03270777	Promoter	S_Shore	Proaohancer	cluster2	2.993625925	-0.788411521
62	HTR5A	cg13892088	Promoter	S_Shore	Proaohancer	cluster4	4.752449041	-0.571069104
63	HTR5A-AS1	cg13892088	Promoter	S_Shore	Proaohancer	cluster4	4.05759841	-0.685010609
64	HYAL3	cg04415662	Promoter	N_Shelf	Proaohancer	cluster2	2.112792611	-0.766900436
65	KCNA3	cg13925011	Promoter	Island	Proaohancer	cluster2	2.361860291	-0.691295429
66	KCNAB3	cg15951188	Promoter	Island	Proaohancer	cluster4	2.060512445	-0.554568309
67	KCNB2	cg12464898	Promoter	S_Shore	Proaohancer	cluster2	7.26994949	-0.776486642
68	KCND2	cg01338148	Promoter	N_Shore	Proaohancer	cluster2	3.835656338	-0.854058534
69	KCNMB2	cg23735602	Promoter	OpenSea	Proaohancer	cluster2	3.606781819	-0.646169093
70	KDM6B	cg27320191	Promoter	N_Shore	Proaohancer	cluster4	2.036409329	-0.520462882
71	KIF3C	cg05504158	Promoter	N_Shore	Proaohancer	cluster4	3.257480208	-0.644564389
72	KLHL5	cg11441074	Promoter	OpenSea	Proaohancer	cluster4	2.950259166	-0.764188686
73	KRT19	cg08966188	Promoter	S_Shore	Proaohancer	cluster2	2.091636374	-0.703015773
74	KRT20	cg06082664	Promoter	OpenSea	Proaohancer	cluster2	6.501587026	-0.839861689
75	LBR	cg22945457	Promoter	N_Shelf	Proaohancer	cluster4	2.147074009	-0.472269445
76	LGALS3BP	cg11105610	Promoter	OpenSea	Proaohancer	cluster2	2.808082159	-0.397066543
77	LINC00514	cg24313631	Promoter	OpenSea	Proaohancer	cluster2	3.887273331	-0.589005913
78	LINC00867	cg21574250	Promoter	OpenSea	Proaohancer	cluster4	5.082986811	-0.66589833
79	LINC01356	cg00154430	Promoter	S_Shore	Proaohancer	cluster2	3.407108969	-0.687203109
80	LINC01518	cg01389954	Promoter	OpenSea	Proaohancer	cluster2	7.772665006	-0.654358084
81	LINGO1	cg24614755	Promoter	OpenSea	Proaohancer	cluster2	2.840860327	-0.708342122
82	LPGAT1	cg11389491	Promoter	S_Shore	Proaohancer	cluster2	2.091733791	-0.735478593
83	LRRC43	cg01402099	Promoter	OpenSea	Proaohancer	cluster2	3.735474216	-0.688857055

84	LRRC4C	cg03673292	Promoter	OpenSea		Proaohancer	cluster4	2.700127633	-0.451784182
85	LYPD6	cg02474731	Promoter	N_Shore		Proaohancer	cluster2	2.785613416	-0.625362011
86	LYSMD2	cg02781526	Promoter	S_Shore		Proaohancer	cluster4	2.294428196	-0.360194229
87	MAP2	cg19447336	Promoter	OpenSea		Proaohancer	cluster2	2.022776308	-0.550989579
88	MCM5	cg15009484	Promoter	N_Shore		Proaohancer	cluster2	2.439712226	-0.578836839
89	METTL11B	cg03001143	Promoter	OpenSea		Proaohancer	cluster2	2.96634035	-0.517644323
90	MICAL2	cg27286609	Promoter	OpenSea		Proaohancer	cluster2	2.053271314	-0.685375902
91	MKRN3	cg13415551	Promoter	OpenSea		Proaohancer	cluster2	3.562859919	-0.686739782
92	MMEL1	cg19633446	Promoter	S_Shelf		Proaohancer	cluster2	2.324525309	-0.658556335
93	MPPED1	cg09929856	Promoter	Island		Proaohancer	cluster2	3.41641041	-0.497401571
94	MRPL3	cg15810640	Promoter	S_Shore		Proaohancer	cluster4	2.280161977	-0.625105393
95	MSLNL	cg02485642	Promoter	Island		Proaohancer	cluster2	2.8058194	-0.66508899
96	MYT1	cg23856293	Promoter	OpenSea		Proaohancer	cluster2	9.792918518	-0.716962109
97	NEFM	cg14139227	Promoter	N_Shore		Proaohancer	cluster2	9.093499151	-0.578283179
98	NEUROD6	cg11554507	Promoter	OpenSea		Proaohancer	cluster2	8.372421975	-0.67718096
99	NFASC	cg06896551	Promoter	OpenSea		Proaohancer	cluster2	2.39333789	-0.534415644
100	NHLH1	cg18023842	Promoter	OpenSea		Proaohancer	cluster2	6.883899044	-0.691672112
101	NIPSNAP1	cg00939432	Promoter	S_Shore		Proaohancer	cluster2	2.742164395	-0.720108428
102	NLRP14	cg12985581	Promoter	S_Shore		Proaohancer	cluster2	3.058278199	-0.77557713
103	NMUR2	cg09364205	Promoter	OpenSea		Proaohancer	cluster4	2.824434992	-0.309695426
104	NT5DC2	cg01729726	Promoter	S_Shore		Proaohancer	cluster2	2.51664268	-0.669257464
105	NTN3	cg01337429	Promoter	Island		Proaohancer	cluster2	5.213521382	-0.826421948
106	NUP62	cg23878206	Promoter	N_Shore		Proaohancer	cluster2	2.925845538	-0.624891323
107	NYAP2	cg02395751	Promoter	OpenSea		Proaohancer	cluster2	8.134628289	-0.53700382
108	OCIAD2	cg10150421	Promoter	S_Shore		Proaohancer	cluster2	2.692998434	-0.647837194
109	ODC1	cg15075241	Promoter	N_Shore		Proaohancer	cluster2	4.02256293	-0.762435648
110	OXSR1	cg25234080	Promoter	N_Shore		Proaohancer	cluster2	2.081712464	-0.769817755
111	PACSIN1	cg04941418	Promoter	N_Shore		Proaohancer	cluster2	3.994065416	-0.550974767
112	PBK	cg17973773	Promoter	S_Shore		Proaohancer	cluster2	4.459733932	-0.571538535

113	PHF19	cg02388253	Promoter	S_Shore	Proaohancer	cluster2	2.604074625	-0.673533964
114	PKIB	cg11889875	Promoter	OpenSea	Proaohancer	cluster2	3.105388428	-0.76225558
115	PLEKHA6	cg21600897	Promoter	OpenSea	Proaohancer	cluster2	2.487625773	-0.704309945
116	PRAME	cg19292908	Promoter	N_Shore	Proaohancer	cluster4	9.823442983	-0.791229209
117	PRDM8	cg05452645	Promoter	N_Shore	Proaohancer	cluster2	2.42723457	-0.521944875
118	RAD21	cg23559636	Promoter	N_Shelf	Proaohancer	cluster2	2.380493742	-0.680683716
119	RASD2	cg17059564	Promoter	S_Shore	Proaohancer	cluster2	5.965927225	-0.64029606
120	REC8	cg18512948	Promoter	Island	Proaohancer	cluster2	3.526355054	-0.558446516
121	RFPL1S	cg06182770	Promoter	OpenSea	Proaohancer	cluster2	5.50044847	-0.421456264
122	RMI1	cg14327541	Promoter	S_Shore	Proaohancer	cluster2	2.618713098	-0.510756718
123	RNF183	cg08006727	Promoter	OpenSea	Proaohancer	cluster2	2.48055575	-0.651866891
124	SARDH	cg12655879	Promoter	OpenSea	Proaohancer	cluster2	3.261621862	-0.575958119
125	SBK1	cg05139728	Promoter	S_Shelf	Proaohancer	cluster2	6.298418961	-0.535364946
126	SBK2	cg02363969	Promoter	S_Shore	Proaohancer	cluster2	4.054456742	-0.356032867
127	SCGB2A1	cg06334737	Promoter	OpenSea	Proaohancer	cluster2	2.068925282	-0.717819085
128	SCN1A	cg14046558	Promoter	OpenSea	Proaohancer	cluster2	6.445984348	-0.665794387
129	SEL1L3	cg26075090	Promoter	Island	Proaohancer	cluster2	3.249884724	-0.544917487
130	SEPHS2	cg26697065	Promoter	N_Shore	Proaohancer	cluster2	2.523010591	-0.809648348
131	SERPINA6	cg06058015	Promoter	OpenSea	Proaohancer	cluster4	2.306845152	-0.447438554
132	SEZ6	cg17977273	Promoter	S_Shore	Proaohancer	cluster2	4.594056825	-0.759473427
133	SF3A2	cg00305624	Promoter	S_Shore	Proaohancer	cluster2	3.534938391	-0.671883287
134	SGTA	cg01227084	Promoter	Island	Proaohancer	cluster2	2.601001191	-0.438971869
135	SH3BP4	cg08858245	Promoter	S_Shelf	Proaohancer	cluster2	3.041632561	-0.494063231
136	SLC9A1	cg26515162	Promoter	N_Shore	Proaohancer	cluster2	2.025411738	-0.424486772
137	SMAD9	cg05977007	Promoter	OpenSea	Proaohancer	cluster2	2.179071899	-0.736349823
138	SMARCA4	cg22809078	Promoter	OpenSea	Proaohancer	cluster2	2.154994793	-0.675702272
139	SMARCC1	cg05235590	Promoter	S_Shore	Proaohancer	cluster4	2.213981524	-0.802069904
140	SNAP25	cg09910691	Promoter	S_Shore	Proaohancer	cluster2	6.554856488	-0.586241833
141	SNAP25-AS1	cg09910691	Promoter	S_Shore	Proaohancer	cluster2	2.567970808	-0.586241833

142	SNRPB2	cg25926513	Promoter	S_Shore	Proaohancer	cluster2	2.276301945	-0.638522886
143	SOGA3	cg03217906	Promoter	S_Shore	Proaohancer	cluster2	3.989798044	-0.592675323
144	SPOCK2	cg23341805	Promoter	S_Shore	Proaohancer	cluster2	3.20883779	-0.605547943
145	ST18	cg10750742	Promoter	OpenSea	Proaohancer	cluster2	5.201877511	-0.462040545
146	STRA6	cg11787522	Promoter	OpenSea	Proaohancer	cluster2	3.807156766	-0.635373383
147	STRAP	cg14280171	Promoter	N_Shore	Proaohancer	cluster4	2.429108839	-0.586036991
148	SVOP	cg22733953	Promoter	OpenSea	Proaohancer	cluster2	5.658005081	-0.544956589
149	TBCB	cg04628320	Promoter	Island	Proaohancer	cluster2	2.383241919	-0.590224889
150	TCF3	cg08430680	Promoter	S_Shelf	Proaohancer	cluster2	3.276031357	-0.573920572
151	TFR2	cg05862812	Promoter	OpenSea	Proaohancer	cluster2	2.339375668	-0.728117986
152	TMEM108	cg23074996	Promoter	OpenSea	Proaohancer	cluster2	3.999313868	-0.640657265
153	TMEM169	cg03698716	Promoter	S_Shore	Proaohancer	cluster2	3.15428325	-0.714186152
154	TMEM200A	cg12236677	Promoter	OpenSea	Proaohancer	cluster4	2.604440073	-0.720571474
155	TMEM51	cg09069072	Promoter	S_Shore	Proaohancer	cluster2	2.08693522	-0.647254833
156	TMEM74	cg04414767	Promoter	N_Shelf	Proaohancer	cluster2	4.930328007	-0.670951435
157	TNFRSF11B	cg08920745	Promoter	S_Shore	Proaohancer	cluster4	2.25792976	-0.708268434
158	TP53	cg07760161	Promoter	N_Shore	Proaohancer	cluster2	2.260435177	-0.728138604
159	TPD52	cg09619309	Promoter	S_Shore	Proaohancer	cluster2	2.0011116	-0.603519594
160	TRIML2	cg16856980	Promoter	N_Shore	Proaohancer	cluster4	3.245779935	-0.495188486
161	TUBA1C	cg15309667	Promoter	OpenSea	Proaohancer	cluster2	2.086875146	-0.695949858
162	TUBB	cg27505349	Promoter	N_Shore	Proaohancer	cluster2	3.282381705	-0.411626376
163	UBE2I	cg26827394	Promoter	N_Shore	Proaohancer	cluster4	2.200190199	-0.540333391
164	UCP2	cg05638011	Promoter	N_Shelf	Proaohancer	cluster2	4.164550655	-0.69335585
165	USH2A	cg15590780	Promoter	OpenSea	Proaohancer	cluster2	7.063037414	-0.647105036
166	UTS2R	cg12598178	Promoter	Island	Proaohancer	cluster4	3.386303067	-0.345621656
167	VRK1	cg12239717	Promoter	OpenSea	Proaohancer	cluster4	2.208640342	-0.315600877
168	YJEFN3	cg22782873	Promoter	Island	Proaohancer	cluster2	2.207962433	-0.582185465
169	ZNF775	cg00417714	Promoter	OpenSea	Proaohancer	cluster2	2.659635746	-0.683675928
170	ZNF777	cg23871318	Promoter	S_Shore	Proaohancer	cluster2	2.271848328	-0.746277388

Group II

S.No.	hgnc_symbol	IlmnID	Refgrp	Relation_to_UCSC_CpG_Island	Proaohancer	Cluster	MS1vsNSk_LogFC	MS1_meth
1	MARCH4	cg00373538	Body	OpenSea	Proaohancer	cluster1	7.551660125	0.70349373
2	ACOT7	cg23780021	Body	OpenSea	other	cluster1	3.682231616	0.587924312
3	ACTN1	cg26499547	Body	OpenSea	Proaohancer	cluster1	2.816948401	0.533201914
4	ADAM19	cg13667243	Body	Island	Proaohancer	cluster1	3.918019521	0.41967263
5	ADAMTS6	cg21878650	Body	OpenSea	Proaohancer	cluster1	2.444639042	0.60711623
6	ANK1	cg15706250	Body	Island	other	cluster1	5.522807549	0.733611117
7	AP3B2	cg25584930	Body	OpenSea	other	cluster1	3.010262369	0.739255711
8	APBA2	cg09077934	Body	Island	other	cluster1	3.374022386	0.6581345
9	APOBEC3C	cg07186138	Body	OpenSea	other	cluster1	2.530667082	0.595796843
10	ASTN2	cg10555502	Body	OpenSea	other	cluster1	2.524398359	0.766918838
11	ATP8A2	cg23843044	Body	OpenSea	Proaohancer	cluster1	2.133498584	0.524443719
12	B4GALNT1	cg21361094	Body	Island	Proaohancer	cluster1	3.053629152	0.733633092
13	BARHL1	cg00668215	Body	Island	Proaohancer	cluster3	6.532942402	0.409693074
14	BARHL2	cg18337783	Body	N_Shore	other	cluster1	9.768315361	0.446741875
15	BRD4	cg22522598	Body	OpenSea	Proaohancer	cluster1	2.501261285	0.5800676
16	BRINP1	cg08453065	Body	OpenSea	other	cluster1	5.962974466	0.494875687
17	BRSK2	cg17429870	Body	N_Shore	Proaohancer	cluster1	3.494149954	0.619219506
18	BTBD17	cg12131414	Body	Island	other	cluster1	7.107711282	0.790086207
19	C1QL1	cg02395846	Body	Island	Proaohancer	cluster1	6.6913022	0.779053375
20	CA10	cg08663613	Body	OpenSea	other	cluster1	6.156846018	0.578581454
21	CACNA1A	cg14911505	Body	OpenSea	other	cluster1	6.061144588	0.649201094
22	CACNA1I	cg17802949	Body	Island	other	cluster1	4.622213999	0.679156441
23	CACNA2D2	cg02855924	Body	Island	Proaohancer	cluster1	3.381159478	0.689187565
24	CADPS	cg27007166	Body	OpenSea	other	cluster3	2.12959833	0.415857305
25	CBFA2T3	cg27435133	Body	S_Shore	other	cluster3	3.907304359	0.682653547
26	CBLN1	cg04438525	Body	N_Shore	other	cluster1	2.457015488	0.6390147

27	CCDC178	cg07027528	Body	OpenSea	other	cluster1	4.509471347	0.599972051
28	CCDC33	cg25590826	Body	OpenSea	other	cluster1	4.160370562	0.561776976
29	CCDC40	cg05159799	Body	S_Shelf	other	cluster1	2.615689093	0.592996106
30	CCNJL	cg23309856	Body	OpenSea	other	cluster1	2.784174534	0.54890861
31	CDH22	cg25484135	Body	N_Shore	other	cluster1	2.409916125	0.525939006
32	CDKN2A	cg14348664	Body	N_Shore	other	cluster1	8.109627208	0.724299554
33	CELF4	cg26790823	Body	OpenSea	Proaohancer	cluster1	4.409551207	0.525583313
34	CELSR3	cg02858118	Body	Island	Proaohancer	cluster1	3.814264352	0.810022107
35	CHRNA4	cg15567340	Body	S_Shore	other	cluster3	6.441543156	0.308800387
36	CHST9	cg07204861	Body	OpenSea	Proaohancer	cluster1	5.970747912	0.560131924
37	CIT	cg14132388	Body	S_Shore	other	cluster3	3.048775812	0.432458563
38	CMIP	cg07137626	Body	OpenSea	Proaohancer	cluster1	2.185894998	0.660968327
39	CNPY1	cg26388509	Body	N_Shelf	other	cluster1	3.191767075	0.600449048
40	CNTN5	cg00930832	Body	OpenSea	other	cluster3	2.155872954	0.491123405
41	CORO1C	cg23749353	Body	OpenSea	Proaohancer	cluster3	2.518960441	0.404457245
42	CPNE7	cg16616467	Body	Island	other	cluster1	4.625393068	0.546054051
43	CREB3L2	cg11787891	Body	OpenSea	other	cluster1	2.238952099	0.63623695
44	CRYBA2	cg17054386	Body	N_Shelf	other	cluster1	10.40864629	0.546297309
45	CSMD3	cg06230615	Body	OpenSea	other	cluster1	5.345973694	0.562016232
46	CTNND2	cg01937840	Body	OpenSea	Proaohancer	cluster3	4.090004282	0.574252912
47	CUEDC1	cg20961045	Body	OpenSea	Proaohancer	cluster3	2.368956621	0.547828571
48	DCDC2	cg22028698	Body	OpenSea	Proaohancer	cluster1	4.060832991	0.547555958
49	DIAPH3	cg24289237	Body	OpenSea	other	cluster1	3.965795715	0.391021978
50	DLGAP3	cg11951427	Body	S_Shore	other	cluster1	6.053848833	0.674987561
51	DLX1	cg17737681	Body	Island	Proaohancer	cluster3	4.580667501	0.628105371
52	DLX5	cg13462129	Body	Island	other	cluster1	3.020078443	0.660962457
53	DMRTA2	cg00945234	Body	Island	other	cluster3	5.885781048	0.772652112
54	DPP6	cg26414472	Body	OpenSea	other	cluster1	2.706663935	0.579161379
55	DPYSL5	cg23332900	Body	OpenSea	other	cluster3	12.21523087	0.48763053

56	EEF1A2	cg26186239	Body	Island	Proaohancer	cluster1	5.442335517	0.738273386
57	EN2	cg16935980	Body	Island	other	cluster1	6.702523914	0.556682549
58	EPB41L3	cg06459104	Body	OpenSea	Proaohancer	cluster1	2.784612803	0.599989703
59	EPHA10	cg24375409	Body	Island	other	cluster1	2.874174553	0.638921776
60	ERC2	cg23599951	Body	OpenSea	Proaohancer	cluster1	3.33494257	0.482548798
61	ETS1	cg23774988	Body	Island	other	cluster1	2.412968934	0.760183241
62	FAM107B	cg00134210	Body	N_Shore	other	cluster1	2.691732037	0.641134848
63	FAM171A1	cg02217247	Body	OpenSea	Proaohancer	cluster3	3.416524592	0.377990393
64	FAM57B	cg06985993	Body	N_Shelf	other	cluster3	6.441644339	0.481296563
65	FGF5	cg14349667	Body	S_Shore	other	cluster1	6.649292843	0.805386356
66	FOXA2	cg20504791	Body	N_Shore	other	cluster1	11.6248805	0.428685535
67	FOXK2	cg21265419	Body	OpenSea	other	cluster1	2.106532761	0.360346974
68	GABRG3	cg23916360	Body	OpenSea	other	cluster3	3.252970429	0.355250477
69	GALNTL6	cg06814616	Body	OpenSea	Proaohancer	cluster1	3.544685708	0.774746248
70	GDF6	cg05690644	Body	Island	Proaohancer	cluster1	6.545713211	0.66103318
71	GNA12	cg16569650	Body	N_Shore	other	cluster1	2.742827804	0.685164399
72	GNAO1	cg10504751	Body	OpenSea	Proaohancer	cluster1	3.729595503	0.619197685
73	GPR158	cg02234281	Body	OpenSea	Proaohancer	cluster1	3.943152091	0.583090912
74	GPRIN1	cg02098752	Body	Island	other	cluster1	5.79170361	0.307858482
75	GRAMD1A	cg25450266	Body	S_Shelf	other	cluster3	2.006535796	0.44652122
76	GRIK2	cg22851944	Body	S_Shore	other	cluster1	2.113026923	0.659127262
77	GRIK3	cg20779373	Body	OpenSea	other	cluster1	4.800991799	0.325384937
78	GRIN2D	cg23444265	Body	Island	Proaohancer	cluster1	5.167989006	0.782661074
79	GRM4	cg00070899	Body	S_Shore	other	cluster1	8.398834767	0.517218104
80	GRM8	cg08696071	Body	OpenSea	Proaohancer	cluster1	7.561385966	0.368618868
81	HES3	cg03327263	Body	N_Shore	other	cluster1	4.069241311	0.410983608
82	HMX2	cg22886575	Body	Island	other	cluster1	5.901275014	0.774677541
83	HOXC11	cg08857479	Body	S_Shore	other	cluster1	2.186868833	0.663579515
84	HSF2BP	cg23043899	Body	OpenSea	Proaohancer	cluster1	2.464440489	0.681364784

85	IGSF21	cg07371981	Body	OpenSea	other	cluster1	3.036423954	0.633745608
86	INHBA	cg08786829	Body	OpenSea	other	cluster3	3.515062034	0.352887161
87	INSRR	cg16438688	Body	Island	other	cluster1	3.362571946	0.665276203
88	INTS7	cg09504092	Body	OpenSea	other	cluster3	2.072341535	0.414167886
89	ISL2	cg08307030	Body	Island	other	cluster3	6.514678624	0.654241263
90	ITPKA	cg22888055	Body	S_Shore	other	cluster1	2.894856773	0.676567469
91	KCNC2	cg06563089	Body	Island	other	cluster1	3.290798505	0.741306182
92	KCNH2	cg16817992	Body	Island	other	cluster1	5.849410457	0.764082922
93	KCNH3	cg08876130	Body	OpenSea	other	cluster1	2.975702244	0.457996695
94	KCNK3	cg27485108	Body	Island	other	cluster1	4.499672997	0.837953793
95	KCNQ2	cg07904817	Body	N_Shore	other	cluster1	9.980389247	0.644483981
96	KHDRBS3	cg00753112	Body	OpenSea	Proaohancer	cluster1	2.310489696	0.708301094
97	KIF19	cg18259003	Body	Island	other	cluster1	7.164810958	0.836040303
98	KIF26A	cg12924430	Body	Island	other	cluster1	2.717284037	0.669741147
99	KIRREL2	cg01588826	Body	S_Shore	Proaohancer	cluster1	8.659197533	0.745463172
100	KIRREL3	cg01775559	Body	OpenSea	other	cluster1	3.32031984	0.672438763
101	KSR2	cg05738715	Body	OpenSea	Proaohancer	cluster1	5.117882329	0.65767354
102	LASP1	cg15883678	Body	OpenSea	other	cluster3	2.508155311	0.570340335
103	LDB3	cg11911560	Body	OpenSea	Proaohancer	cluster3	2.675588928	0.374338325
104	LDLR	cg22006489	Body	OpenSea	Proaohancer	cluster1	2.04257596	0.6944535
105	LGALS1	cg09023892	Body	N_Shore	Proaohancer	cluster1	2.062357482	0.679589731
106	LHFPL3	cg03393137	Body	OpenSea	other	cluster1	3.911694205	0.657241453
107	LHX2	cg14425564	Body	OpenSea	other	cluster1	2.294724235	0.588259216
108	LHX9	cg22443762	Body	Island	other	cluster1	9.493125199	0.61250893
109	LINC00870	cg10162076	Body	OpenSea	other	cluster1	2.161780613	0.50518971
110	LINC01122	cg23641231	Body	OpenSea	other	cluster1	2.869705135	0.697523763
111	LINC01399	cg27370618	Body	OpenSea	other	cluster1	6.227734561	0.465273785
112	LINC01529	cg11791444	Body	OpenSea	other	cluster1	2.508283173	0.687948691
113	LMX1B	cg14225031	Body	Island	Proaohancer	cluster1	2.392892771	0.701186122

114	LRTM2	cg18952026	Body	OpenSea	Proaohancer	cluster1	6.092105405	0.766831003
115	MAPK8IP2	cg09877744	Body	Island	other	cluster1	3.595914866	0.815128907
116	MAST1	cg06537894	Body	Island	other	cluster1	3.729316978	0.670136626
117	MB	cg25157817	Body	OpenSea	other	cluster1	2.024686951	0.53935159
118	MNX1	cg02440420	Body	Island	other	cluster1	7.800586842	0.452011881
119	MTUS2	cg11108505	Body	OpenSea	other	cluster3	4.015050399	0.492868461
120	MYO15A	cg02458237	Body	OpenSea	other	cluster1	4.837314461	0.492101099
121	NCAM1	cg09843482	Body	OpenSea	other	cluster3	3.834958003	0.45968262
122	NCOR2	cg00765705	Body	OpenSea	other	cluster1	2.250885714	0.432663336
123	NELL1	cg20916120	Body	OpenSea	Proaohancer	cluster3	4.528628407	0.417711418
124	NEUROD1	cg11528849	Body	OpenSea	other	cluster1	11.47870336	0.817142347
125	NEUROD2	cg20153196	Body	N_Shore	other	cluster3	5.566887418	0.375931228
126	NFIC	cg15658793	Body	Island	other	cluster3	2.070092942	0.556579098
127	NKX2-1	cg13980454	Body	N_Shore	other	cluster1	7.587857331	0.566115921
128	NKX6-1	cg25830182	Body	N_Shore	other	cluster1	9.508400919	0.696343861
129	NPHS1	cg20655980	Body	OpenSea	other	cluster1	8.536725798	0.823253556
130	NREP	cg22680692	Body	OpenSea	other	cluster3	2.019649606	0.453251932
131	NRGN	cg10938436	Body	Island	Proaohancer	cluster1	3.978881073	0.783123035
132	NRXN2	cg10940462	Body	Island	Proaohancer	cluster1	3.646965226	0.639247968
133	NRXN3	cg12503756	Body	OpenSea	other	cluster1	3.308627612	0.626522838
134	NTRK1	cg01357370	Body	Island	other	cluster1	2.746744542	0.665276203
135	NXPH1	cg13578408	Body	S_Shore	Proaohancer	cluster1	4.331081091	0.733032437
136	NXPH4	cg04850731	Body	Island	other	cluster1	4.597284439	0.611042656
137	ONECUT2	cg15276629	Body	OpenSea	other	cluster1	8.389221076	0.706317789
138	OTP	cg14446615	Body	N_Shore	other	cluster1	5.538649165	0.70607571
139	PAX2	cg17589590	Body	S_Shore	other	cluster1	11.57556288	0.741841053
140	PAX5	cg20815683	Body	Island	other	cluster1	9.461190912	0.744285308
141	PAX6	cg16616521	Body	Island	other	cluster1	3.716006358	0.68036786
142	PCBP3	cg08455194	Body	OpenSea	Proaohancer	cluster3	3.404443401	0.301705131

143	PCDH9	cg10707081	Body	OpenSea		Proaohancer	cluster1	3.058212529	0.468507097
144	PCSK2	cg23756474	Body	N_Shore		other	cluster1	2.533046324	0.640572682
145	PDE1C	cg05512100	Body	N_Shore		other	cluster3	4.024748587	0.474100029
146	PFKP	cg20520804	Body	S_Shore		other	cluster1	2.751660769	0.540279316
147	PHOX2B	cg10192893	Body	Island		Proaohancer	cluster1	5.113930891	0.727732847
148	PLCXD2	cg10335947	Body	OpenSea		other	cluster1	2.786939673	0.577236974
149	POT1-AS1	cg17105921	Body	OpenSea		other	cluster3	2.324059606	0.5579229
150	PRDM13	cg17989257	Body	N_Shore		other	cluster1	6.426928316	0.715795281
151	PRKAR1B	cg07454365	Body	N_Shelf		other	cluster1	3.10188892	0.490159706
152	PRSS23	cg24069172	Body	OpenSea		other	cluster1	2.009830701	0.616410643
153	PTPRN2	cg05124021	Body	S_Shore		other	cluster1	3.749932196	0.518815895
154	PTPRR	cg22483686	Body	OpenSea		Proaohancer	cluster1	2.235692248	0.745121197
155	RAP1GAP	cg11531272	Body	OpenSea		other	cluster1	2.221474871	0.594974102
156	RASSF3	cg18544413	Body	OpenSea		Proaohancer	cluster1	2.12094564	0.631241459
157	RBFOX1	cg19636673	Body	OpenSea		other	cluster1	5.603195847	0.564501208
158	RGS7	cg04120815	Body	OpenSea		other	cluster1	3.986407841	0.751555435
159	RIMS2	cg14750367	Body	OpenSea		other	cluster1	6.346461244	0.475752506
160	ROBO3	cg01283096	Body	N_Shore		other	cluster3	2.114878752	0.631531674
161	RTN2	cg02899346	Body	N_Shore		other	cluster3	2.653680207	0.640678974
162	RUNDC3A	cg02115818	Body	S_Shore		other	cluster1	2.8391998	0.784580135
163	SACS	cg10526559	Body	S_Shelf		Proaohancer	cluster1	3.02237735	0.624296763
164	SAE1	cg26902742	Body	OpenSea		Proaohancer	cluster1	2.603719581	0.573514361
165	SATB2	cg20731529	Body	OpenSea		Proaohancer	cluster1	3.422252918	0.402991557
166	SCG5	cg08770263	Body	OpenSea		other	cluster1	4.606106169	0.654111962
167	SCHIP1	cg09694300	Body	OpenSea		Proaohancer	cluster1	2.168682441	0.402337572
168	SCN8A	cg21974358	Body	Island		Proaohancer	cluster1	4.052049093	0.674852804
169	SCNN1A	cg20621699	Body	Island		other	cluster3	2.094455124	0.437967964
170	SCRT2	cg05073843	Body	Island		other	cluster1	8.595411929	0.76493714
171	SDK1	cg17458659	Body	OpenSea		other	cluster1	2.030602093	0.757261202

172	SEZ6L2	cg06890619	Body	Island	Proaohancer	cluster1	5.731487155	0.765959968
173	SH2B2	cg07512361	Body	Island	other	cluster1	2.784305401	0.67230775
174	SHB	cg25036498	Body	OpenSea	Proaohancer	cluster1	2.156093644	0.634846123
175	SHISA7	cg21719704	Body	N_Shelf	other	cluster1	2.053138773	0.605011202
176	SIM2	cg27312652	Body	Island	Proaohancer	cluster3	4.872446644	0.674269207
177	SIX6	cg14507337	Body	Island	other	cluster3	6.129403529	0.484262454
178	SKOR1	cg21611830	Body	Island	Proaohancer	cluster1	3.671276454	0.633595728
179	SLC12A5	cg26729880	Body	OpenSea	other	cluster1	2.80703195	0.741477605
180	SLC17A6	cg09150064	Body	S_Shore	Proaohancer	cluster1	10.41102131	0.595390707
181	SLC17A7	cg00392377	Body	Island	other	cluster3	2.367535328	0.645663514
182	SLC24A2	cg23378460	Body	OpenSea	other	cluster1	2.582462965	0.697483359
183	SLC32A1	cg11519217	Body	Island	Proaohancer	cluster1	6.229017141	0.604413341
184	SLC4A4	cg11363527	Body	OpenSea	Proaohancer	cluster1	4.624961994	0.591619927
185	SLC6A18	cg11681428	Body	Island	Proaohancer	cluster1	3.958050434	0.667263394
186	SLC6A3	cg21163347	Body	OpenSea	other	cluster3	8.32802175	0.367906383
187	SLC6A5	cg15083015	Body	S_Shelf	other	cluster1	4.491334228	0.64449891
188	SLC7A14	cg01977413	Body	OpenSea	Proaohancer	cluster1	4.117669615	0.573862466
189	SLC8A2	cg11794430	Body	OpenSea	other	cluster1	4.909203048	0.588804398
190	SMAD3	cg04171324	Body	OpenSea	other	cluster1	2.12967896	0.57681089
191	SMAD7	cg11909137	Body	OpenSea	Proaohancer	cluster1	2.634275208	0.791219055
192	SMTNL2	cg26089877	Body	Island	other	cluster1	2.402652703	0.634695244
193	SPEG	cg13437337	Body	Island	other	cluster1	2.030838905	0.925651734
194	SPTBN4	cg02576468	Body	Island	Proaohancer	cluster1	2.886540823	0.590072267
195	SRGAP1	cg04627470	Body	OpenSea	Proaohancer	cluster1	2.36279069	0.645466417
196	SRRM3	cg04115680	Body	Island	other	cluster1	3.955975116	0.784504856
197	SRRM4	cg01254303	Body	Island	other	cluster1	7.170107475	0.702620478
198	SSBP3	cg10614054	Body	OpenSea	other	cluster1	2.420515171	0.550458252
199	ST6GALNAC5	cg17405164	Body	OpenSea	Proaohancer	cluster1	4.201579345	0.629311475
200	STK32B	cg27153759	Body	Island	other	cluster3	2.645926845	0.47254997

201	SYN2	cg27396259	Body	OpenSea	other	cluster3	2.614529282	0.757708284
202	SYNJ2	cg04786164	Body	OpenSea	Proaohancer	cluster1	2.20518691	0.583751874
203	SYNPR	cg11238995	Body	OpenSea	Proaohancer	cluster3	3.939955163	0.684279612
204	SYT1	cg24588941	Body	OpenSea	other	cluster1	4.924127878	0.489280293
205	SYT16	cg01491926	Body	OpenSea	other	cluster1	6.270755223	0.587378918
206	SYT7	cg12189835	Body	N_Shore	other	cluster1	3.764817231	0.748877412
207	SZRD1	cg05247850	Body	OpenSea	other	cluster1	2.148288094	0.572107987
208	TARID	cg16789867	Body	OpenSea	other	cluster1	2.567778033	0.561788918
209	TBR1	cg12520549	Body	S_Shore	Proaohancer	cluster1	2.156521052	0.660085614
210	TBX20	cg07555268	Body	N_Shelf	other	cluster1	7.945357701	0.599916623
211	TESC	cg06799805	Body	S_Shore	Proaohancer	cluster1	2.390711838	0.599759868
212	TFAP2A	cg08178786	Body	S_Shore	other	cluster3	2.39909265	0.301178807
213	TFAP2B	cg23015341	Body	Island	other	cluster3	2.585506521	0.656773113
214	TFAP2D	cg13654594	Body	S_Shore	Proaohancer	cluster1	9.320062975	0.591481651
215	TGFB2	cg07810039	Body	OpenSea	other	cluster1	3.918394957	0.752178208
216	TLX1	cg23340017	Body	N_Shore	other	cluster3	4.063279376	0.72527138
217	TMEM132E	cg02927904	Body	Island	other	cluster1	5.475289643	0.622280059
218	TMEM151B	cg03498886	Body	Island	other	cluster3	3.319836131	0.562760437
219	TMEM178B	cg14319628	Body	OpenSea	other	cluster3	5.27969703	0.303734556
220	TOP1	cg25135000	Body	OpenSea	other	cluster3	2.0242823	0.485342663
221	TPM4	cg06590173	Body	S_Shore	Proaohancer	cluster1	2.175860614	0.715497869
222	TRPM8	cg23993463	Body	Island	other	cluster1	4.451524004	0.591590837
223	UNC13A	cg02617655	Body	Island	Proaohancer	cluster3	7.290055451	0.910550715
224	UNC5A	cg19371429	Body	OpenSea	other	cluster3	4.736969975	0.361669099
225	UNC79	cg11085942	Body	OpenSea	Proaohancer	cluster1	6.505209137	0.696913507
226	UNC80	cg00285941	Body	OpenSea	other	cluster1	4.064752311	0.5703393
227	VAV2	cg08221952	Body	OpenSea	Proaohancer	cluster1	3.493306326	0.343854077
228	VAX1	cg26263263	Body	N_Shore	other	cluster1	3.666815837	0.646844867
229	VSTM2L	cg09969478	Body	OpenSea	other	cluster3	2.15248903	0.384500992

230	WNK4	cg18963509	Body	Island	other	cluster1	2.128829986	0.505763184
231	WT1	cg25782229	Body	N_Shore	other	cluster1	7.134187768	0.658566576
232	ZNF827	cg05267427	Body	OpenSea	Proaohancer	cluster1	2.57997843	0.538920115

Group III

S.No.	hgnc_symbol	IlmnID	Refgrp	Relation_to_UCSC_CpG_Island	Proaohancer	Cluster	MS1vsNSk_LogFC	MS1_meth
1	SEPT4	cg06976613	Body	OpenSea	other	cluster4	-3.217153231	-0.746920047
2	A2M	cg06699201	Body	OpenSea	other	cluster4	-9.370395885	-0.666075569
3	AADACL2-AS1	cg15453782	Body	OpenSea	other	cluster4	-8.046705738	-0.797014903
4	ABCA12	cg04530015	Body	OpenSea	other	cluster4	-8.381718771	-0.464660545
5	ABCA13	cg14674740	Body	OpenSea	other	cluster2	-2.576067258	-0.8086699
6	ABCA4	cg19196920	Body	OpenSea	other	cluster4	-5.392834099	-0.321015571
7	ABCC11	cg27185993	Body	OpenSea	other	cluster2	-3.34629633	-0.751405487
8	ABCC8	cg12401398	Body	OpenSea	Proaohancer	cluster2	-5.274863347	-0.491566363
9	ABI3	cg14009013	Body	N_Shore	other	cluster4	-5.01415646	-0.711120289
10	ABLIM1	cg27354918	Body	OpenSea	other	cluster2	-3.272115988	-0.577923171
11	ACAN	cg04886934	Body	OpenSea	other	cluster4	-4.461972448	-0.908901828
12	ACPP	cg25111496	Body	OpenSea	other	cluster2	-11.1901	-0.681808375
13	ACSM5	cg27514874	Body	OpenSea	other	cluster4	-7.465526039	-0.803480149
14	ADAMTS10	cg00420246	Body	N_Shore	other	cluster4	-2.074598399	-0.828824702
15	ADAMTS2	cg03259333	Body	N_Shore	other	cluster2	-6.57288676	-0.68993261
16	ADCY2	cg00247998	Body	OpenSea	other	cluster2	-8.394580522	-0.343874324
17	ADCY5	cg10379479	Body	OpenSea	Proaohancer	cluster2	-2.357081211	-0.693509093
18	ADGRD1	cg18923655	Body	OpenSea	other	cluster2	-6.279830754	-0.69358289
19	AGBL1	cg21205579	Body	OpenSea	other	cluster4	-3.921019132	-0.797346459
20	AKR1C3	cg08894837	Body	OpenSea	other	cluster4	-4.317666552	-0.627079286
21	AKR1C4	cg12357263	Body	OpenSea	other	cluster4	-5.039429466	-0.75544497

22	ALB	cg24018190	Body	OpenSea	Proaohancer	cluster4	-3.496894424	-0.406293193
23	ALDH8A1	cg18761400	Body	OpenSea	other	cluster2	-2.431230499	-0.661001537
24	ANK3	cg12655303	Body	OpenSea	Proaohancer	cluster2	-2.927050594	-0.760374126
25	ANKUB1	cg20321106	Body	OpenSea	other	cluster4	-2.778869591	-0.581299148
26	ANO1	cg13065497	Body	OpenSea	Proaohancer	cluster2	-10.38067745	-0.569990231
27	ANO2	cg25415853	Body	OpenSea	other	cluster2	-4.385700256	-0.693250523
28	ANTXRLP1	cg16800932	Body	OpenSea	other	cluster2	-6.619552241	-0.671165984
29	AOC1	cg08801691	Body	S_Shore	other	cluster4	-6.145697262	-0.381118917
30	ARHGAP15	cg27474094	Body	OpenSea	Proaohancer	cluster2	-5.024015766	-0.568908115
31	ARHGAP25	cg03293050	Body	OpenSea	Proaohancer	cluster2	-3.535475521	-0.590326454
32	ARHGEF10	cg14932939	Body	OpenSea	other	cluster2	-3.373674012	-0.646590697
33	ARHGEF3	cg15009130	Body	OpenSea	other	cluster2	-2.622835437	-0.486788798
34	ARHGEF3-AS1	cg12880229	Body	OpenSea	other	cluster4	-7.781306192	-0.392601895
35	ARRB1	cg07512639	Body	OpenSea	other	cluster2	-3.052050433	-0.511434489
36	ATP13A4	cg23997451	Body	OpenSea	other	cluster2	-8.190332928	-0.667834259
37	ATP2C2	cg26449717	Body	OpenSea	other	cluster2	-9.107241511	-0.754513981
38	BCAS1	cg02450219	Body	OpenSea	other	cluster2	-3.617274418	-0.521003521
39	BCO2	cg11741468	Body	OpenSea	other	cluster4	-2.687311401	-0.42496124
40	BPIFC	cg12406374	Body	OpenSea	other	cluster2	-8.639624023	-0.700980199
41	BTNL2	cg18880384	Body	OpenSea	other	cluster2	-2.823093646	-0.644205077
42	BTNL9	cg11886002	Body	S_Shore	other	cluster4	-6.306132778	-0.6765306
43	C22orf34	cg09640001	Body	OpenSea	other	cluster2	-6.222387649	-0.725294978
44	C3	cg19254532	Body	S_Shore	Proaohancer	cluster4	-5.409744039	-0.705781361
45	C3orf52	cg08451978	Body	OpenSea	Proaohancer	cluster2	-3.170465726	-0.694867846
46	C4orf36	cg22701096	Body	OpenSea	other	cluster4	-2.340123101	-0.569106836
47	C8orf37-AS1	cg13136972	Body	OpenSea	other	cluster4	-2.59466433	-0.599157211
48	C9	cg03453248	Body	OpenSea	other	cluster4	-6.808883672	-0.824929666
49	CA13	cg05071334	Body	OpenSea	other	cluster4	-3.157488348	-0.391297462
50	CACNA2D4	cg03882634	Body	OpenSea	other	cluster2	-2.179081006	-0.515000755

51	CACNB4	cg00695781	Body	OpenSea	other	cluster4	-6.262457856	-0.564132809
52	CADM3	cg21532811	Body	OpenSea	other	cluster2	-3.138335801	-0.588511118
53	CALCR	cg17918845	Body	OpenSea	other	cluster4	-3.284751691	-0.855775066
54	CATSPERB	cg18158970	Body	OpenSea	other	cluster4	-8.570519084	-0.732717457
55	CCDC141	cg16077818	Body	OpenSea	other	cluster2	-5.316847472	-0.685161412
56	CCDC146	cg13504492	Body	OpenSea	other	cluster2	-2.615277264	-0.697705592
57	CCDC170	cg16835712	Body	OpenSea	other	cluster2	-5.999210592	-0.708243495
58	CCDC63	cg16202755	Body	OpenSea	Proaohancer	cluster2	-5.496137372	-0.504778896
59	CCR1	cg01297500	Body	OpenSea	other	cluster4	-3.829014247	-0.743078336
60	CD109	cg17024944	Body	OpenSea	Proaohancer	cluster2	-2.140032542	-0.662973465
61	CD163	cg18235274	Body	OpenSea	other	cluster2	-12.68912117	-0.623875919
62	CD200R1	cg17069590	Body	OpenSea	other	cluster4	-4.969638027	-0.721525063
63	CD209	cg26704759	Body	OpenSea	other	cluster2	-8.1993428	-0.698201703
64	CD300LF	cg15189015	Body	OpenSea	other	cluster2	-5.558532288	-0.592129742
65	CD300LG	cg14422451	Body	OpenSea	other	cluster4	-9.431134311	-0.578727031
66	CD48	cg10140148	Body	OpenSea	Proaohancer	cluster4	-9.087916854	-0.555985395
67	CD84	cg27304328	Body	OpenSea	other	cluster2	-11.92568789	-0.737240751
68	CELSR1	cg05728600	Body	OpenSea	other	cluster2	-2.0018267	-0.522694231
69	CFAP46	cg24184882	Body	S_Shore	other	cluster2	-2.659192165	-0.308214443
70	CFAP69	cg16453054	Body	OpenSea	other	cluster4	-4.538322403	-0.640988555
71	CFH	cg19575580	Body	OpenSea	other	cluster4	-3.209182422	-0.447029634
72	CHP2	cg04774930	Body	S_Shelf	other	cluster4	-11.42248298	-0.758175323
73	CHRND	cg18316247	Body	OpenSea	other	cluster2	-3.020538026	-0.680940448
74	CHST15	cg24186506	Body	OpenSea	Proaohancer	cluster2	-2.566519501	-0.700360823
75	CLCA4	cg13443976	Body	S_Shelf	other	cluster4	-11.19986258	-0.72915377
76	CLEC2A	cg24640735	Body	OpenSea	other	cluster4	-12.75216934	-0.741677816
77	CLVS2	cg02930689	Body	OpenSea	other	cluster4	-2.642864995	-0.713111908
78	COL12A1	cg06882849	Body	OpenSea	other	cluster2	-3.47961591	-0.713964251
79	COL23A1	cg08684511	Body	OpenSea	other	cluster2	-4.992892924	-0.672850883

80	COL28A1	cg01141095	Body	OpenSea	other	cluster4	-3.196279206	-0.547394238
81	COL4A2-AS1	cg20919265	Body	OpenSea	other	cluster4	-3.924849009	-0.71878351
82	COL6A5	cg01402511	Body	OpenSea	other	cluster4	-11.66946729	-0.826734905
83	COLEC12	cg05489343	Body	OpenSea	other	cluster2	-5.104727177	-0.757426216
84	COLQ	cg27194209	Body	OpenSea	other	cluster2	-2.332166399	-0.599324112
85	CORIN	cg19985724	Body	OpenSea	other	cluster4	-7.59686199	-0.683712775
86	CP	cg27586272	Body	OpenSea	other	cluster2	-5.523028408	-0.781674087
87	CPB1	cg24027602	Body	OpenSea	other	cluster4	-6.33439992	-0.539008419
88	CPQ	cg18544592	Body	OpenSea	Proaohancer	cluster2	-2.125581851	-0.714816652
89	CPZ	cg22657659	Body	OpenSea	other	cluster2	-5.392029283	-0.715056249
90	CRTAM	cg02763813	Body	OpenSea	other	cluster2	-2.856819876	-0.772721363
91	CRYM	cg24969261	Body	N_Shore	other	cluster4	-6.717147994	-0.335485244
92	CUBN	cg15311766	Body	OpenSea	other	cluster2	-3.014153315	-0.638274548
93	CXCL12	cg10079294	Body	OpenSea	other	cluster2	-5.816379988	-0.637656698
94	CYP2C18	cg21636366	Body	OpenSea	other	cluster4	-6.183467594	-0.637321192
95	CYP3A7	cg18504691	Body	OpenSea	other	cluster4	-2.799884006	-0.588253255
96	CYP4F12	cg21114383	Body	OpenSea	other	cluster4	-13.45123456	-0.71298617
97	DAPL1	cg24936299	Body	OpenSea	other	cluster2	-2.270545811	-0.621571262
98	DAPP1	cg10117156	Body	OpenSea	other	cluster4	-7.46374838	-0.639699284
99	DCLK1	cg04541021	Body	OpenSea	other	cluster2	-3.972543119	-0.786651821
100	DEFB1	cg01691696	Body	OpenSea	other	cluster2	-3.269079829	-0.578532887
101	DEGS2	cg01768697	Body	N_Shelf	other	cluster2	-3.55785866	-0.598921688
102	DGAT2	cg23214363	Body	OpenSea	other	cluster2	-3.351295296	-0.48906252
103	DGKG	cg04086468	Body	N_Shore	other	cluster4	-2.203794017	-0.513159242
104	DHRS12	cg26505466	Body	OpenSea	other	cluster2	-2.568979781	-0.578472855
105	DHX58	cg02450064	Body	S_Shore	Proaohancer	cluster2	-2.75762733	-0.61763973
106	DIRC3	cg06523618	Body	OpenSea	Proaohancer	cluster4	-3.000681739	-0.8332273
107	DKK2	cg01421943	Body	OpenSea	other	cluster4	-6.783014147	-0.63443409
108	DLGAP2	cg01571203	Body	S_Shore	other	cluster4	-4.579431028	-0.582240028

109	DNAH3	cg26831772	Body	OpenSea	other	cluster2	-4.761805107	-0.677284113
110	DNASE1L3	cg11387741	Body	OpenSea	other	cluster4	-9.384851514	-0.606302905
111	DOCK8	cg09943198	Body	OpenSea	other	cluster4	-10.89770241	-0.546060943
112	DOK5	cg18073890	Body	OpenSea	Proaohancer	cluster2	-3.819801206	-0.306715374
113	DSG1	cg08415964	Body	OpenSea	other	cluster2	-18.99148439	-0.684059829
114	DSG1-AS1	cg08415964	Body	OpenSea	other	cluster2	-13.53926992	-0.727720032
115	DSG4	cg03229769	Body	OpenSea	other	cluster4	-10.35485139	-0.757791951
116	DTX2P1-UPK3BP1-PMS2P11	cg15802375	Body	N_Shore	other	cluster2	-3.270705673	-0.479778251
117	DTX4	cg16770774	Body	OpenSea	other	cluster4	-7.199533415	-0.804475375
118	EBF2	cg06078892	Body	OpenSea	other	cluster2	-5.633304429	-0.485578733
119	EDAR	cg15391842	Body	OpenSea	other	cluster2	-4.633180914	-0.631408321
120	EFCC1	cg08941083	Body	OpenSea	other	cluster2	-9.044435561	-0.493669882
121	EGFLAM	cg19641786	Body	OpenSea	other	cluster4	-8.940070787	-0.604441855
122	EGFLAM-AS4	cg12558410	Body	OpenSea	other	cluster4	-6.828733717	-0.704465112
123	EIF1B-AS1	cg07537738	Body	OpenSea	other	cluster4	-2.860453089	-0.678577934
124	ELANE	cg11683663	Body	Island	other	cluster4	-5.546023844	-0.52457782
125	ELN	cg20516004	Body	OpenSea	other	cluster2	-2.892237481	-0.538965989
126	EMB	cg04478223	Body	OpenSea	other	cluster4	-2.342261464	-0.684750147
127	ENPP6	cg17343253	Body	N_Shelf	other	cluster2	-2.017739971	-0.672278979
128	EPHA1	cg21019085	Body	OpenSea	other	cluster2	-8.761760281	-0.743507582
129	ERP27	cg14738823	Body	OpenSea	other	cluster4	-5.200097531	-0.638523108
130	ESR1	cg04525653	Body	OpenSea	other	cluster4	-3.324259068	-0.402370638
131	EVPL	cg01013054	Body	Island	other	cluster2	-6.348926633	-0.380868436
132	F7	cg11605551	Body	S_Shore	other	cluster2	-3.15128762	-0.617451396
133	FAM167A-AS1	cg20693444	Body	OpenSea	other	cluster2	-7.668594624	-0.528644986
134	FBN3	cg11453197	Body	OpenSea	other	cluster2	-4.047282149	-0.751108606
135	FCGR2A	cg13817826	Body	OpenSea	Proaohancer	cluster2	-8.427416427	-0.731043476
136	FGF14	cg10071528	Body	OpenSea	other	cluster2	-7.158457567	-0.332977354
137	FGF14-AS1	cg10071528	Body	OpenSea	other	cluster2	-6.768107299	-0.635171519

138	FGF14-IT1	cg10071528	Body	OpenSea	other	cluster2	-6.396574523	-0.635171519
139	FILIP1	cg17944372	Body	OpenSea	Proaohancer	cluster4	-2.972003892	-0.533914694
140	FLT3	cg26128614	Body	OpenSea	other	cluster4	-5.210926182	-0.434439998
141	FLT4	cg06077821	Body	Island	other	cluster4	-6.393055627	-0.816597881
142	FOXP2	cg13886600	Body	OpenSea	other	cluster4	-4.294709964	-0.619790643
143	FRMPD1	cg25802300	Body	OpenSea	other	cluster2	-2.734408571	-0.782917435
144	FRY-AS1	cg03955673	Body	OpenSea	other	cluster2	-2.215214203	-0.382296729
145	GBA3	cg21765117	Body	OpenSea	other	cluster4	-4.754893795	-0.799318356
146	GBP5	cg19925186	Body	OpenSea	other	cluster4	-2.038668674	-0.431802898
147	GCOM1	cg17240133	Body	OpenSea	other	cluster4	-6.937571144	-0.493252497
148	GIPR	cg05333844	Body	N_Shore	other	cluster2	-2.122705983	-0.49801793
149	GLI1	cg00272656	Body	OpenSea	other	cluster2	-2.021821732	-0.628016682
150	GLYATL2	cg21535655	Body	OpenSea	other	cluster4	-5.706914337	-0.703110116
151	GNG12-AS1	cg02453666	Body	OpenSea	other	cluster2	-3.999668367	-0.708113806
152	GNGT2	cg07451034	Body	OpenSea	other	cluster4	-3.665889186	-0.822698437
153	GPR78	cg19005640	Body	Island	other	cluster4	-5.176339234	-0.745392114
154	GREM2	cg01809217	Body	Island	other	cluster4	-4.032216015	-0.373056389
155	GRID1	cg16068023	Body	OpenSea	other	cluster2	-2.564738819	-0.533558863
156	GRID1-AS1	cg16068023	Body	OpenSea	other	cluster2	-5.480933908	-0.533558863
157	GRIK1	cg01856276	Body	OpenSea	other	cluster2	-3.031765926	-0.822580779
158	GRTP1	cg13930683	Body	OpenSea	other	cluster4	-3.024521801	-0.747342141
159	GSG1L	cg10210465	Body	OpenSea	other	cluster2	-2.901277865	-0.630263456
160	GZMB	cg08766149	Body	OpenSea	other	cluster4	-6.423016358	-0.513145226
161	HAS1	cg11425517	Body	N_Shelf	other	cluster4	-3.20016137	-0.650984854
162	HLA-DQB2	cg04345908	Body	S_Shore	other	cluster4	-12.1911289	-0.442282272
163	HLA-DRA	cg26684131	Body	OpenSea	other	cluster4	-15.20107423	-0.499729818
164	HS3ST4	cg23845203	Body	OpenSea	other	cluster2	-6.69778719	-0.687294581
165	IDE	cg24340021	Body	OpenSea	other	cluster2	-2.281275501	-0.653016563
166	IFI44L	cg00458211	Body	OpenSea	other	cluster4	-4.675663675	-0.73376818

167	IFNG-AS1	cg23786157	Body	OpenSea	other	cluster4	-4.916030463	-0.672177262
168	IKZF2	cg00185482	Body	OpenSea	other	cluster4	-3.079971837	-0.664037222
169	IL15RA	cg22870664	Body	OpenSea	other	cluster2	-2.017682015	-0.803173128
170	IL18RAP	cg23027207	Body	OpenSea	other	cluster4	-7.184962162	-0.762083887
171	IL1F10	cg24553058	Body	OpenSea	other	cluster2	-9.201590549	-0.744573659
172	IL1RL1	cg19575169	Body	OpenSea	other	cluster4	-5.177681884	-0.799942817
173	IL7	cg08910705	Body	OpenSea	other	cluster4	-2.435942102	-0.65790849
174	INPP5D	cg14134851	Body	OpenSea	other	cluster4	-10.05875434	-0.73548149
175	IQGAP2	cg14843129	Body	OpenSea	Proaohancer	cluster4	-6.303033745	-0.586400039
176	IRF4	cg11417701	Body	OpenSea	other	cluster4	-2.963006634	-0.37626885
177	IRF8	cg27208925	Body	OpenSea	other	cluster2	-5.591033833	-0.512816887
178	ISM1	cg09495909	Body	OpenSea	other	cluster2	-3.512337389	-0.752478265
179	ITGAM	cg03458229	Body	OpenSea	other	cluster2	-4.532260187	-0.614749426
180	ITGB4	cg09001112	Body	Island	other	cluster2	-4.658920918	-0.770608289
181	ITGB6	cg03583022	Body	OpenSea	other	cluster2	-4.588547867	-0.83401866
182	ITGB7	cg04348509	Body	N_Shelf	other	cluster2	-2.579011689	-0.48910676
183	ITGB8	cg14959425	Body	OpenSea	Proaohancer	cluster2	-5.434845708	-0.622215737
184	IYD	cg02031025	Body	OpenSea	other	cluster4	-8.254665435	-0.707789227
185	JAKMIP3	cg03968041	Body	OpenSea	other	cluster2	-2.608924926	-0.620731831
186	JMJD7-PLA2G4B	cg05018681	Body	OpenSea	other	cluster2	-2.454108989	-0.333903369
187	KBTBD11	cg24598145	Body	N_Shore	other	cluster2	-3.666301572	-0.608738896
188	KCNK1	cg13524022	Body	OpenSea	other	cluster2	-6.990393493	-0.779546444
189	KCNK12	cg01886809	Body	N_Shelf	other	cluster4	-2.777534016	-0.82677303
190	KLC3	cg25481705	Body	Island	other	cluster2	-4.014550549	-0.548943579
191	KLKB1	cg11597515	Body	OpenSea	Proaohancer	cluster4	-3.34251924	-0.471798666
192	KLRG2	cg22894276	Body	N_Shelf	other	cluster2	-6.230289656	-0.660370275
193	LAMA4	cg05476493	Body	OpenSea	Proaohancer	cluster2	-2.550551466	-0.688721839
194	LAMC3	cg16860556	Body	OpenSea	other	cluster2	-7.394384204	-0.597917392
195	LCE5A	cg00011513	Body	N_Shelf	other	cluster4	-11.1529887	-0.697127476

196	LDB2	cg16581308	Body	OpenSea	Proaohancer	cluster4	-9.099390392	-0.686055299
197	LGALS2	cg17929273	Body	OpenSea	other	cluster2	-6.506913778	-0.570184873
198	LGR5	cg25285440	Body	S_Shelf	other	cluster4	-6.507940686	-0.468275002
199	LIFR	cg18204964	Body	OpenSea	other	cluster4	-2.10813462	-0.565307185
200	LIFR-AS1	cg04925574	Body	OpenSea	other	cluster4	-2.517452122	-0.730101382
201	LILRB4	cg20169266	Body	OpenSea	other	cluster4	-8.200553252	-0.444897386
202	LINC00639	cg20859339	Body	OpenSea	other	cluster4	-4.842900025	-0.809919571
203	LINC00887	cg24839536	Body	OpenSea	other	cluster2	-2.70083592	-0.580444105
204	LINC00968	cg17812212	Body	OpenSea	other	cluster4	-7.819610714	-0.782793695
205	LINC01358	cg18581916	Body	OpenSea	other	cluster2	-3.01020433	-0.636265839
206	LINC01375	cg11931329	Body	OpenSea	other	cluster4	-6.363797928	-0.420976895
207	LINC01554	cg06176418	Body	OpenSea	other	cluster2	-6.916876835	-0.629940799
208	LINC01589	cg03780505	Body	OpenSea	other	cluster2	-4.309466482	-0.658291545
209	LIPH	cg05130202	Body	OpenSea	other	cluster2	-6.763158184	-0.63299136
210	LIPM	cg00432203	Body	OpenSea	other	cluster2	-12.24647276	-0.662385766
211	LMX1A	cg07647077	Body	OpenSea	other	cluster2	-3.138389557	-0.728498969
212	LRP1	cg06600276	Body	S_Shore	other	cluster2	-4.016685068	-0.667007887
213	LRP1B	cg06382546	Body	OpenSea	Proaohancer	cluster2	-2.421362134	-0.618146984
214	LY86-AS1	cg00676373	Body	OpenSea	Proaohancer	cluster2	-7.097659785	-0.511778719
215	LY9	cg20662001	Body	OpenSea	other	cluster4	-8.36186427	-0.750236156
216	LYPLAL1-AS1	cg08411363	Body	OpenSea	other	cluster4	-6.913910646	-0.658189031
217	MAN1A1	cg25494796	Body	OpenSea	other	cluster4	-2.443224802	-0.324250689
218	MCTP2	cg05758793	Body	OpenSea	other	cluster4	-5.39666515	-0.582462107
219	MEGF6	cg04549679	Body	N_Shelf	other	cluster2	-2.087151206	-0.338167876
220	MIR3134	cg11740035	Body	OpenSea	other	cluster4	-3.350239169	-0.528078661
221	MIR7853	cg25409638	Body	OpenSea	Proaohancer	cluster2	-5.341775842	-0.576766288
222	MIR99AHG	cg04864577	Body	OpenSea	other	cluster4	-4.716415965	-0.441126213
223	MME	cg14596625	Body	OpenSea	other	cluster4	-2.525343108	-0.748583539
224	MMP2	cg10030250	Body	N_Shelf	other	cluster2	-3.73045317	-0.609748186

225	MPP7	cg16527477	Body	OpenSea	other	cluster4	-2.92465063	-0.666988246
226	MROH7	cg24671160	Body	OpenSea	other	cluster4	-4.864562204	-0.719288303
227	MROH7-TTC4	cg24671160	Body	OpenSea	other	cluster4	-4.720341909	-0.719288303
228	MROH9	cg05225934	Body	OpenSea	other	cluster4	-8.116579631	-0.579655701
229	MS4A4A	cg02587435	Body	OpenSea	other	cluster4	-10.28071326	-0.726454801
230	MS4A6A	cg00580057	Body	OpenSea	other	cluster4	-11.99922421	-0.817063478
231	MTSS1	cg10351284	Body	OpenSea	Proaohancer	cluster4	-2.5798151	-0.533632995
232	MUC5B	cg13649441	Body	S_Shelf	other	cluster2	-2.102598049	-0.740922965
233	MYLK-AS1	cg03635646	Body	OpenSea	other	cluster4	-2.141464131	-0.83985242
234	MYO1F	cg26384903	Body	Island	other	cluster4	-5.226375214	-0.669380154
235	MYO7B	cg16175423	Body	OpenSea	other	cluster2	-2.666818039	-0.531964165
236	NEBL	cg16208523	Body	OpenSea	other	cluster2	-2.003427581	-0.727486076
237	NFATC2	cg27599015	Body	N_Shelf	other	cluster2	-2.1954027	-0.800043282
238	NFIA-AS2	cg26520684	Body	OpenSea	other	cluster2	-3.585209146	-0.471482302
239	NLRP3	cg05615449	Body	OpenSea	other	cluster4	-2.118889181	-0.62294867
240	NLRP6	cg16395700	Body	S_Shore	other	cluster4	-5.473479823	-0.828849818
241	NOS1	cg01207748	Body	OpenSea	other	cluster4	-8.119062153	-0.579490351
242	NOTCH1	cg14364472	Body	N_Shore	other	cluster2	-3.691466901	-0.752969374
243	NOTCH4	cg21993547	Body	OpenSea	other	cluster4	-2.835783289	-0.777228611
244	NR2F2-AS1	cg20650366	Body	OpenSea	other	cluster2	-6.281769124	-0.738263476
245	NTRK2	cg25888138	Body	OpenSea	other	cluster4	-10.56124149	-0.394076663
246	NWD1	cg13135643	Body	OpenSea	other	cluster2	-2.123168271	-0.624112923
247	OPRK1	cg05027549	Body	N_Shelf	other	cluster4	-3.366044417	-0.68512352
248	OSMR-AS1	cg00838695	Body	OpenSea	other	cluster2	-2.845812948	-0.574330777
249	OSTN	cg13913961	Body	OpenSea	Proaohancer	cluster4	-7.273230348	-0.676781768
250	P2RX7	cg22867729	Body	OpenSea	Proaohancer	cluster2	-6.90955347	-0.789379825
251	PAMR1	cg10832281	Body	OpenSea	Proaohancer	cluster2	-3.624215075	-0.569548882
252	PAX8	cg15037451	Body	OpenSea	Proaohancer	cluster4	-3.60097588	-0.809679089
253	PAX8-AS1	cg15037451	Body	OpenSea	Proaohancer	cluster4	-8.62771304	-0.809679089

254	PCSK5	cg15434184	Body	OpenSea	other	cluster4	-3.911755828	-0.407685808
255	PDGFRA	cg14039968	Body	OpenSea	Proaohancer	cluster4	-4.214289213	-0.74708744
256	PDZD2	cg09790035	Body	OpenSea	other	cluster2	-2.050101862	-0.577396221
257	PEBP4	cg19185544	Body	OpenSea	other	cluster2	-4.704064744	-0.625008025
258	PGLYRP4	cg15623013	Body	OpenSea	other	cluster4	-10.60201424	-0.594797531
259	PIK3AP1	cg19687603	Body	OpenSea	other	cluster2	-3.478980035	-0.478084782
260	PIK3C2G	cg20782059	Body	OpenSea	other	cluster4	-9.800250304	-0.777374307
261	PKDCC	cg06839241	Body	OpenSea	Proaohancer	cluster4	-2.602844961	-0.407987056
262	PLAGL1	cg06271237	Body	OpenSea	other	cluster4	-2.183197928	-0.360804617
263	PLCH1	cg15609035	Body	OpenSea	other	cluster4	-2.299048429	-0.878757625
264	PLCL1	cg04362425	Body	OpenSea	other	cluster4	-2.082405966	-0.796251674
265	PLCXD3	cg05638200	Body	OpenSea	other	cluster4	-2.485577676	-0.486348902
266	PLXNA4	cg22531284	Body	OpenSea	other	cluster2	-6.367440243	-0.561691939
267	PODNL1	cg10165008	Body	Island	other	cluster4	-2.811843619	-0.676213919
268	PON1	cg22798737	Body	OpenSea	other	cluster4	-7.017683812	-0.740436509
269	PON3	cg14239045	Body	OpenSea	other	cluster4	-7.603754627	-0.605663353
270	POSTN	cg23202139	Body	OpenSea	other	cluster4	-4.71907777	-0.752549764
271	PPARGC1A	cg18744982	Body	OpenSea	other	cluster4	-4.472299199	-0.525101293
272	PPL	cg18148948	Body	OpenSea	other	cluster4	-5.824446404	-0.724342334
273	PPP1R14C	cg08927004	Body	OpenSea	other	cluster2	-2.90187097	-0.760901696
274	PPP2R2B	cg20540357	Body	OpenSea	other	cluster2	-3.939892333	-0.59447511
275	PRB4	cg09679558	Body	OpenSea	other	cluster4	-3.177115262	-0.59493815
276	PRDM16	cg03082523	Body	N_Shore	other	cluster2	-4.527115424	-0.654946136
277	PREX2	cg14532680	Body	OpenSea	other	cluster4	-8.859985497	-0.516353009
278	PROS1	cg27044832	Body	OpenSea	other	cluster2	-2.915237421	-0.673063578
279	PRRX1	cg24513381	Body	OpenSea	Proaohancer	cluster4	-4.659601919	-0.735188886
280	PZP	cg17944251	Body	OpenSea	Proaohancer	cluster4	-4.730110542	-0.706987125
281	RAB44	cg22259123	Body	OpenSea	other	cluster2	-5.419076412	-0.750520218
282	RAMP1	cg13452095	Body	OpenSea	other	cluster2	-4.303753387	-0.624597224

283	RAPGEF4	cg04571833	Body	OpenSea	other	cluster4	-3.440346868	-0.72873699
284	RASIP1	cg26370886	Body	Island	other	cluster2	-2.385183651	-0.686797648
285	RDH10-AS1	cg02613338	Body	OpenSea	other	cluster2	-2.387460369	-0.828396619
286	RGS6	cg18127398	Body	OpenSea	Proaohancer	cluster4	-10.09858571	-0.596321917
287	RNASET2	cg25258033	Body	N_Shore	other	cluster2	-2.18913108	-0.601771326
288	RNF175	cg18630485	Body	OpenSea	other	cluster4	-8.212602472	-0.715502758
289	RNF222	cg04482798	Body	Island	Proaohancer	cluster4	-6.142359528	-0.766479897
290	RNLS	cg01307228	Body	OpenSea	other	cluster4	-2.262559038	-0.511267369
291	RORA	cg03461216	Body	OpenSea	other	cluster2	-6.518038115	-0.756919866
292	ROS1	cg08237608	Body	OpenSea	other	cluster2	-5.911991061	-0.653083481
293	RSPO1	cg11305072	Body	OpenSea	other	cluster2	-10.96363096	-0.789377464
294	RUFY4	cg08908213	Body	OpenSea	other	cluster2	-5.923177751	-0.595324328
295	RYR1	cg15804426	Body	S_Shore	other	cluster2	-2.296981654	-0.780128776
296	RYR3	cg24364120	Body	OpenSea	other	cluster4	-5.548869906	-0.573955936
297	SAMD5	cg02233217	Body	OpenSea	other	cluster4	-2.349892102	-0.670937308
298	SCARA5	cg24762759	Body	N_Shelf	other	cluster2	-14.56532233	-0.400635282
299	SCML4	cg09682385	Body	OpenSea	Proaohancer	cluster2	-3.052923134	-0.593645864
300	SCUBE2	cg12743500	Body	OpenSea	other	cluster2	-4.910757908	-0.324843329
301	SDC1	cg10329928	Body	OpenSea	Proaohancer	cluster2	-3.279571192	-0.558748166
302	SEC14L4	cg23383138	Body	OpenSea	other	cluster2	-10.17288907	-0.615156451
303	SEC14L5	cg00835521	Body	Island	other	cluster2	-2.071023561	-0.81696718
304	SELL	cg07784955	Body	OpenSea	Proaohancer	cluster4	-8.111385235	-0.419640639
305	SELP	cg06812356	Body	OpenSea	other	cluster2	-9.891801995	-0.721812557
306	SEPT4-AS1	cg06976613	Body	OpenSea	other	cluster4	-2.483827237	-0.746920047
307	SERPINA5	cg14423617	Body	OpenSea	other	cluster2	-9.164228649	-0.828428399
308	SERPINB13	cg20471736	Body	OpenSea	other	cluster4	-8.457223208	-0.391897586
309	SFMBT2	cg07763467	Body	OpenSea	Proaohancer	cluster4	-5.393531956	-0.723525951
310	SGPP2	cg06379883	Body	OpenSea	other	cluster4	-2.178477517	-0.488803953
311	SH3TC2	cg25246741	Body	OpenSea	other	cluster4	-2.789709125	-0.814639033

312	SIGLEC14	cg16034991	Body	OpenSea	other	cluster4	-6.053087191	-0.753442572
313	SLC1A3	cg12506575	Body	OpenSea	other	cluster4	-2.2138185	-0.693159857
314	SLC1A7	cg10137383	Body	OpenSea	Proaohancer	cluster2	-3.734997052	-0.712843395
315	SLC22A25	cg16358817	Body	OpenSea	other	cluster2	-6.103248243	-0.609748966
316	SLC22A3	cg01739295	Body	S_Shelf	other	cluster4	-6.271331443	-0.494387396
317	SLC22A7	cg26558036	Body	OpenSea	other	cluster4	-3.161990183	-0.691048844
318	SLC25A21	cg21252728	Body	OpenSea	other	cluster2	-2.049616391	-0.612212917
319	SLC26A1	cg02776128	Body	Island	other	cluster4	-2.467644255	-0.318262232
320	SLC26A3	cg19889126	Body	OpenSea	other	cluster4	-5.404566199	-0.724461872
321	SLC27A1	cg15507500	Body	N_Shelf	other	cluster4	-2.526122377	-0.478037461
322	SLC2A9	cg03841832	Body	OpenSea	Proaohancer	cluster4	-3.911434222	-0.584268168
323	SLC44A4	cg10969178	Body	OpenSea	Proaohancer	cluster2	-2.945886609	-0.552555736
324	SLC44A5	cg04677630	Body	OpenSea	other	cluster2	-4.852026731	-0.624908075
325	SLC47A1	cg16887170	Body	OpenSea	other	cluster2	-4.263278014	-0.717305729
326	SLC47A2	cg19555478	Body	OpenSea	other	cluster2	-6.731078289	-0.738197213
327	SLC5A10	cg27548595	Body	N_Shelf	other	cluster2	-2.166038253	-0.74756554
328	SLC6A2	cg03519481	Body	OpenSea	other	cluster2	-4.216791106	-0.626437507
329	SLC9A9	cg26537443	Body	OpenSea	other	cluster4	-5.609200419	-0.420412527
330	SLIT3	cg06071396	Body	OpenSea	other	cluster2	-5.131294629	-0.664694369
331	SMOC2	cg05621218	Body	OpenSea	Proaohancer	cluster4	-9.957385818	-0.786804459
332	SNED1	cg14767619	Body	N_Shore	other	cluster2	-2.978680534	-0.385561003
333	SNTG2	cg14389442	Body	OpenSea	other	cluster2	-10.18628183	-0.678604014
334	SNX29P2	cg11282846	Body	OpenSea	other	cluster2	-5.883675872	-0.709015651
335	SOAT2	cg26268968	Body	OpenSea	Proaohancer	cluster4	-4.860955749	-0.498832397
336	SPAG17	cg03501167	Body	OpenSea	other	cluster4	-2.583746821	-0.8105571
337	SPATA13	cg18656962	Body	OpenSea	other	cluster4	-3.205426706	-0.750696909
338	SPDEF	cg00293599	Body	OpenSea	other	cluster2	-3.159671732	-0.431228433
339	SPON1	cg17658341	Body	OpenSea	Proaohancer	cluster2	-5.961514409	-0.600958763
340	SPTA1	cg16041062	Body	OpenSea	other	cluster4	-4.184844743	-0.741869941

341	SSPN	cg13909661	Body	OpenSea	other	cluster2	-2.158724158	-0.666865889
342	ST6GAL1	cg00127725	Body	OpenSea	other	cluster2	-2.673903075	-0.599590229
343	STXBP5-AS1	cg09426038	Body	OpenSea	other	cluster4	-2.281183004	-0.577158905
344	STXBP6	cg16125853	Body	OpenSea	other	cluster2	-2.917701466	-0.514047543
345	SULF2	cg08998048	Body	OpenSea	other	cluster2	-4.030817239	-0.663354297
346	SVEP1	cg18613092	Body	OpenSea	other	cluster4	-3.978345126	-0.723408094
347	SYNE3	cg17445458	Body	OpenSea	other	cluster2	-2.651649717	-0.635609144
348	SYT17	cg18051449	Body	OpenSea	other	cluster2	-4.376784622	-0.814098696
349	SYT6	cg07510237	Body	OpenSea	other	cluster2	-4.119386972	-0.761539685
350	TG	cg01598421	Body	OpenSea	Proaohancer	cluster2	-5.851279828	-0.698440415
351	TGFBR3	cg02391982	Body	OpenSea	Proaohancer	cluster2	-3.757475741	-0.683794039
352	TGM3	cg20117447	Body	Island	other	cluster4	-13.54439167	-0.769882457
353	THEG	cg23463127	Body	N_Shore	other	cluster2	-3.071795661	-0.334947247
354	TIGIT	cg09755142	Body	OpenSea	other	cluster4	-4.056081343	-0.696562173
355	TMEM51-AS1	cg26178194	Body	OpenSea	Proaohancer	cluster4	-3.543229137	-0.386002329
356	TMPRSS13	cg00575786	Body	OpenSea	other	cluster2	-10.09884996	-0.632964692
357	TMPRSS4	cg01728830	Body	OpenSea	other	cluster2	-5.442908824	-0.581364105
358	TMTC1	cg18602710	Body	OpenSea	Proaohancer	cluster2	-2.650971713	-0.584092456
359	TNFRSF1B	cg15526535	Body	OpenSea	Proaohancer	cluster2	-2.498422217	-0.712444674
360	TNFSF11	cg10371412	Body	OpenSea	other	cluster4	-6.079310962	-0.582587289
361	TNFSF8	cg14187242	Body	OpenSea	other	cluster2	-8.440852398	-0.69414375
362	TNNI3K	cg08718880	Body	OpenSea	Proaohancer	cluster4	-4.40623907	-0.673745629
363	TNXB	cg26695758	Body	Island	Proaohancer	cluster2	-7.65756013	-0.639129509
364	TPRXL	cg23145243	Body	OpenSea	other	cluster2	-6.960524008	-0.787293228
365	TRIM22	cg12313315	Body	OpenSea	other	cluster4	-4.961161632	-0.68924383
366	TRPC6	cg06726982	Body	OpenSea	other	cluster4	-2.958823493	-0.673291156
367	TRPM1	cg07964177	Body	OpenSea	other	cluster2	-10.28081573	-0.637512692
368	TRPV6	cg24444878	Body	OpenSea	other	cluster4	-12.25012687	-0.73501183
369	TSHZ2	cg14830513	Body	OpenSea	other	cluster2	-2.494902519	-0.549344519

370	TSNAX-DISC1	cg16177633	Body	OpenSea	other	cluster2	-2.829325498	-0.636324141
371	TSPAN8	cg21758446	Body	OpenSea	other	cluster4	-6.267075251	-0.359180995
372	TTC22	cg22167738	Body	OpenSea	other	cluster2	-7.244438162	-0.489479807
373	TXK	cg01105703	Body	OpenSea	other	cluster4	-3.427481765	-0.663210267
374	UBQLN3	cg10517815	Body	OpenSea	other	cluster2	-6.790120839	-0.707476523
375	UMODL1	cg00785029	Body	OpenSea	other	cluster2	-2.745793627	-0.71411757
376	UNC13C	cg00101164	Body	OpenSea	other	cluster4	-4.38652862	-0.486663418
377	UNC13D	cg06769725	Body	OpenSea	other	cluster2	-2.472760324	-0.573182841
378	UPK1A	cg09968702	Body	N_Shelf	other	cluster2	-4.752364571	-0.59850635
379	VIPR2	cg19965595	Body	OpenSea	other	cluster2	-8.11543318	-0.772680477
380	VPS13D	cg00059737	Body	OpenSea	Proaohancer	cluster2	-2.01080216	-0.623204097
381	WFDC1	cg02138682	Body	OpenSea	other	cluster4	-6.04520511	-0.59438202
382	XDH	cg22597494	Body	OpenSea	other	cluster2	-2.113245537	-0.698439796
383	ZBTB16	cg00387390	Body	OpenSea	other	cluster2	-12.92224523	-0.629380158

Group IV

S.No.	hgnc_symbol	IlmnID	Refgrp	Relation_to_UCSC_CpG_Island	Proaohancer	Cluster	MS1vsNSk_LogFC	MS1_meth
1	ACOX2	cg16587010	Promoter	OpenSea	Proaohancer	cluster1	-6.220273532	0.361005935
2	ACSS1	cg02086310	Promoter	S_Shore	Proaohancer	cluster1	-3.437788681	0.477359621
3	ACVRL1	cg25749512	Promoter	Island	Proaohancer	cluster1	-6.908447289	0.688972425
4	ADAM33	cg11858114	Promoter	Island	Proaohancer	cluster3	-5.843056669	0.445330736
5	ADCY7	cg19594772	Promoter	OpenSea	Proaohancer	cluster1	-2.106402045	0.548550183
6	ADD3	cg08294376	Promoter	OpenSea	Proaohancer	cluster1	-2.096087198	0.526527285
7	ADRA1A	cg17963840	Promoter	Island	Proaohancer	cluster3	-2.771782032	0.653686935
8	AGT	cg07502417	Promoter	OpenSea	Proaohancer	cluster1	-3.711477216	0.609450593
9	AKNA	cg08827579	Promoter	OpenSea	Proaohancer	cluster1	-2.060798719	0.584358253
10	ALOX15	cg15843823	Promoter	S_Shore	Proaohancer	cluster3	-7.790790759	0.478832305

11	ALS2CL	cg25104512	Promoter	Island	Proaohancer	cluster3	-7.570695784	0.342744275
12	ANGPT1	cg11745019	Promoter	OpenSea	Proaohancer	cluster1	-2.758723565	0.796635629
13	ANKRD29	cg03354908	Promoter	S_Shore	Proaohancer	cluster1	-4.793682053	0.671157059
14	AQP1	cg26923410	Promoter	OpenSea	Proaohancer	cluster1	-7.886934918	0.376874811
15	ASB2	cg13485310	Promoter	OpenSea	Proaohancer	cluster1	-2.316112797	0.305624956
16	BCL6	cg04339396	Promoter	S_Shore	Proaohancer	cluster1	-2.425919956	0.561690957
17	BHMT	cg02286091	Promoter	OpenSea	Proaohancer	cluster1	-2.879894659	0.843835828
18	C2orf40	cg14535980	Promoter	N_Shore	Proaohancer	cluster1	-5.305935197	0.81037766
19	C5orf56	cg11576424	Promoter	S_Shelf	Proaohancer	cluster1	-2.093591511	0.698812765
20	CALCRL	cg21350115	Promoter	OpenSea	Proaohancer	cluster1	-4.08869328	0.697754865
21	CCDC60	cg13254584	Promoter	Island	Proaohancer	cluster1	-2.052180364	0.589432733
22	CCRL2	cg03477080	Promoter	OpenSea	Proaohancer	cluster3	-2.092164509	0.476163747
23	CD300A	cg02554050	Promoter	OpenSea	Proaohancer	cluster1	-5.089634827	0.437414169
24	CD302	cg03432176	Promoter	S_Shore	Proaohancer	cluster3	-2.364446039	0.440595225
25	CD36	cg24275848	Promoter	OpenSea	Proaohancer	cluster1	-5.745540829	0.499758879
26	CD8A	cg12606911	Promoter	Island	Proaohancer	cluster1	-2.521690161	0.824048637
27	CFTR	cg25509184	Promoter	OpenSea	Proaohancer	cluster1	-6.520102696	0.795062563
28	CH25H	cg07899729	Promoter	S_Shore	Proaohancer	cluster3	-7.884215827	0.393137979
29	CHL1	cg13925692	Promoter	Island	Proaohancer	cluster3	-4.805085031	0.729976319
30	CHRM5	cg26855208	Promoter	S_Shore	Proaohancer	cluster3	-3.225458981	0.455236216
31	CLEC14A	cg08139247	Promoter	Island	Proaohancer	cluster1	-10.86851241	0.707531562
32	CLEC3B	cg25576364	Promoter	OpenSea	Proaohancer	cluster1	-11.74319779	0.654317536
33	CLU	cg24428058	Promoter	N_Shore	Proaohancer	cluster1	-2.17895723	0.584492348
34	CMKLR1	cg22927613	Promoter	OpenSea	Proaohancer	cluster1	-11.46235313	0.625727771
35	COL21A1	cg15703636	Promoter	S_Shore	Proaohancer	cluster3	-4.005678006	0.329666144
36	COX7A1	cg24929925	Promoter	Island	Proaohancer	cluster1	-2.810647867	0.658847135
37	CPXM2	cg03630088	Promoter	S_Shore	Proaohancer	cluster3	-11.40717809	0.69765928
38	CYP27A1	cg22811307	Promoter	N_Shore	Proaohancer	cluster1	-2.825757453	0.786130229
39	DAAM2	cg01985396	Promoter	S_Shore	Proaohancer	cluster3	-3.642529302	0.343923457

40	DES	cg03583111	Promoter	Island	Proaohancer	cluster1	-10.16781823	0.883855469
41	DGKA	cg07679948	Promoter	Island	Proaohancer	cluster1	-2.992066331	0.61822325
42	DIO2	cg16254394	Promoter	OpenSea	Proaohancer	cluster1	-2.519926497	0.454187573
43	DLEU7	cg08274637	Promoter	Island	Proaohancer	cluster1	-2.552051414	0.584662723
44	DNAJC15	cg12201399	Promoter	N_Shore	Proaohancer	cluster3	-3.422721475	0.435256483
45	DNASE1L2	cg09607446	Promoter	Island	Proaohancer	cluster1	-4.565223038	0.355798076
46	DRD2	cg12176709	Promoter	S_Shore	Proaohancer	cluster3	-2.607713623	0.34342763
47	DSP	cg03814872	Promoter	N_Shore	Proaohancer	cluster3	-6.197371278	0.501324348
48	ELMO1	cg09775742	Promoter	OpenSea	Proaohancer	cluster3	-2.135266543	0.407981861
49	ELMOD1	cg09445027	Promoter	OpenSea	Proaohancer	cluster1	-4.59102025	0.548463544
50	EMILIN1	cg05654832	Promoter	N_Shelf	Proaohancer	cluster1	-4.160409637	0.57635342
51	EMP2	cg27178401	Promoter	OpenSea	Proaohancer	cluster1	-2.774544225	0.634800455
52	F13A1	cg13462398	Promoter	OpenSea	Proaohancer	cluster1	-11.62480434	0.323101521
53	FAM107A	cg23827531	Promoter	OpenSea	Proaohancer	cluster3	-8.494159013	0.505533358
54	FAM166B	cg04245250	Promoter	OpenSea	Proaohancer	cluster1	-4.883611033	0.400173976
55	FAM20A	cg15761609	Promoter	S_Shore	Proaohancer	cluster3	-3.431377558	0.403831233
56	FBP1	cg12211605	Promoter	Island	Proaohancer	cluster3	-6.240203765	0.540672494
57	FERD3L	cg25691167	Promoter	Island	Proaohancer	cluster1	-5.183337198	0.925568663
58	FERMT1	cg07718903	Promoter	S_Shore	Proaohancer	cluster1	-4.098695608	0.59579914
59	FHL5	cg03043157	Promoter	OpenSea	Proaohancer	cluster3	-3.298659756	0.372860497
60	FMOD	cg21089380	Promoter	OpenSea	Proaohancer	cluster1	-8.365336223	0.735750506
61	FNDC1	cg09107912	Promoter	Island	Proaohancer	cluster1	-4.114946216	0.636756025
62	FOXI2	cg02523640	Promoter	Island	Proaohancer	cluster3	-4.869057687	0.830177549
63	FRZB	cg04037038	Promoter	Island	Proaohancer	cluster3	-4.459412886	0.406049381
64	GALM	cg05604112	Promoter	OpenSea	Proaohancer	cluster1	-3.048769531	0.364481078
65	GDF10	cg07773116	Promoter	Island	Proaohancer	cluster3	-4.732344323	0.375713073
66	GFRA2	cg15794228	Promoter	Island	Proaohancer	cluster1	-8.195088885	0.662250335
67	GIMAP1	cg12914657	Promoter	N_Shelf	Proaohancer	cluster1	-10.01305658	0.611537819
68	GIMAP5	cg02955377	Promoter	OpenSea	Proaohancer	cluster1	-9.598854286	0.62836203

69	GSN	cg14399183	Promoter	OpenSea	Proaohancer	cluster1	-4.201712007	0.579221929
70	GSTM5	cg24467349	Promoter	OpenSea	Proaohancer	cluster1	-7.496430459	0.548109053
71	H6PD	cg26754508	Promoter	S_Shelf	Proaohancer	cluster1	-2.543146221	0.582202849
72	HAAO	cg26907578	Promoter	Island	Proaohancer	cluster3	-3.324681186	0.835781233
73	HAND2	cg01580681	Promoter	N_Shore	Proaohancer	cluster3	-2.259300042	0.402280343
74	HCK	cg17508991	Promoter	N_Shore	Proaohancer	cluster1	-4.407423195	0.868433883
75	HCLS1	cg02167021	Promoter	OpenSea	Proaohancer	cluster1	-6.456190688	0.626916656
76	HHATL	cg07864883	Promoter	OpenSea	Proaohancer	cluster1	-4.996387088	0.481260861
77	HIST1H4F	cg12260798	Promoter	N_Shore	Proaohancer	cluster1	-6.022310874	0.629776806
78	IL33	cg02305659	Promoter	OpenSea	Proaohancer	cluster1	-12.63248445	0.35749738
79	ITM2B	cg04657044	Promoter	N_Shore	Proaohancer	cluster1	-2.910638414	0.552869743
80	KCNE3	cg17718679	Promoter	Island	Proaohancer	cluster1	-4.769559863	0.688552638
81	KCNJ15	cg08257579	Promoter	OpenSea	Proaohancer	cluster1	-6.411664471	0.5896031
82	KIAA1217	cg23881119	Promoter	OpenSea	Proaohancer	cluster1	-2.009198783	0.593101706
83	KLK10	cg11170179	Promoter	Island	Proaohancer	cluster3	-4.718978858	0.408114702
84	KLK13	cg20104456	Promoter	S_Shore	Proaohancer	cluster1	-9.772438483	0.807061962
85	KRT72	cg22200736	Promoter	S_Shore	Proaohancer	cluster1	-11.19970182	0.698655644
86	LHCGR	cg20156659	Promoter	Island	Proaohancer	cluster1	-5.300585995	0.806398881
87	LINC01094	cg24390961	Promoter	OpenSea	Proaohancer	cluster1	-3.168648671	0.722593184
88	LINC01354	cg13023668	Promoter	OpenSea	Proaohancer	cluster1	-3.19156421	0.650959124
89	LINC-PINT	cg03072329	Promoter	OpenSea	Proaohancer	cluster1	-3.285250973	0.769906537
90	LRCOL1	cg04759104	Promoter	Island	Proaohancer	cluster1	-2.644825129	0.718272258
91	LRG1	cg11502375	Promoter	N_Shelf	Proaohancer	cluster1	-7.631186684	0.568194722
92	LRRC15	cg13321259	Promoter	OpenSea	Proaohancer	cluster1	-4.721060192	0.506737204
93	LRRN2	cg16367429	Promoter	OpenSea	Proaohancer	cluster1	-2.45444208	0.367031164
94	LTC4S	cg05408831	Promoter	N_Shore	Proaohancer	cluster3	-3.155709352	0.734970266
95	LY96	cg13213009	Promoter	OpenSea	Proaohancer	cluster1	-4.489465261	0.449402192
96	MAP3K8	cg19596273	Promoter	S_Shore	Proaohancer	cluster3	-2.111322336	0.730028988
97	MCHR1	cg21342728	Promoter	OpenSea	Proaohancer	cluster3	-4.007408844	0.56171936

98	MEOX2	cg02969141	Promoter	OpenSea	Proaohancer	cluster3	-2.254661155	0.550306828
99	MGST2	cg11124069	Promoter	OpenSea	Proaohancer	cluster1	-2.272174887	0.630932159
100	MT1M	cg18909924	Promoter	Island	Proaohancer	cluster1	-6.444106229	0.858892732
101	NCALD	cg00680551	Promoter	N_Shore	Proaohancer	cluster3	-3.65062751	0.70180905
102	NDRG2	cg18081258	Promoter	Island	Proaohancer	cluster3	-5.891219692	0.382527154
103	NKAPL	cg18675097	Promoter	Island	Proaohancer	cluster1	-5.078374174	0.879180279
104	NMU	cg15112355	Promoter	S_Shore	Proaohancer	cluster1	-3.030775928	0.376739613
105	NOD1	cg06557644	Promoter	OpenSea	Proaohancer	cluster1	-2.585976448	0.618244474
106	NOSTRIN	cg25694915	Promoter	OpenSea	Proaohancer	cluster1	-2.632468052	0.582791692
107	NPBWR1	cg26205771	Promoter	N_Shore	Proaohancer	cluster3	-3.899302422	0.797605728
108	NPY5R	cg15586439	Promoter	N_Shore	Proaohancer	cluster3	-8.471497841	0.334138727
109	NUPR1	cg15745560	Promoter	OpenSea	Proaohancer	cluster1	-4.486618834	0.624550396
110	OSR1	cg23215476	Promoter	Island	Proaohancer	cluster3	-4.182351783	0.461416341
111	OVOL1	cg23220533	Promoter	Island	Proaohancer	cluster1	-3.598794309	0.468768652
112	PARP15	cg23442853	Promoter	N_Shore	Proaohancer	cluster1	-6.772000375	0.612253572
113	PAX3	cg18352427	Promoter	Island	Proaohancer	cluster1	-8.445926805	0.475354578
114	PAX9	cg04415798	Promoter	S_Shore	Proaohancer	cluster1	-3.314550846	0.52313382
115	PCDHB3	cg01925738	Promoter	N_Shore	Proaohancer	cluster1	-2.136050972	0.543362857
116	PDK4	cg06070414	Promoter	S_Shore	Proaohancer	cluster1	-4.128588434	0.359319394
117	PELI1	cg11727482	Promoter	N_Shore	Proaohancer	cluster1	-2.636876357	0.561106978
118	PHF24	cg19422661	Promoter	S_Shelf	Proaohancer	cluster1	-4.131856581	0.550929578
119	PHYHIP	cg13093774	Promoter	Island	Proaohancer	cluster3	-4.905382315	0.706220457
120	PIK3IP1	cg12273325	Promoter	S_Shelf	Proaohancer	cluster1	-3.599271679	0.728347001
121	PLA2G7	cg16536824	Promoter	S_Shore	Proaohancer	cluster3	-3.93964728	0.320324531
122	PLA2R1	cg20257553	Promoter	S_Shore	Proaohancer	cluster1	-6.792498455	0.861003124
123	PLSCR4	cg12727940	Promoter	S_Shore	Proaohancer	cluster3	-3.315541215	0.37804096
124	PPFIBP2	cg04723364	Promoter	N_Shore	Proaohancer	cluster1	-3.079647832	0.589693526
125	RANBP3L	cg21870145	Promoter	OpenSea	Proaohancer	cluster1	-4.606191028	0.599791591
126	RASAL3	cg05504085	Promoter	OpenSea	Proaohancer	cluster3	-4.81117468	0.398615722

127	RASSF9	cg27035734	Promoter	OpenSea	Proaohancer	cluster3	-5.622360404	0.651573198
128	RAX	cg00814752	Promoter	S_Shore	Proaohancer	cluster3	-3.720680293	0.557981706
129	RBM47	cg27335760	Promoter	OpenSea	Proaohancer	cluster1	-5.413925872	0.511778573
130	RBPJL	cg08654443	Promoter	Island	Proaohancer	cluster3	-6.999892559	0.604098963
131	RFX8	cg27255239	Promoter	S_Shore	Proaohancer	cluster1	-2.653623817	0.443163785
132	RGMA	cg04697454	Promoter	S_Shore	Proaohancer	cluster1	-5.956787949	0.636100922
133	RHOD	cg15693066	Promoter	Island	Proaohancer	cluster1	-5.181915702	0.899289238
134	RNF180	cg17621438	Promoter	N_Shore	Proaohancer	cluster3	-4.464688983	0.369010587
135	SCN9A	cg22872396	Promoter	OpenSea	Proaohancer	cluster1	-2.410332496	0.44592004
136	SCUBE1	cg01237056	Promoter	Island	Proaohancer	cluster1	-2.343489821	0.852541275
137	SERPINF1	cg04955351	Promoter	OpenSea	Proaohancer	cluster1	-5.220772418	0.706517719
138	SGCD	cg12904904	Promoter	OpenSea	Proaohancer	cluster1	-2.874857686	0.561795872
139	SH3D19	cg16827707	Promoter	OpenSea	Proaohancer	cluster1	-2.369977457	0.533452677
140	SLC16A5	cg09300114	Promoter	Island	Proaohancer	cluster1	-2.372328547	0.646880595
141	SLC22A31	cg03280573	Promoter	N_Shore	Proaohancer	cluster1	-3.829991758	0.332254233
142	SLC22A8	cg11200963	Promoter	OpenSea	Proaohancer	cluster1	-6.086562658	0.525833649
143	SLC25A2	cg07039560	Promoter	Island	Proaohancer	cluster1	-5.676764121	0.396324502
144	SLC27A6	cg20801875	Promoter	N_Shore	Proaohancer	cluster3	-2.935738533	0.409523493
145	SLC2A14	cg06645921	Promoter	Island	Proaohancer	cluster1	-8.849529709	0.815504288
146	SLC38A11	cg03464655	Promoter	OpenSea	Proaohancer	cluster3	-3.678503169	0.703996597
147	SLC38A4	cg21428710	Promoter	OpenSea	Proaohancer	cluster3	-7.07234462	0.594576303
148	SOD3	cg03577139	Promoter	OpenSea	Proaohancer	cluster1	-9.548989268	0.605233029
149	SORBS1	cg20959701	Promoter	OpenSea	Proaohancer	cluster1	-2.187619384	0.654409137
150	SOX8	cg05520409	Promoter	Island	Proaohancer	cluster3	-3.432797142	0.652907101
151	SP8	cg27191852	Promoter	N_Shelf	Proaohancer	cluster1	-5.585105721	0.606681731
152	SPARCL1	cg19466563	Promoter	OpenSea	Proaohancer	cluster1	-7.930702639	0.588078596
153	STAR	cg09630404	Promoter	OpenSea	Proaohancer	cluster1	-3.569776442	0.684714351
154	STAT5A	cg08246644	Promoter	N_Shore	Proaohancer	cluster1	-2.562472671	0.755553786
155	STEAP4	cg07719679	Promoter	OpenSea	Proaohancer	cluster1	-13.50037156	0.408898723

156	SYCE1	cg20087519	Promoter	Island	Proaohancer	cluster1	-4.186075617	0.744515158
157	SYCN	cg22290648	Promoter	Island	Proaohancer	cluster3	-6.350001669	0.38880022
158	TAL1	cg06955484	Promoter	N_Shore	Proaohancer	cluster1	-6.23909221	0.609565808
159	TBX15	cg05329979	Promoter	Island	Proaohancer	cluster1	-2.886139482	0.623987614
160	TBX5	cg06911121	Promoter	Island	Proaohancer	cluster1	-9.665655877	0.621551496
161	TBX5-AS1	cg16517851	Promoter	Island	Proaohancer	cluster1	-9.981595085	0.756603736
162	THBS2	cg26280713	Promoter	OpenSea	Proaohancer	cluster3	-4.092342451	0.679513116
163	THRB	cg14974411	Promoter	OpenSea	Proaohancer	cluster1	-4.152993233	0.474915751
164	TLR1	cg08888038	Promoter	OpenSea	Proaohancer	cluster3	-3.105136849	0.428275175
165	TMC1	cg14327393	Promoter	OpenSea	Proaohancer	cluster1	-2.909493274	0.58480687
166	TMEM144	cg18366748	Promoter	N_Shore	Proaohancer	cluster1	-3.637294512	0.597088013
167	TMEM63A	cg10459226	Promoter	S_Shore	Proaohancer	cluster1	-2.188298344	0.45548606
168	TNFRSF18	cg15706223	Promoter	Island	Proaohancer	cluster1	-4.419980863	0.707319571
169	TNS1	cg03323067	Promoter	OpenSea	Proaohancer	cluster1	-2.97613385	0.812460066
170	UBXN10	cg11651220	Promoter	S_Shore	Proaohancer	cluster3	-3.243947547	0.570833189
171	UBXN10-AS1	cg25826297	Promoter	S_Shore	Proaohancer	cluster3	-5.281238599	0.670250184
172	UPB1	cg12492885	Promoter	Island	Proaohancer	cluster1	-4.332674931	0.755442205
173	WFIKKN2	cg10408284	Promoter	OpenSea	Proaohancer	cluster1	-4.528311888	0.572648182
174	WNT2B	cg17569616	Promoter	N_Shore	Proaohancer	cluster1	-2.396875559	0.487096616
175	WNT3	cg02017282	Promoter	Island	Proaohancer	cluster1	-2.385224046	0.672927744
176	YBX2	cg16009970	Promoter	N_Shore	Proaohancer	cluster3	-2.025602755	0.425353871
177	ZBTB20	cg03556243	Promoter	OpenSea	Proaohancer	cluster1	-2.504962496	0.635900316
178	ZC3H12D	cg18082788	Promoter	OpenSea	Proaohancer	cluster1	-4.894601882	0.571176357
179	ZNF366	cg21915770	Promoter	OpenSea	Proaohancer	cluster3	-7.736336773	0.755669679

Supplemental Table 3: List of genes regulated by DNA methylation in MCC13

Group I

	hgnc_symbol	ID	Location	CpG Island	Proaohancer	Cluster	MCC13vsNSk_LogFC	MCC13_meth
1	ACLY	cg14583225	Promoter	N_Shore	Proaohancer	cluster2	2.809886346	-0.581543328
2	ADAMTS20	cg24606791	Promoter	S_Shore	Proaohancer	cluster4	3.588466951	-0.339936608
3	ADCY1	cg11175660	Promoter	N_Shore	Proaohancer	cluster2	3.649333334	-0.502368973
4	AFAP1	cg17438830	Promoter	OpenSea	Proaohancer	cluster2	3.271162719	-0.517049613
5	ANKRD34B	cg26305827	Promoter	S_Shore	Proaohancer	cluster2	3.383254964	-0.424833481
6	ARC	cg08387463	Promoter	Island	Proaohancer	cluster2	5.536715967	-0.462418203
7	ASPHD1	cg02488299	Promoter	S_Shore	Proaohancer	cluster2	3.524290314	-0.764118978
8	ATF5	cg23878206	Promoter	N_Shore	Proaohancer	cluster2	3.722962005	-0.300919045
9	C19orf81	cg14844855	Promoter	OpenSea	Proaohancer	cluster4	2.285785066	-0.752282359
10	CALN1	cg14955982	Promoter	OpenSea	Proaohancer	cluster2	2.133996386	-0.527088314
11	CDK5R2	cg23452259	Promoter	N_Shore	Proaohancer	cluster2	11.89211855	-0.564744346
12	CENPN	cg03284308	Promoter	N_Shore	Proaohancer	cluster2	3.769466917	-0.579939883
13	CHGA	cg01901466	Promoter	N_Shore	Proaohancer	cluster4	8.803026942	-0.735469058
14	DTYMK	cg00969323	Promoter	S_Shore	Proaohancer	cluster2	2.68798113	-0.45534253
15	ELFN1	cg17549927	Promoter	OpenSea	Proaohancer	cluster2	4.33299006	-0.439096415
16	EYA2	cg06209200	Promoter	OpenSea	Proaohancer	cluster2	4.399235591	-0.393313956
17	GAL3ST1	cg08215954	Promoter	OpenSea	Proaohancer	cluster2	3.001037497	-0.446101
18	GMNN	cg04570316	Promoter	S_Shore	Proaohancer	cluster2	2.476433966	-0.550807574
19	GNB1	cg27021587	Promoter	OpenSea	Proaohancer	cluster2	2.522247608	-0.492233854
20	GREM1	cg17327198	Promoter	OpenSea	Proaohancer	cluster2	3.275587632	-0.405542385
21	GRXCR1	cg20682222	Promoter	OpenSea	Proaohancer	cluster4	9.873559684	-0.742433963
22	HABP2	cg18833140	Promoter	OpenSea	Proaohancer	cluster2	4.256864465	-0.556030906
23	HIST1H2BO	cg12629515	Promoter	N_Shore	Proaohancer	cluster2	4.595673686	-0.666075529
24	HIST1H3J	cg12629515	Promoter	N_Shore	Proaohancer	cluster2	4.578997472	-0.666075529
25	HMGA1	cg02654940	Promoter	S_Shore	Proaohancer	cluster2	4.052625313	-0.586496629

26	HTR1D	cg08872493	Promoter	OpenSea	Proaohancer	cluster4	7.960193833	-0.402431335
27	KCNA3	cg13925011	Promoter	Island	Proaohancer	cluster2	2.437030839	-0.614014184
28	KCND2	cg01338148	Promoter	N_Shore	Proaohancer	cluster2	3.60313519	-0.64405535
29	KRT20	cg06082664	Promoter	OpenSea	Proaohancer	cluster2	6.478928137	-0.321327983
30	LBR	cg22945457	Promoter	N_Shelf	Proaohancer	cluster4	2.260612278	-0.357612564
31	LHFPL5	cg22184996	Promoter	S_Shore	Proaohancer	cluster2	2.005269052	-0.536790307
32	LINGO1	cg06061712	Promoter	OpenSea	Proaohancer	cluster2	2.7506593	-0.455175162
33	MC5R	cg14179575	Promoter	Island	Proaohancer	cluster4	2.037829072	-0.540743514
34	MCM5	cg15009484	Promoter	N_Shore	Proaohancer	cluster2	2.458353055	-0.507191743
35	METTL11B	cg03192897	Promoter	OpenSea	Proaohancer	cluster4	2.912635154	-0.494044157
36	MICAL2	cg27286609	Promoter	OpenSea	Proaohancer	cluster2	2.159512711	-0.392302986
37	MKI67	cg22260973	Promoter	S_Shore	Proaohancer	cluster2	4.474632121	-0.407723418
38	MKRN3	cg13415551	Promoter	OpenSea	Proaohancer	cluster2	3.505923895	-0.498602334
39	MMEL1	cg19633446	Promoter	S_Shelf	Proaohancer	cluster2	2.169538	-0.604689525
40	MPPED1	cg09929856	Promoter	Island	Proaohancer	cluster2	3.491249315	-0.547699307
41	MSLNL	cg02485642	Promoter	Island	Proaohancer	cluster2	3.25444153	-0.64363604
42	MYT1	cg23856293	Promoter	OpenSea	Proaohancer	cluster2	9.713431728	-0.69515139
43	NEFM	cg14139227	Promoter	N_Shore	Proaohancer	cluster2	8.404233611	-0.603563091
44	NEUROD6	cg03535916	Promoter	S_Shelf	Proaohancer	cluster2	8.390428305	-0.543558514
45	NIPSNAP1	cg00939432	Promoter	S_Shore	Proaohancer	cluster2	2.80667731	-0.690512723
46	NUP62	cg23878206	Promoter	N_Shore	Proaohancer	cluster2	2.883546034	-0.300919045
47	NYAP2	cg02395751	Promoter	OpenSea	Proaohancer	cluster2	7.91958827	-0.493842014
48	ODC1	cg15075241	Promoter	N_Shore	Proaohancer	cluster2	4.066409957	-0.572552035
49	PAK2	cg15166739	Promoter	N_Shore	Proaohancer	cluster4	2.217366873	-0.316974059
50	PIGW	cg00482489	Promoter	S_Shore	Proaohancer	cluster2	2.106878635	-0.313609946
51	PKIB	cg11889875	Promoter	OpenSea	Proaohancer	cluster2	2.927021565	-0.526523952
52	PNLDC1	cg15758783	Promoter	Island	Proaohancer	cluster4	2.044418244	-0.380912093
53	PRAME	cg19292908	Promoter	N_Shore	Proaohancer	cluster4	9.868340641	-0.787311172
54	PTPRD	cg09418843	Promoter	OpenSea	Proaohancer	cluster4	3.315304422	-0.535760644

55	RBFOX3	cg07966558	Promoter	OpenSea	Proaohancer	cluster2	4.497784766	-0.66190871
56	RFPL1S	cg06182770	Promoter	OpenSea	Proaohancer	cluster2	5.238479433	-0.39298452
57	RMI1	cg14327541	Promoter	S_Shore	Proaohancer	cluster2	2.882030499	-0.330869025
58	RPH3A	cg22453284	Promoter	OpenSea	Proaohancer	cluster2	3.766518524	-0.511284381
59	SARDH	cg12655879	Promoter	OpenSea	Proaohancer	cluster2	3.084019241	-0.487319779
60	SEPHS2	cg26697065	Promoter	N_Shore	Proaohancer	cluster2	2.58553936	-0.530835392
61	SERPINA6	cg06058015	Promoter	OpenSea	Proaohancer	cluster4	2.209926394	-0.628088357
62	SH3BP4	cg08858245	Promoter	S_Shelf	Proaohancer	cluster2	3.109402607	-0.351593623
63	SNAP25	cg09910691	Promoter	S_Shore	Proaohancer	cluster2	6.304894115	-0.558494387
64	SNRPB2	cg25926513	Promoter	S_Shore	Proaohancer	cluster2	2.414135324	-0.543609674
65	ST18	cg10750742	Promoter	OpenSea	Proaohancer	cluster2	5.042054163	-0.375538432
66	SVOP	cg22733953	Promoter	OpenSea	Proaohancer	cluster2	5.939117074	-0.673239949
67	TBCB	cg04628320	Promoter	Island	Proaohancer	cluster2	2.416080312	-0.42003089
68	TCF3	cg08430680	Promoter	S_Shelf	Proaohancer	cluster2	3.295221023	-0.392603729
69	TMEM51	cg06869505	Promoter	OpenSea	Proaohancer	cluster2	2.40333063	-0.747342429
70	TP53	cg07760161	Promoter	N_Shore	Proaohancer	cluster2	2.348669256	-0.539226293
71	UBLCP1	cg02884181	Promoter	N_Shore	Proaohancer	cluster2	2.005973645	-0.383167649
72	ULBP3	cg17728956	Promoter	S_Shore	Proaohancer	cluster4	2.954347295	-0.482218807
73	WWTR1	cg16853336	Promoter	OpenSea	Proaohancer	cluster4	2.33625108	-0.443735425
74	ZNF536	cg26406587	Promoter	N_Shelf	Proaohancer	cluster4	3.472314924	-0.477938945

Group II

S.No.	hgnc_symbol	IlmnID	Refgrp	Relation_to_UCSC_CpG_Island	Proaohancer	Cluster	MCC13vsNSk_LogFC	MCC13_meth
1	UCK2	cg06252523	Body	OpenSea	other	cluster3	2.158414606	0.30524472
2	SHISA7	cg21719704	Body	N_Shelf	other	cluster1	2.091130024	0.305351142
3	KCNC2	cg06563089	Body	Island	other	cluster1	3.072159575	0.308859882
4	VSTM2B	cg15436715	Body	OpenSea	other	cluster1	2.59568974	0.312506075
5	DPYSL5	cg23332900	Body	OpenSea	other	cluster3	12.2584198	0.317078551

6	POT1-AS1	cg17105921	Body	OpenSea	other	cluster3	2.093956185	0.318165701
7	KSR2	cg05738715	Body	OpenSea	Proaohancer	cluster1	5.064006249	0.319247303
8	TMEM151B	cg03498886	Body	Island	other	cluster3	3.341261115	0.322477538
9	BMPER	cg06711255	Body	OpenSea	Proaohancer	cluster3	3.89608615	0.324275731
10	PRKCA	cg15212295	Body	OpenSea	Proaohancer	cluster3	3.384601997	0.327001923
11	CACNA2D2	cg26325335	Body	Island	Proaohancer	cluster1	3.229221513	0.327559659
12	NRXN3	cg12874088	Body	OpenSea	other	cluster1	3.352998141	0.327712783
13	SYNJ2	cg04786164	Body	OpenSea	Proaohancer	cluster1	2.311449631	0.327878809
14	PTBP1	cg02984314	Body	Island	other	cluster3	2.86876669	0.335609625
15	SMTNL2	cg26089877	Body	Island	other	cluster1	2.547719495	0.336189997
16	GDF6	cg05690644	Body	Island	Proaohancer	cluster1	6.895556133	0.33636865
17	RCC2	cg01904812	Body	Island	other	cluster3	2.384628545	0.340409297
18	NFIC	cg15658793	Body	Island	other	cluster3	2.211166974	0.342986545
19	SLC8A2	cg23048399	Body	Island	other	cluster3	4.827664233	0.353987478
20	DLG4	cg13768953	Body	N_Shelf	other	cluster3	2.295901932	0.358661761
21	BARX1	cg12179826	Body	Island	other	cluster1	8.909584892	0.364605937
22	PPM1D	cg26127413	Body	OpenSea	other	cluster3	2.364208662	0.369644687
23	DNMT3A	cg20303441	Body	N_Shelf	Proaohancer	cluster3	2.138226544	0.370872263
24	TMC2	cg12385739	Body	OpenSea	other	cluster1	3.861693853	0.38079856
25	CELSR3	cg02858118	Body	Island	Proaohancer	cluster1	3.882047429	0.382567056
26	CDH22	cg25484135	Body	N_Shore	other	cluster1	2.423224138	0.386436142
27	PCBP3	cg08455194	Body	OpenSea	Proaohancer	cluster3	3.350596998	0.388145778
28	TMEM132E	cg16065538	Body	Island	other	cluster1	5.573395437	0.398108862
29	ACTN4	cg03315866	Body	OpenSea	other	cluster1	2.068134283	0.399319575
30	CSMD3	cg06230615	Body	OpenSea	other	cluster1	5.636482147	0.400124147
31	DIAPH3	cg24289237	Body	OpenSea	other	cluster1	4.253583611	0.402170394
32	RIMS2	cg14750367	Body	OpenSea	other	cluster1	6.266856541	0.404245157
33	NPHS1	cg20655980	Body	OpenSea	other	cluster1	7.974227484	0.413897025
34	ADAM19	cg13667243	Body	Island	Proaohancer	cluster1	3.993958146	0.414997292

35	POU6F2	cg18850127	Body	OpenSea	other	cluster3	2.331044678	0.41519392
36	HOXC11	cg08857479	Body	S_Shore	other	cluster1	2.263329463	0.417842778
37	LINC00870	cg10162076	Body	OpenSea	other	cluster1	2.272112611	0.432088376
38	CBFA2T3	cg27435133	Body	S_Shore	other	cluster3	3.686267703	0.432174077
39	FAM171A1	cg02217247	Body	OpenSea	Proaohancer	cluster3	3.422341548	0.432392096
40	APBA2	cg09077934	Body	Island	other	cluster1	3.454927353	0.437968039
41	DPP6	cg11330315	Body	OpenSea	other	cluster1	2.576042636	0.438344952
42	TMEM104	cg01285145	Body	N_Shore	Proaohancer	cluster3	3.032004444	0.440033651
43	TPH2	cg02754084	Body	OpenSea	Proaohancer	cluster1	2.331236289	0.440229127
44	PTPRN2	cg05124021	Body	S_Shore	other	cluster1	3.727723021	0.440921008
45	SMAD3	cg16430601	Body	OpenSea	Proaohancer	cluster1	2.259697658	0.44177968
46	NOCT	cg26954135	Body	OpenSea	other	cluster3	2.170637787	0.44400243
47	CA10	cg14444957	Body	OpenSea	Proaohancer	cluster1	6.008275738	0.448992125
48	LHX9	cg22443762	Body	Island	other	cluster1	9.010187664	0.453552504
49	TRPA1	cg01414726	Body	N_Shore	other	cluster3	2.90900797	0.454271718
50	CCDC178	cg06156162	Body	OpenSea	other	cluster1	4.475101631	0.455282308
51	PAX2	cg26156687	Body	Island	other	cluster1	11.91975333	0.456281163
52	PHOX2B	cg10192893	Body	Island	Proaohancer	cluster1	4.725228864	0.456401069
53	CCNJL	cg23309856	Body	OpenSea	other	cluster1	2.955476394	0.45644126
54	CBLN1	cg04438525	Body	N_Shore	other	cluster1	2.136612845	0.459456714
55	GALNT10	cg26013791	Body	Island	other	cluster3	2.162330839	0.460718005
56	LINC01122	cg23641231	Body	OpenSea	other	cluster1	3.371467061	0.463029171
57	CACNA1I	cg17802949	Body	Island	other	cluster1	4.880914886	0.463301027
58	SPTBN4	cg02576468	Body	Island	Proaohancer	cluster1	2.843868023	0.476520548
59	SSBP3	cg21159128	Body	OpenSea	other	cluster3	2.148866699	0.478739915
60	NT5DC2	cg07757611	Body	S_Shore	other	cluster3	2.525472062	0.481573642
61	MEX3A	cg11717597	Body	Island	other	cluster3	6.49004538	0.488953416
62	SIM2	cg14626259	Body	N_Shore	Proaohancer	cluster3	4.989345264	0.491989394
63	SFTA3	cg26002259	Body	N_Shore	Proaohancer	cluster1	3.699115393	0.495598789

64	CCDC40	cg05159799	Body	S_Shelf	other	cluster1	2.498846275	0.498226074
65	AP3B2	cg25584930	Body	OpenSea	other	cluster1	2.994736939	0.499559169
66	GRIK2	cg22851944	Body	S_Shore	other	cluster1	2.19876218	0.500724134
67	MYO15A	cg02458237	Body	OpenSea	other	cluster1	4.87931455	0.502462015
68	CRYBA2	cg17054386	Body	N_Shelf	other	cluster1	10.47821264	0.506320539
69	EN2	cg16935980	Body	Island	other	cluster1	6.272424169	0.506889941
70	SKOR1	cg21611830	Body	Island	Proaohancer	cluster1	3.862787415	0.508437379
71	HSF2BP	cg16942004	Body	OpenSea	Proaohancer	cluster1	2.43223348	0.508968751
72	FGF5	cg14349667	Body	S_Shore	other	cluster1	7.220388881	0.514293342
73	NXPH1	cg03836586	Body	S_Shelf	other	cluster1	3.904292876	0.522101624
74	CACNA1A	cg14911505	Body	OpenSea	other	cluster1	5.872313508	0.527763426
75	KHDRBS3	cg00753112	Body	OpenSea	Proaohancer	cluster1	2.255161854	0.528562237
76	RGS7	cg04120815	Body	OpenSea	other	cluster1	4.487237654	0.52936656
77	FAM189B	cg27615508	Body	S_Shore	other	cluster3	2.525984406	0.52943412
78	VAX1	cg26263263	Body	N_Shore	other	cluster1	2.832350974	0.529855507
79	DLX5	cg09359114	Body	N_Shore	other	cluster1	2.708688552	0.53019165
80	INSRR	cg16438688	Body	Island	other	cluster1	3.053469338	0.53135375
81	NTRK1	cg16438688	Body	Island	other	cluster1	2.105327413	0.53135375
82	RAP1GAP	cg11531272	Body	OpenSea	other	cluster1	2.117532382	0.532087942
83	ZNF827	cg05267427	Body	OpenSea	Proaohancer	cluster1	2.654549493	0.53310034
84	PRSS23	cg24069172	Body	OpenSea	other	cluster1	2.232421338	0.533894648
85	SYT16	cg01491926	Body	OpenSea	other	cluster1	6.729802263	0.535580587
86	KIRREL3	cg01775559	Body	OpenSea	other	cluster1	3.301040723	0.537584202
87	SRRM3	cg17783368	Body	S_Shelf	other	cluster1	3.77451946	0.539427533
88	DDN	cg00757327	Body	Island	other	cluster3	6.272928982	0.546927308
89	BRSK2	cg17429870	Body	N_Shore	Proaohancer	cluster1	3.415980181	0.549375483
90	CPNE7	cg16616467	Body	Island	other	cluster1	4.711138328	0.550296308
91	SCN8A	cg21974358	Body	Island	Proaohancer	cluster1	4.045940189	0.553607579
92	KCNH3	cg08876130	Body	OpenSea	other	cluster1	2.982861835	0.555357255

93	SCRT2	cg05073843	Body	Island		other	cluster1	8.035083207	0.558145986
94	TRPM8	cg23993463	Body	Island		other	cluster1	4.433861704	0.558312116
95	MARCH4	cg20409685	Body	OpenSea		other	cluster1	7.831597932	0.563225738
96	TBX20	cg00598758	Body	N_Shore		other	cluster1	8.11739641	0.564174164
97	HOXD13	cg24914355	Body	S_Shore		other	cluster1	3.491706024	0.564826535
98	ST6GALNAC5	cg17405164	Body	OpenSea		Proaohancer	cluster1	4.242904812	0.56727042
99	WT1	cg25782229	Body	N_Shore		other	cluster1	7.32817135	0.570947957
100	LHX2	cg14425564	Body	OpenSea		other	cluster1	2.113523925	0.573435164
101	TFAP2B	cg23015341	Body	Island		other	cluster3	2.455777369	0.576181921
102	BTBD17	cg12131414	Body	Island		other	cluster1	7.511519985	0.58036232
103	CTNND2	cg01937840	Body	OpenSea		Proaohancer	cluster3	4.052588947	0.581868451
104	CCDC33	cg25590826	Body	OpenSea		other	cluster1	3.879236178	0.585946211
105	LHX1	cg15291243	Body	Island		other	cluster1	10.7863773	0.587516946
106	SDK1	cg17458659	Body	OpenSea		other	cluster1	2.031375589	0.590481267
107	NKX2-1	cg13980454	Body	N_Shore		other	cluster1	7.87532171	0.59084843
108	KIF26A	cg12924430	Body	Island		other	cluster1	2.87491505	0.590987837
109	COL22A1	cg15909548	Body	OpenSea		other	cluster1	7.76029815	0.592271082
110	TLX1	cg23340017	Body	N_Shore		other	cluster3	4.090513255	0.593137258
111	GRIN2D	cg23444265	Body	Island		Proaohancer	cluster1	5.138870137	0.597490738
112	TBR1	cg12520549	Body	S_Shore		Proaohancer	cluster1	2.182031556	0.598676522
113	TFAP2D	cg13654594	Body	S_Shore		Proaohancer	cluster1	8.965097025	0.600078755
114	ERC2	cg12029753	Body	OpenSea		Proaohancer	cluster1	3.425634526	0.600762426
115	SYT1	cg24588941	Body	OpenSea		other	cluster1	4.884478436	0.605161244
116	NKX6-1	cg25830182	Body	N_Shore		other	cluster1	9.755399652	0.606667521
117	NRXN2	cg10940462	Body	Island		Proaohancer	cluster1	3.490005558	0.610170471
118	KCNH2	cg16817992	Body	Island		other	cluster1	6.035686734	0.612517906
119	MAST1	cg06537894	Body	Island		other	cluster1	3.770685843	0.620850202
120	KIF19	cg18259003	Body	Island		other	cluster1	7.028825535	0.623667049
121	LINC01399	cg23932633	Body	OpenSea		other	cluster1	5.922663796	0.626935646

122	TP73	cg17163168	Body	OpenSea	other	cluster1	3.777689821	0.629978946
123	LMX1B	cg14225031	Body	Island	Proaohancer	cluster1	2.169632184	0.633958355
124	CMIP	cg07137626	Body	OpenSea	Proaohancer	cluster1	2.171226314	0.63809024
125	PRKAR1B	cg07454365	Body	N_Shelf	other	cluster1	3.155132068	0.640731205
126	SLC12A5	cg06132069	Body	Island	other	cluster1	2.659683686	0.643999681
127	NXPH4	cg04850731	Body	Island	other	cluster1	4.40166925	0.644372551
128	HMX2	cg22886575	Body	Island	other	cluster1	5.897183774	0.645061401
129	KIRREL2	cg01588826	Body	S_Shore	Proaohancer	cluster1	8.471575701	0.646470661
130	RUNDC3A	cg02115818	Body	S_Shore	other	cluster1	2.772722172	0.648669408
131	SLC4A4	cg11363527	Body	OpenSea	Proaohancer	cluster1	4.715909453	0.653290022
132	LDB3	cg11911560	Body	OpenSea	Proaohancer	cluster3	2.457423138	0.654709881
133	SH2B2	cg07512361	Body	Island	other	cluster1	2.82748807	0.666068444
134	VAX2	cg10617784	Body	Island	Proaohancer	cluster1	3.181907529	0.670586756
135	ITPKA	cg22888055	Body	S_Shore	other	cluster1	2.328321241	0.673711406
136	DLGAP3	cg18113535	Body	Island	Proaohancer	cluster1	5.89308567	0.68084685
137	ANK1	cg15706250	Body	Island	other	cluster1	5.516262197	0.682005326
138	SLC17A6	cg09150064	Body	S_Shore	Proaohancer	cluster1	10.16609645	0.685558573
139	SRRM4	cg01254303	Body	Island	other	cluster1	6.945169192	0.687257678
140	GNA12	cg16569650	Body	N_Shore	other	cluster1	2.669062032	0.687692874
141	BARHL1	cg15033348	Body	S_Shore	other	cluster1	6.623360571	0.698501116
142	SIX3	cg16509777	Body	S_Shore	other	cluster1	10.05546693	0.709058205
143	LRTM2	cg18952026	Body	OpenSea	Proaohancer	cluster1	6.457128062	0.713106533
144	C1QL1	cg02395846	Body	Island	Proaohancer	cluster1	6.420426649	0.719323448
145	B4GALNT1	cg21361094	Body	Island	Proaohancer	cluster1	2.787522284	0.720906878
146	ISLR2	cg01178680	Body	Island	other	cluster1	6.785731533	0.728264214
147	PAX6	cg15215534	Body	S_Shore	other	cluster1	3.335197359	0.737342756
148	CDKN2A	cg14348664	Body	N_Shore	other	cluster1	8.059200526	0.737721753
149	PAX5	cg20815683	Body	Island	other	cluster1	9.23153081	0.743067628
150	ETS1	cg01900413	Body	Island	Proaohancer	cluster1	2.587797722	0.743929844

151	GRM4	cg00070899	Body	S_Shore	other	cluster1	8.290701383	0.744841276
152	WNK4	cg20653128	Body	N_Shore	other	cluster1	2.220538818	0.74551795
153	CERKL	cg01293771	Body	OpenSea	other	cluster1	2.249833178	0.747123798
154	MNX1	cg02440420	Body	Island	other	cluster1	7.850133652	0.754196136
155	HMGA2	cg15394142	Body	OpenSea	Proaohancer	cluster3	8.63624365	0.756731635
156	POU4F2	cg21419180	Body	Island	Proaohancer	cluster3	11.00103867	0.758527222
157	SEZ6L2	cg06890619	Body	Island	Proaohancer	cluster1	5.707960976	0.771166592
158	ONECUT2	cg15276629	Body	OpenSea	other	cluster1	8.284861332	0.771514029
159	NRGN	cg10938436	Body	Island	Proaohancer	cluster1	3.908214294	0.777834898
160	SLC32A1	cg12451099	Body	Island	Proaohancer	cluster1	6.491940307	0.789163444
161	ISL2	cg13749815	Body	Island	other	cluster3	6.46431272	0.80131534
162	GPRIN1	cg02098752	Body	Island	other	cluster1	5.75489852	0.803701157
163	MAPK8IP2	cg09877744	Body	Island	other	cluster1	3.686299043	0.817626864
164	ZFR2	cg03126579	Body	Island	other	cluster3	2.088162805	0.842876457

Group III

S.No.	hgnc_symbol	IlmnID	Refgrp	Relation_to_UCSC_CpG_Island	Proaohancer	Cluster	MCC13vsNSk_LogFC	MCC13_meth
1	ABCA13	cg08329397	Body	OpenSea	other	cluster4	-2.434563157	-0.371260988
2	ABCA4	cg19196920	Body	OpenSea	other	cluster4	-4.343129108	-0.435573878
3	ABCC8	cg12401398	Body	OpenSea	Proaohancer	cluster2	-5.128285461	-0.309742192
4	ACPP	cg25111496	Body	OpenSea	other	cluster2	-9.332798584	-0.332131409
5	ADAMTS17	cg02589109	Body	OpenSea	other	cluster4	-2.006286347	-0.312503577
6	ADAMTS2	cg03259333	Body	N_Shore	other	cluster2	-6.46707384	-0.590545766
7	ADCY2	cg00247998	Body	OpenSea	other	cluster2	-9.157573129	-0.589627385
8	ADGB	cg14870813	Body	OpenSea	other	cluster4	-3.186831457	-0.622900541
9	AGBL1	cg21205579	Body	OpenSea	other	cluster4	-5.815843462	-0.331992552
10	AKR1C3	cg08894837	Body	OpenSea	other	cluster4	-4.055704958	-0.307173009
11	AKR1C4	cg12357263	Body	OpenSea	other	cluster4	-5.046819467	-0.595809135

12	ALDH8A1	cg18761400	Body	OpenSea	other	cluster2	-2.402296027	-0.518637555
13	ANO1	cg27260891	Body	OpenSea	other	cluster4	-9.319480315	-0.554950281
14	ANO2	cg20202463	Body	OpenSea	other	cluster2	-4.911843365	-0.409314084
15	AOC1	cg08801691	Body	S_Shore	other	cluster4	-8.868440019	-0.473580468
16	ARHGEF7-AS1	cg18829020	Body	OpenSea	other	cluster4	-5.42931315	-0.545308344
17	ATP13A4	cg23997451	Body	OpenSea	other	cluster2	-8.015169698	-0.649578544
18	BCAS1	cg02450219	Body	OpenSea	other	cluster2	-5.599753151	-0.338278546
19	BPIFC	cg12406374	Body	OpenSea	other	cluster2	-9.568425339	-0.683778357
20	C22orf34	cg09640001	Body	OpenSea	other	cluster2	-6.306669424	-0.339324746
21	C3orf52	cg08451978	Body	OpenSea	Proaohancer	cluster2	-3.03891077	-0.812607854
22	CACNA2D4	cg01214459	Body	OpenSea	other	cluster2	-3.05409231	-0.596600621
23	CCDC141	cg16077818	Body	OpenSea	other	cluster2	-5.825475107	-0.53953132
24	CCDC170	cg16835712	Body	OpenSea	other	cluster2	-5.125707648	-0.356493147
25	CCDC80	cg02468355	Body	OpenSea	Proaohancer	cluster4	-2.322698106	-0.32479285
26	CD163	cg18235274	Body	OpenSea	other	cluster2	-12.69670167	-0.378575593
27	CD209	cg26704759	Body	OpenSea	other	cluster2	-8.269857364	-0.612790726
28	CD300LF	cg15189015	Body	OpenSea	other	cluster2	-5.565940826	-0.554791093
29	CD48	cg10140148	Body	OpenSea	Proaohancer	cluster4	-9.095486814	-0.457538183
30	CD84	cg27304328	Body	OpenSea	other	cluster2	-11.93326719	-0.47411551
31	CD86	cg04387658	Body	OpenSea	other	cluster4	-6.423830968	-0.522404084
32	CDH4	cg12708821	Body	OpenSea	Proaohancer	cluster2	-2.372245135	-0.596700357
33	CFAP46	cg24184882	Body	S_Shore	other	cluster2	-2.420921471	-0.628334967
34	CFAP69	cg16453054	Body	OpenSea	other	cluster4	-3.925858549	-0.488060272
35	CHP2	cg01776298	Body	S_Shore	other	cluster4	-13.38858912	-0.576894859
36	CLEC2A	cg24640735	Body	OpenSea	other	cluster4	-12.75975064	-0.686317191
37	CNGB3	cg27312173	Body	OpenSea	other	cluster4	-2.256346403	-0.706551977
38	COL28A1	cg01141095	Body	OpenSea	other	cluster4	-2.730719125	-0.356788157
39	CORIN	cg19985724	Body	OpenSea	other	cluster4	-7.553448003	-0.531127633
40	CPZ	cg22657659	Body	OpenSea	other	cluster2	-2.112912662	-0.489121353

41	CR1L	cg13314228	Body	OpenSea	other	cluster4	-2.794797406	-0.440920305
42	CYP2W1	cg24286812	Body	Island	other	cluster2	-4.567494869	-0.414841331
43	CYP4F12	cg21114383	Body	OpenSea	other	cluster4	-11.56576718	-0.715048122
44	DCC	cg05207924	Body	OpenSea	other	cluster4	-3.381498913	-0.685465388
45	DGAT2	cg23214363	Body	OpenSea	other	cluster2	-3.083720535	-0.333979768
46	DLGAP2	cg24188870	Body	N_Shore	other	cluster4	-4.770049135	-0.442115537
47	DNAH3	cg26831772	Body	OpenSea	other	cluster2	-4.684351452	-0.453527002
48	DSC3	cg24898592	Body	OpenSea	other	cluster4	-3.049485345	-0.432641149
49	DTX4	cg16770774	Body	OpenSea	other	cluster4	-7.262070105	-0.667433755
50	EDAR	cg05729352	Body	OpenSea	other	cluster2	-5.398128717	-0.374834823
51	EGFLAM	cg25344265	Body	OpenSea	other	cluster4	-9.034635097	-0.605970213
52	ELANE	cg11683663	Body	Island	other	cluster4	-8.218590498	-0.424779745
53	ENPP6	cg17343253	Body	N_Shelf	other	cluster2	-2.061816289	-0.424186743
54	EYS	cg25099688	Body	OpenSea	other	cluster4	-3.31786625	-0.590244616
55	F7	cg11605551	Body	S_Shore	other	cluster2	-2.606056335	-0.460558684
56	FAM167A-AS1	cg20693444	Body	OpenSea	other	cluster2	-7.676137898	-0.478207908
57	FBN3	cg11453197	Body	OpenSea	other	cluster2	-4.111808981	-0.459453114
58	FLT4	cg06077821	Body	Island	other	cluster4	-6.072500519	-0.654768293
59	FRMPD1	cg25802300	Body	OpenSea	other	cluster2	-2.656142059	-0.487507925
60	GLYATL2	cg21535655	Body	OpenSea	other	cluster4	-4.725310923	-0.34324048
61	GNG12-AS1	cg02453666	Body	OpenSea	other	cluster2	-2.320633977	-0.325042327
62	GPR26	cg13677800	Body	Island	other	cluster2	-3.210038705	-0.345945737
63	GPR78	cg19005640	Body	Island	other	cluster4	-3.103305004	-0.393456913
64	GREM2	cg01809217	Body	Island	other	cluster4	-3.91328787	-0.303754052
65	GRIK1	cg01856276	Body	OpenSea	other	cluster2	-2.101806784	-0.469876209
66	GRIN3B	cg01806181	Body	Island	other	cluster2	-2.238384533	-0.505922154
67	HAS1	cg11425517	Body	N_Shelf	other	cluster4	-3.67441931	-0.561028872
68	HS3ST4	cg23845203	Body	OpenSea	other	cluster2	-6.705297223	-0.629027645
69	IL1RL1	cg19575169	Body	OpenSea	other	cluster4	-5.179850257	-0.352778573

70	IQGAP2	cg14843129	Body	OpenSea		Proaohancer	cluster4	-4.181642329	-0.50306238
71	IRF4	cg11417701	Body	OpenSea		other	cluster4	-2.955814191	-0.354488806
72	ISM1	cg09495909	Body	OpenSea		other	cluster2	-3.515613829	-0.34473578
73	ITGAM	cg03458229	Body	OpenSea		other	cluster2	-3.975628557	-0.326717921
74	ITGB8	cg09606036	Body	OpenSea		other	cluster4	-5.435630917	-0.470081397
75	JAKMIP3	cg13196806	Body	N_Shelf		other	cluster2	-2.906025542	-0.665477905
76	KBTBD11	cg24598145	Body	N_Shore		other	cluster2	-3.175262393	-0.575699538
77	KCNK1	cg13524022	Body	OpenSea		other	cluster2	-5.469952114	-0.548541204
78	KCNK12	cg01886809	Body	N_Shelf		other	cluster4	-2.396641707	-0.639845816
79	KEL	cg12427933	Body	OpenSea		other	cluster4	-2.064585069	-0.616957053
80	LCE5A	cg00011513	Body	N_Shelf		other	cluster4	-8.510364084	-0.483534323
81	LDB2	cg16581308	Body	OpenSea		Proaohancer	cluster4	-12.36321437	-0.67311458
82	LDHD	cg07565228	Body	Island		other	cluster2	-4.880352718	-0.683885668
83	LILRB4	cg20169266	Body	OpenSea		other	cluster4	-8.208111173	-0.669305993
84	LINC00968	cg17812212	Body	OpenSea		other	cluster4	-5.970634778	-0.659566956
85	LINC01375	cg11931329	Body	OpenSea		other	cluster4	-6.371288589	-0.663558255
86	LINC01589	cg03780505	Body	OpenSea		other	cluster2	-2.836461378	-0.665574529
87	LIPM	cg00432203	Body	OpenSea		other	cluster2	-12.25405289	-0.744711239
88	LMX1A	cg07647077	Body	OpenSea		other	cluster2	-4.203109937	-0.51061554
89	LY86-AS1	cg00676373	Body	OpenSea		Proaohancer	cluster2	-5.121602453	-0.530965744
90	LY9	cg20662001	Body	OpenSea		other	cluster4	-6.488583751	-0.528706224
91	MAL	cg07765975	Body	OpenSea		other	cluster2	-7.255291313	-0.721235849
92	MIR7853	cg25409638	Body	OpenSea		Proaohancer	cluster2	-5.349151129	-0.482529474
93	MROH9	cg20705671	Body	OpenSea		other	cluster4	-4.568969408	-0.318460424
94	MS4A4A	cg02587435	Body	OpenSea		other	cluster4	-10.2882888	-0.631282903
95	MS4A6A	cg00580057	Body	OpenSea		other	cluster4	-12.0068039	-0.613401402
96	MUC5B	cg13649441	Body	S_Shelf		other	cluster2	-4.247947478	-0.711123994
97	NAALADL2	cg25488905	Body	OpenSea		other	cluster4	-2.976394294	-0.514136288
98	NEBL	cg07815856	Body	OpenSea		other	cluster2	-2.118716426	-0.42608476

99	NFATC2	cg27599015	Body	N_Shelf	other	cluster2	-2.29301914	-0.673201781
100	NLRP6	cg16395700	Body	S_Shore	other	cluster4	-5.480880269	-0.701706867
101	NOS1	cg01207748	Body	OpenSea	other	cluster4	-8.803237788	-0.581153563
102	NOTCH4	cg21993547	Body	OpenSea	other	cluster4	-2.997390659	-0.300500046
103	NR2F2-AS1	cg20650366	Body	OpenSea	other	cluster2	-5.218453586	-0.675415872
104	NWD1	cg13135643	Body	OpenSea	other	cluster2	-2.190030551	-0.322819349
105	OPRK1	cg05027549	Body	N_Shelf	other	cluster4	-5.290551742	-0.431461828
106	PAX8	cg15037451	Body	OpenSea	Proaohancer	cluster4	-5.625094287	-0.461514798
107	PAX8-AS1	cg15037451	Body	OpenSea	Proaohancer	cluster4	-7.692674579	-0.461514798
108	PDE10A	cg11175473	Body	OpenSea	Proaohancer	cluster4	-5.557406824	-0.680181121
109	PGLYRP4	cg15623013	Body	OpenSea	other	cluster4	-10.60959141	-0.606219057
110	PLBD1	cg14376791	Body	OpenSea	other	cluster2	-4.523145018	-0.431025932
111	PLCXD3	cg05638200	Body	OpenSea	other	cluster4	-3.225297589	-0.502156613
112	PLXNA4	cg22531284	Body	OpenSea	other	cluster2	-4.891211543	-0.551524013
113	PPP1R14C	cg06958223	Body	OpenSea	Proaohancer	cluster4	-2.565662053	-0.644159006
114	PRDM16	cg03082523	Body	N_Shore	other	cluster2	-6.791754077	-0.707242597
115	PREX2	cg14532680	Body	OpenSea	other	cluster4	-10.63508389	-0.571037309
116	PTGER3	cg01294197	Body	OpenSea	other	cluster2	-4.898633451	-0.495944664
117	PTGIS	cg13806637	Body	OpenSea	other	cluster2	-2.047487207	-0.423616315
118	PYHIN1	cg05738399	Body	OpenSea	other	cluster4	-7.377701107	-0.409132171
119	PZP	cg17944251	Body	OpenSea	Proaohancer	cluster4	-10.06049743	-0.67568179
120	RASIP1	cg26370886	Body	Island	other	cluster2	-2.278544684	-0.630595118
121	RGS6	cg18127398	Body	OpenSea	Proaohancer	cluster4	-10.10616055	-0.597372944
122	RNASET2	cg25258033	Body	N_Shore	other	cluster2	-2.170398292	-0.427419107
123	ROS1	cg10647214	Body	OpenSea	other	cluster4	-6.493267699	-0.371461176
124	RSPO1	cg11305072	Body	OpenSea	other	cluster2	-8.292062792	-0.782214096
125	RYR1	cg15804426	Body	S_Shore	other	cluster2	-2.023069812	-0.528821952
126	RYR3	cg24364120	Body	OpenSea	other	cluster4	-3.923955812	-0.656485641
127	SCARA5	cg16913114	Body	OpenSea	other	cluster2	-11.91124157	-0.624070631

128	SEC14L5	cg00835521	Body	Island		other	cluster2	-2.31968093	-0.789701701
129	SELP	cg06812356	Body	OpenSea		other	cluster2	-9.899375854	-0.68137366
130	SFMBT2	cg07763467	Body	OpenSea		Proaohancer	cluster4	-4.445168357	-0.603936225
131	SGPP2	cg06379883	Body	OpenSea		other	cluster4	-2.444547676	-0.373947342
132	SIGLEC14	cg16034991	Body	OpenSea		other	cluster4	-6.060556094	-0.488900404
133	SLC22A25	cg16358817	Body	OpenSea		other	cluster2	-6.110716631	-0.587915735
134	SLC22A3	cg01739295	Body	S_Shelf		other	cluster4	-6.23474208	-0.367394147
135	SLC22A7	cg26558036	Body	OpenSea		other	cluster4	-5.915685939	-0.474405486
136	SLC2A9	cg03841832	Body	OpenSea		Proaohancer	cluster4	-4.250372963	-0.531214706
137	SLC45A2	cg11241413	Body	OpenSea		other	cluster4	-2.160018509	-0.333246577
138	SLC47A1	cg11317447	Body	N_Shore		other	cluster2	-3.882905872	-0.427110637
139	SLC6A2	cg03519481	Body	OpenSea		other	cluster2	-4.809599978	-0.572840924
140	SLC9A9	cg10212985	Body	OpenSea		other	cluster4	-5.117449039	-0.475452425
141	SMOC2	cg05621218	Body	OpenSea		Proaohancer	cluster4	-12.77089614	-0.684143316
142	SNTG2	cg07107859	Body	OpenSea		other	cluster4	-7.485636961	-0.647821064
143	SPON1	cg24556573	Body	OpenSea		other	cluster4	-4.753541917	-0.428973671
144	SPTA1	cg16041062	Body	OpenSea		other	cluster4	-4.054303504	-0.706543543
145	ST8SIA2	cg08289525	Body	S_Shore		other	cluster4	-3.29161419	-0.425272998
146	STAB2	cg01726939	Body	OpenSea		other	cluster2	-5.106138627	-0.79774046
147	SULF2	cg08998048	Body	OpenSea		other	cluster2	-4.188128241	-0.470600428
148	SYT6	cg07510237	Body	OpenSea		other	cluster2	-6.235404958	-0.55080872
149	TGM3	cg20306032	Body	Island		other	cluster4	-13.55197272	-0.329348042
150	THEG	cg23463127	Body	N_Shore		other	cluster2	-3.039316407	-0.393052875
151	TNFRSF1B	cg15526535	Body	OpenSea		Proaohancer	cluster2	-2.790133055	-0.545088259
152	TNXB	cg26695758	Body	Island		Proaohancer	cluster2	-7.662277296	-0.501691155
153	TPRXL	cg23145243	Body	OpenSea		other	cluster2	-5.113903708	-0.745091011
154	TRPV6	cg24444878	Body	OpenSea		other	cluster4	-9.048459539	-0.442952544
155	TSHZ2	cg16015829	Body	OpenSea		other	cluster2	-4.114480203	-0.498319678
156	TSPEAR-AS1	cg10054014	Body	S_Shelf		other	cluster2	-2.806380102	-0.522166922

157	TTC22	cg22167738	Body	OpenSea	other	cluster2	-7.14556679	-0.598868001
158	UBQLN3	cg10517815	Body	OpenSea	other	cluster2	-6.797627606	-0.59952975
159	VIPR2	cg19965595	Body	OpenSea	other	cluster2	-8.122989009	-0.782881028
160	WDFY4	cg09632868	Body	OpenSea	other	cluster2	-10.55092383	-0.38406045
161	XDH	cg22597494	Body	OpenSea	other	cluster2	-2.209166677	-0.35933585
162	ZBTB16	cg00387390	Body	OpenSea	other	cluster2	-12.92982609	-0.573175456

Group IV

S.No.	hgnc_symbol	IlmnID	Refgrp	Relation_to_UCSC_CpG_Island	Proaohancer	Cluster	MCC13vsNSk_LogFC	MCC13_meth
1	ABI3	cg06952307	Promoter	OpenSea	Proaohancer	cluster3	-5.191439894	0.402032868
2	ACOX2	cg16587010	Promoter	OpenSea	Proaohancer	cluster1	-7.083793215	0.320814984
3	ACSS1	cg02086310	Promoter	S_Shore	Proaohancer	cluster1	-3.029420844	0.326983435
4	ACVRL1	cg25749512	Promoter	Island	Proaohancer	cluster1	-6.99064495	0.541912755
5	ADAM33	cg11858114	Promoter	Island	Proaohancer	cluster3	-6.071528346	0.377520626
6	ADORA2A-AS1	cg11210890	Promoter	Island	Proaohancer	cluster1	-3.132426568	0.626833583
7	ADRA1A	cg10647509	Promoter	Island	Proaohancer	cluster3	-2.483378276	0.331470037
8	ANGPT1	cg11745019	Promoter	OpenSea	Proaohancer	cluster1	-2.427123452	0.593041845
9	ANKK1	cg16158779	Promoter	Island	Proaohancer	cluster3	-7.629417475	0.559339397
10	ANKRD29	cg03354908	Promoter	S_Shore	Proaohancer	cluster1	-5.053140524	0.372180438
11	AQP1	cg26923410	Promoter	OpenSea	Proaohancer	cluster1	-8.241200687	0.355027377
12	ASB2	cg13485310	Promoter	OpenSea	Proaohancer	cluster1	-2.674175551	0.485714292
13	BHMT	cg02286091	Promoter	OpenSea	Proaohancer	cluster1	-4.035808156	0.607314303
14	BLK	cg02034205	Promoter	OpenSea	Proaohancer	cluster1	-4.447278865	0.354444201
15	BLNK	cg07397033	Promoter	OpenSea	Proaohancer	cluster1	-7.723440246	0.464784852
16	C1QTNF9	cg05820861	Promoter	Island	Proaohancer	cluster1	-3.508952755	0.359450876
17	C2orf40	cg14535980	Promoter	N_Shore	Proaohancer	cluster1	-8.045101902	0.402204839
18	CALCRL	cg21350115	Promoter	OpenSea	Proaohancer	cluster1	-4.408339313	0.678156705
19	CCDC60	cg10573232	Promoter	Island	Proaohancer	cluster1	-5.289401681	0.633804431

20	CD200R1	cg26754426	Promoter	OpenSea	Proaohancer	cluster3	-6.454597935	0.494367217
21	CD300A	cg12331932	Promoter	OpenSea	Proaohancer	cluster1	-5.460406841	0.460640404
22	CD36	cg24275848	Promoter	OpenSea	Proaohancer	cluster1	-5.713796078	0.507568754
23	CD8A	cg12606911	Promoter	Island	Proaohancer	cluster1	-2.285790142	0.319493972
24	CFTR	cg25509184	Promoter	OpenSea	Proaohancer	cluster1	-7.964028486	0.71769267
25	CHL1	cg13925692	Promoter	Island	Proaohancer	cluster3	-5.365942256	0.306912344
26	CLEC14A	cg06284231	Promoter	Island	Proaohancer	cluster1	-10.87608994	0.49688694
27	CLEC3B	cg25576364	Promoter	OpenSea	Proaohancer	cluster1	-9.864577914	0.676352811
28	CMKLR1	cg22927613	Promoter	OpenSea	Proaohancer	cluster1	-9.582447909	0.524662851
29	CMTM2	cg08032924	Promoter	Island	Proaohancer	cluster3	-2.592702757	0.422768106
30	COX7A1	cg24929925	Promoter	Island	Proaohancer	cluster1	-3.781411325	0.653974101
31	CPXM2	cg16197717	Promoter	Island	Proaohancer	cluster3	-11.41475718	0.58495459
32	CRIP1	cg26110883	Promoter	Island	Proaohancer	cluster3	-2.045441757	0.433513233
33	CSRP3	cg05895618	Promoter	OpenSea	Proaohancer	cluster1	-3.760739688	0.355539413
34	CWH43	cg22826333	Promoter	Island	Proaohancer	cluster3	-11.22671018	0.478524995
35	DDO	cg14956327	Promoter	OpenSea	Proaohancer	cluster1	-2.784499405	0.426395827
36	DES	cg03583111	Promoter	Island	Proaohancer	cluster1	-9.641371855	0.741090161
37	DGKA	cg07679948	Promoter	Island	Proaohancer	cluster1	-2.994248633	0.616111689
38	DLEU7	cg08274637	Promoter	Island	Proaohancer	cluster1	-2.590699396	0.73312552
39	DNAJC15	cg12201399	Promoter	N_Shore	Proaohancer	cluster3	-3.521216542	0.529575203
40	ELOVL7	cg16298547	Promoter	N_Shore	Proaohancer	cluster3	-3.388253546	0.521546503
41	EMP2	cg27178401	Promoter	OpenSea	Proaohancer	cluster1	-2.706409849	0.639518548
42	EREG	cg19308222	Promoter	OpenSea	Proaohancer	cluster1	-8.000762175	0.581161018
43	FAM107A	cg23827531	Promoter	OpenSea	Proaohancer	cluster3	-7.386704113	0.377206173
44	FAM135B	cg26005485	Promoter	N_Shore	Proaohancer	cluster3	-4.727958718	0.319426993
45	FAM160A1	cg16008351	Promoter	OpenSea	Proaohancer	cluster3	-3.15965088	0.578536694
46	FAM20A	cg15761609	Promoter	S_Shore	Proaohancer	cluster3	-4.827826735	0.608246842
47	FAM87B	cg16535257	Promoter	OpenSea	Proaohancer	cluster1	-3.956733942	0.72101362
48	FAXDC2	cg23566333	Promoter	OpenSea	Proaohancer	cluster1	-3.754695895	0.33368466

49	FBXL22	cg01447951	Promoter	N_Shelf		Proaohancer	cluster3	-2.477911729	0.451719147
50	FERD3L	cg25691167	Promoter	Island		Proaohancer	cluster1	-5.190673611	0.527009063
51	FERMT1	cg07718903	Promoter	S_Shore		Proaohancer	cluster1	-3.803294529	0.548505197
52	FILIP1	cg26466508	Promoter	OpenSea		Proaohancer	cluster3	-4.207945688	0.385883096
53	FMOD	cg26987645	Promoter	OpenSea		Proaohancer	cluster1	-9.432768296	0.562319615
54	FNDC1	cg09107912	Promoter	Island		Proaohancer	cluster1	-3.21338818	0.777395068
55	FOXI2	cg02523640	Promoter	Island		Proaohancer	cluster3	-4.868959146	0.456377888
56	GALM	cg05604112	Promoter	OpenSea		Proaohancer	cluster1	-4.368852285	0.797015821
57	GFRA2	cg15794228	Promoter	Island		Proaohancer	cluster1	-8.304035164	0.596209028
58	GIMAP5	cg02955377	Promoter	OpenSea		Proaohancer	cluster1	-9.606427393	0.344607895
59	GLIPR1L1	cg24234651	Promoter	OpenSea		Proaohancer	cluster3	-3.304669722	0.409581619
60	GNGT2	cg05026650	Promoter	OpenSea		Proaohancer	cluster3	-3.751168987	0.402032868
61	GRIA1	cg17166762	Promoter	OpenSea		Proaohancer	cluster3	-2.227249102	0.484675837
62	GRM7	cg02037791	Promoter	N_Shore		Proaohancer	cluster3	-2.768586706	0.672614068
63	GSTM1	cg24506221	Promoter	Island		Proaohancer	cluster1	-10.1807532	0.728076258
64	GSTM5	cg24467349	Promoter	OpenSea		Proaohancer	cluster1	-8.855270114	0.622827767
65	GSX2	cg10248878	Promoter	N_Shore		Proaohancer	cluster3	-2.85604305	0.55216218
66	H6PD	cg26754508	Promoter	S_Shelf		Proaohancer	cluster1	-2.483114726	0.583596746
67	HCK	cg17508991	Promoter	N_Shore		Proaohancer	cluster1	-4.972980298	0.873527464
68	HCLS1	cg02167021	Promoter	OpenSea		Proaohancer	cluster1	-6.559955279	0.615692356
69	HIST1H4F	cg12260798	Promoter	N_Shore		Proaohancer	cluster1	-6.702493463	0.767554971
70	ITIH5	cg01634132	Promoter	OpenSea		Proaohancer	cluster3	-13.96463455	0.47036298
71	ITM2B	cg04657044	Promoter	N_Shore		Proaohancer	cluster1	-2.712031714	0.563393047
72	JDP2	cg10101773	Promoter	N_Shore		Proaohancer	cluster1	-2.064962475	0.417252205
73	KCNE3	cg17718679	Promoter	Island		Proaohancer	cluster1	-4.298806081	0.515003982
74	KCNJ15	cg08257579	Promoter	OpenSea		Proaohancer	cluster1	-5.518962057	0.32026907
75	KLHDC7B	cg17174023	Promoter	Island		Proaohancer	cluster1	-2.229077528	0.650615125
76	KLHL31	cg00536792	Promoter	OpenSea		Proaohancer	cluster1	-3.517351136	0.581119536
77	KRT222	cg23776012	Promoter	OpenSea		Proaohancer	cluster3	-4.788991722	0.428562226

78	KRT72	cg22200736	Promoter	S_Shore	Proaohancer	cluster1	-9.334215548	0.421899016
79	LDLRAD4	cg18511326	Promoter	OpenSea	Proaohancer	cluster1	-2.1861089	0.662153154
80	LGI1	cg07793207	Promoter	OpenSea	Proaohancer	cluster3	-7.191441844	0.380068288
81	LHCGR	cg20156659	Promoter	Island	Proaohancer	cluster1	-8.033862346	0.432799161
82	LINC01354	cg13023668	Promoter	OpenSea	Proaohancer	cluster1	-6.440887622	0.651346777
83	LRG1	cg11502375	Promoter	N_Shelf	Proaohancer	cluster1	-6.919770168	0.539500094
84	LRRC3B	cg18404374	Promoter	N_Shore	Proaohancer	cluster3	-3.844822492	0.352725187
85	LTC4S	cg05408831	Promoter	N_Shore	Proaohancer	cluster3	-3.739302686	0.793134479
86	LUM	cg21957925	Promoter	OpenSea	Proaohancer	cluster3	-5.805446814	0.412773205
87	LY96	cg13213009	Promoter	OpenSea	Proaohancer	cluster1	-3.808540648	0.467476855
88	MACC1	cg08705973	Promoter	OpenSea	Proaohancer	cluster3	-9.299965512	0.454780125
89	MCHR1	cg21342728	Promoter	OpenSea	Proaohancer	cluster3	-4.010403991	0.31976038
90	MEGF11	cg23451023	Promoter	S_Shore	Proaohancer	cluster1	-2.207812263	0.509131633
91	MGST2	cg11124069	Promoter	OpenSea	Proaohancer	cluster1	-2.517343345	0.572012182
92	MT1M	cg18909924	Promoter	Island	Proaohancer	cluster1	-4.912066697	0.468026277
93	NDRG2	cg23552977	Promoter	N_Shore	Proaohancer	cluster1	-5.630076058	0.432517547
94	NKAPL	cg18675097	Promoter	Island	Proaohancer	cluster1	-4.393448229	0.752961338
95	NMU	cg15112355	Promoter	S_Shore	Proaohancer	cluster1	-2.940060341	0.511179445
96	NOD1	cg06557644	Promoter	OpenSea	Proaohancer	cluster1	-2.539565395	0.431112398
97	NOSTRIN	cg25694915	Promoter	OpenSea	Proaohancer	cluster1	-3.976452929	0.539419052
98	NPBWR1	cg26205771	Promoter	N_Shore	Proaohancer	cluster3	-2.644819524	0.778305276
99	NPY5R	cg15586439	Promoter	N_Shore	Proaohancer	cluster3	-4.438420545	0.467704488
100	NUPR1	cg15745560	Promoter	OpenSea	Proaohancer	cluster1	-3.873027612	0.547079927
101	OSR1	cg09162333	Promoter	Island	Proaohancer	cluster3	-4.103298163	0.48474976
102	PARP15	cg23442853	Promoter	N_Shore	Proaohancer	cluster1	-6.059906337	0.68976955
103	PAX3	cg18352427	Promoter	Island	Proaohancer	cluster1	-10.4127393	0.403148434
104	PCDHB3	cg01925738	Promoter	N_Shore	Proaohancer	cluster1	-2.101217609	0.541780347
105	PGLYRP1	cg26767632	Promoter	S_Shore	Proaohancer	cluster1	-2.756480489	0.470908932
106	PKDREJ	cg06134567	Promoter	Island	Proaohancer	cluster3	-2.170368817	0.506749252

107	PKP1	cg27256468	Promoter	Island		Proaohancer	cluster1	-11.25500202	0.777781884
108	PLA2R1	cg20257553	Promoter	S_Shore		Proaohancer	cluster1	-6.550429366	0.767414143
109	PLSCR4	cg24315815	Promoter	S_Shore		Proaohancer	cluster3	-2.913971107	0.494124777
110	PPFIBP2	cg04723364	Promoter	N_Shore		Proaohancer	cluster1	-3.174718271	0.539724195
111	RANBP3L	cg21870145	Promoter	OpenSea		Proaohancer	cluster1	-3.829811773	0.519196264
112	RASAL3	cg05504085	Promoter	OpenSea		Proaohancer	cluster3	-9.036000306	0.464850399
113	RASSF9	cg27035734	Promoter	OpenSea		Proaohancer	cluster3	-3.91242177	0.666849026
114	RAX	cg00814752	Promoter	S_Shore		Proaohancer	cluster3	-3.142028671	0.340035626
115	RFTN2	cg23334662	Promoter	OpenSea		Proaohancer	cluster3	-4.176117721	0.688236566
116	RFX8	cg27255239	Promoter	S_Shore		Proaohancer	cluster1	-3.739190244	0.38217908
117	RHOD	cg15693066	Promoter	Island		Proaohancer	cluster1	-3.990281114	0.44890748
118	RNF180	cg23008153	Promoter	N_Shore		Proaohancer	cluster3	-3.796494524	0.331696876
119	S100A5	cg19808620	Promoter	OpenSea		Proaohancer	cluster1	-3.386206468	0.443996507
120	SGCD	cg12904904	Promoter	OpenSea		Proaohancer	cluster1	-2.955032158	0.37233704
121	SLC10A6	cg15881238	Promoter	OpenSea		Proaohancer	cluster3	-8.320777539	0.526158948
122	SLC13A5	cg26782108	Promoter	Island		Proaohancer	cluster3	-3.162260465	0.528890812
123	SLC22A8	cg11200963	Promoter	OpenSea		Proaohancer	cluster1	-6.094042734	0.520798269
124	SLC25A2	cg05845376	Promoter	Island		Proaohancer	cluster1	-4.529581596	0.569234793
125	SLC34A2	cg09157302	Promoter	N_Shore		Proaohancer	cluster3	-7.384442609	0.30640536
126	SLC38A11	cg05339727	Promoter	OpenSea		Proaohancer	cluster3	-6.599869604	0.364139096
127	SLC38A4	cg21428710	Promoter	OpenSea		Proaohancer	cluster3	-3.870708078	0.691387523
128	SOD3	cg03577139	Promoter	OpenSea		Proaohancer	cluster1	-11.4961738	0.563312655
129	SORBS1	cg20959701	Promoter	OpenSea		Proaohancer	cluster1	-2.301449518	0.648272791
130	SP8	cg27191852	Promoter	N_Shelf		Proaohancer	cluster1	-5.933811731	0.434016837
131	SPHKAP	cg00423153	Promoter	S_Shore		Proaohancer	cluster1	-4.248398921	0.642570273
132	STAR	cg09630404	Promoter	OpenSea		Proaohancer	cluster1	-4.322470632	0.481524889
133	STAT5A	cg08246644	Promoter	N_Shore		Proaohancer	cluster1	-2.293816376	0.762652353
134	STEAP4	cg07719679	Promoter	OpenSea		Proaohancer	cluster1	-10.83623227	0.334583239
135	SULT1C2	cg25838818	Promoter	OpenSea		Proaohancer	cluster1	-2.647120414	0.339557363

136	SYCE1	cg20087519	Promoter	Island		Proaohancer	cluster1	-4.254963892	0.748126387
137	TACC2	cg23331156	Promoter	OpenSea		Proaohancer	cluster1	-3.237201309	0.64914875
138	TAL1	cg06955484	Promoter	N_Shore		Proaohancer	cluster1	-5.257840483	0.590426078
139	TBX15	cg05329979	Promoter	Island		Proaohancer	cluster1	-2.658930984	0.691077662
140	TBX5	cg06911121	Promoter	Island		Proaohancer	cluster1	-9.673229552	0.598830805
141	TBX5-AS1	cg16517851	Promoter	Island		Proaohancer	cluster1	-9.989169603	0.809698396
142	TDRD9	cg06289566	Promoter	Island		Proaohancer	cluster1	-3.143435368	0.600802375
143	THRB	cg14974411	Promoter	OpenSea		Proaohancer	cluster1	-3.531437352	0.346027726
144	TLR2	cg14581949	Promoter	N_Shore		Proaohancer	cluster3	-3.188347972	0.303976655
145	TMC1	cg14327393	Promoter	OpenSea		Proaohancer	cluster1	-2.584275535	0.5301041
146	TMEM144	cg18366748	Promoter	N_Shore		Proaohancer	cluster1	-2.763016963	0.55558698
147	TMEM196	cg15377585	Promoter	S_Shore		Proaohancer	cluster3	-5.992444523	0.354510747
148	TMEM61	cg08937380	Promoter	Island		Proaohancer	cluster3	-5.708156949	0.351468183
149	TMEM63A	cg10459226	Promoter	S_Shore		Proaohancer	cluster1	-2.206655433	0.328116446
150	TNNT2	cg00003287	Promoter	OpenSea		Proaohancer	cluster1	-5.268949381	0.313221311
151	TNS1	cg03323067	Promoter	OpenSea		Proaohancer	cluster1	-2.911920624	0.52261418
152	TRIM55	cg23322523	Promoter	OpenSea		Proaohancer	cluster3	-3.826531525	0.34381061
153	TRIM61	cg14038058	Promoter	OpenSea		Proaohancer	cluster3	-4.551366934	0.646159926
154	UPB1	cg12492885	Promoter	Island		Proaohancer	cluster1	-4.246555851	0.682642296
155	VNN1	cg01052291	Promoter	OpenSea		Proaohancer	cluster3	-6.298287536	0.411371593
156	VWA5A	cg10399199	Promoter	OpenSea		Proaohancer	cluster1	-2.140092657	0.324984961
157	WFDC1	cg19051527	Promoter	N_Shore		Proaohancer	cluster1	-4.441887624	0.527029333
158	WFIKKN2	cg10408284	Promoter	OpenSea		Proaohancer	cluster1	-6.438383997	0.62955538
159	WNT3	cg02017282	Promoter	Island		Proaohancer	cluster1	-2.172308968	0.583201484
160	ZC3H12D	cg09313931	Promoter	OpenSea		Proaohancer	cluster3	-4.989484676	0.779546199
161	ZNF154	cg01268824	Promoter	S_Shore		Proaohancer	cluster1	-3.420761937	0.678207268

Supplemental Table 4: List of MCC specific genes related to MCPyV status and regulated by DNA methylation						
	gene name	probes	Met. Status	Location	Relation to CpG Island	Proaohancer
1	ABCA4	cg19196920	hypo	Body	OpenSea	other
2	CCR1	cg01297500	hypo	Body	OpenSea	other
3	CIT	cg14132388, cg14312334, cg20840795, cg24973993, cg26097051	hypo	Body	S_Shore	Proaohancer
4	CYP3A7	cg18504691	hypo	Body	OpenSea	other
5	HTR5A	cg13892088	hypo	Promoter	S_Shore	Proaohancer
6	INPP5D	cg14134851, cg27165095	hypo	Body	OpenSea	other
7	KCNK12	cg01886809	hypo	Body	N_Shelf	other
8	MRPL3	cg15810640	hypo	Promoter	S_Shore	Proaohancer
9	TIGIT	cg09755142	hypo	Body	OpenSea	other
10	UBE2I	cg06703573, cg19439687, cg26827394	hypo	Body, and/or Promoter	Island	Proaohancer
